# The E3 ligase MKRN2 prevents DRiP accumulation in stress granules and maintains granulostasis

**DOI:** 10.1038/s44319-026-00832-2

**Published:** 2026-06-15

**Authors:** Emmanuel Amzallag, Yehuda M Danino, Valentina Secco, Serena Carra, Eran Hornstein

**Affiliations:** 1 Department of Molecular Genetics, Weizmann Institute of Science, Rehovot, Israel; 2 Department of Molecular Neuroscience, Weizmann Institute of Science, Rehovot, Israel; 3 Department of Biomedical, Metabolic and Neural Sciences, University of Modena and Reggio Emilia, Modena, Italy

**Keywords:** Post-translational Modifications & Proteolysis, Proteomics, RNA Biology

## Abstract

Stress granules (SGs) are transient cytoplasmic biomolecular condensates that play a role in the cellular response to proteotoxic stress. It has been previously shown that ubiquitination regulates SG dynamics; however, the specific mechanisms by which ubiquitin affects SGs are not fully understood. Here, using proximity proteomics, we discover that the recruitment of several E3 ubiquitin ligases, VCP cofactors and HSP70 to SGs is dependent on the activity of the E1 ubiquitin ligase UBA1. The RNA-binding E3 ubiquitin-protein ligase Makorin 2 (MKRN2) is strongly depleted from SGs in the absence of UBA1 function. MKRN2 promotes both the proper formation of SGs and their disassembly following stress recovery, by preventing the accumulation of misfolding-prone defective ribosomal products (DRiPs) within SGs. Therefore, MKRN2 is a novel regulator of SGs that mediates the maintenance of granulostasis.

## Synopsis

UBA1-dependent recruitment of the E3 ubiquitin ligase MKRN2 to stress granules maintains granulostasis by preventing the accumulation of defective ribosomal products (DRiPs) within these biomolecular condensates.

- Several E3 ubiquitin ligases, VCP cofactors and HSP70 are recruited to SGs in UBA1-dependent manner.
- The E3 ligase MKRN2 maintains granulostasis by preventing the accumulation of misfolding-prone DRiPs into SGs and is essential for both proper SG assembly during heat stress and efficient disassembly during recovery.

## Abstract (web summary)

UBA1-dependent recruitment of the E3 ubiquitin ligase MKRN2 to stress granules maintains granulostasis by preventing the accumulation of defective ribosomal products (DRiPs) within these biomolecular condensates.

## Graphical abstract

**Figure:**
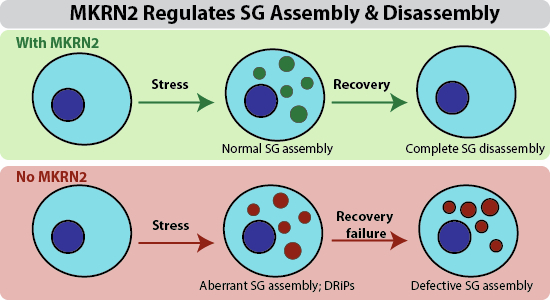

**Figure:**
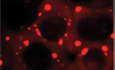

## Introduction

Stress granules (SGs) are biomolecular condensates that form via a process of liquid-liquid phase separation (LLPS) of biomacromolecules (Protter and Parker, 2016). SG assembly is initiated by a variety of stressors, including heat stress, oxidative stress, osmotic stress, and ER stress, during which the cell activates the integrated stress response (ISR) (Sidrauski et al, 2015). Phosphorylation of eIF2α initiates the ISR, resulting in polysome disassembly and global shutdown of translation. This releases mRNA molecules from ribosomes and engages a variety of RNA-binding proteins (RBPs) to form multivalent interactions with both RNA and with other proteins via RNA-binding domains (RBDs) and intrinsically disordered domains (IDD), respectively, resulting in the formation of discrete SGs. Upon recovery from stress, SGs rapidly disassemble, and their dissolution allows mRNAs to be redirected to translation.

 SGs can fail to disassemble and persist for a long time, affecting the cells’ ability to restore translation, as well as the retro-translocation in the nucleus of RBPs (Protter and Parker, 2016). Several factors can delay SG disassembly, including the accumulation of misfolded and damaged proteins and the impairment of the protein quality control system. The latter refers to molecular chaperones and protein degradation systems that cooperate to detect unfolded/misfolded proteins and promote their clearance (Hipp et al, 2019). Amongst the misfolded and potentially toxic proteins that can accumulate inside SGs, changing their dynamic liquid-like properties are disease-linked mutated RBPs such as TDP-43, FUS or hnRNPA1 (Gordon et al, 2019; Baron et al, 2013; Gui et al, 2019), as well as defective ribosomal products (DRiPs), which are truncated proteins released from disassembling polyribosomes. DRiPs are rapidly polyubiquitinated for degradation by the proteasome system as well as by autophagy (Schubert et al, 2000). Amongst the chaperones that participate in the maintenance of SG dynamics are the ATP-dependent chaperone complexes. These chaperone complexes reduce DRiP accumulation inside SGs (Seguin et al, 2014; Ganassi et al, 2016; Alberti et al, 2017). Accordingly, the activity of HSP70, VCP, and also the autophagic machinery, thus helps surveil stress-induced DRiPs and maintain SGs functioning properly, in a process termed granulostasis (Alberti et al, 2017).

Until now, the maintenance of granulostasis under stress has been largely associated with the activity of specific molecular chaperones, namely the HSP70/HSPB8/BAG3 and VCP/UFD1L/PLAA complexes (Seguin et al, 2014; Ganassi et al, 2016). The ubiquitin-proteasome system (UPS) is a major component of cellular protein degradation and mediates the conjugation of ubiquitin onto target molecules marked for proteasomal degradation. While ubiquitination and SUMOylation, have been shown to impact SG dynamics as well (Maxwell et al, 2021; Marmor-Kollet et al, 2020; Verde et al, 2025) it is unknown if they regulate DRiPs accumulation in SGs.

Active ubiquitination is required for proper SG disassembly (Maxwell et al, 2021; Gwon et al, 2021; Tolay & Buchberger, 2021; Yang et al, 2023; Pan et al, 2025; Xu et al, 2023; Keiten-Schmitz et al, 2020; Xie et al, 2018; Jeon et al, 2024; Wang et al, 2019; Turakhiya et al, 2018); however, the specific E3 ligases that maintain proper SG dynamics and granulostasis are only partially known. For example, the E3 ligase TRIM21 mediates K63-linked G3BP1 ubiquitination, and SG condensation and the HECT-domain E3 ligases ITCH and NEDD4L regulate SG disassembly (Yang et al, 2023; Pan et al, 2025). However, whether specific E3 ligases regulate the clearance of DRiPs from SGs has not yet been explored.

Here, we report ubiquitination-dependent changes in SG proteomic composition that led to the identification of Makorin 2 (MKRN2) as an important SG-engaged E3 ligase that regulates SG dynamics and the sequestration of stress-induced DRiPs into SGs.

## Results and discussion

### Ubiquitination affects stress granule dynamics and composition

**Figure 1:**
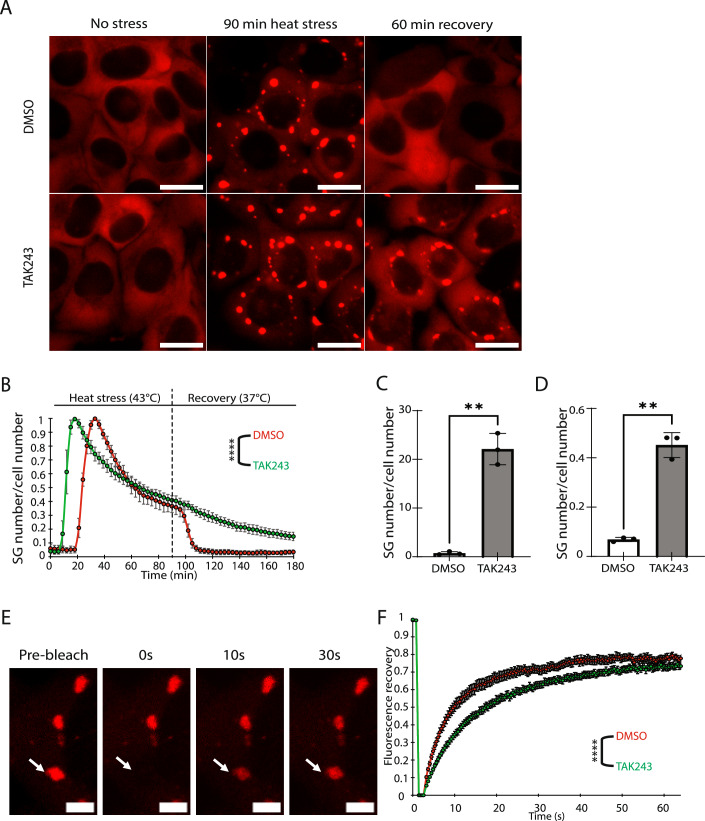
Ubiquitination affects stress granule dynamics and biophysical properties. (**A**) Representative images of U2OS cells overexpressing G3BP1-mCherry with G3BP1/2 KO background, without (DMSO) or with TAK243 (1 uM) after 90 min of heat stress at 43 °C (HS) or during recovery at 37 °C for 1 h. Scale bar = 20 um. (**B**) Quantification of average SG number/cell number as a function of time. Data were normalized internally to the time point with maximal SG assembly. ~1000 SG counted per biological repeat. Nine fields per condition. Data from one experiment out of three biological repeats (Appendix Fig. S1). *p* < 0.0001. Two-way ANOVA with repeated measures. Error bars = s.d. (**C**) Quantification of SG number/cell number at 15 min of HS. Average of three biological repeats shown. *p* = 0.0070. Welch’s *t*-test. Error bars = s.d. (**D**) Quantification of SG number/cell number at 60 min recovery. Average of three biological repeats shown. *p* = 0.0048. Welch’s *t*-test. Error bars = s.d. (**E**) A temporal image series from a FRAP experiment on G3BP1-mCherry U2OS cells under heat stress. The arrow indicates the photobleached SG. Scale bar = 5 um. (**F**) Quantification of fluorescence recovery after photobleaching in U2OS cells without or with TAK243. Data from one biological repeat out of three is shown (Appendix Fig. S2A). ~20 SGs were counted per experiment. *p* < 0.0001. Two-way ANOVA with repeated measures. Error bars = s.e.m. *p* value * <0.05; ** <0.01, ****<0.0001. Source data are available online for this figure.

It was recently shown that UBA1 activity is required for proper SG disassembly (Maxwell et al, 2021; Gwon et al, 2021; Tolay and Buchberger, 2021, 2022). However, a detailed characterization of how SGs and the UPS interact has not been achieved. Using G3BP1-mCherry expressing U2OS cells, in which the endogenous G3BP1 and G3BP2 genes were knocked out, (Kedersha et al, 2016) we studied the full SG assembly and disassembly cycle, induced by heat stress (HS; 43 °C followed by stress recovery at 37 °C; Fig. 1A,B). Inhibition of the ubiquitin-activating enzyme UBA1 (ubiquitin-like modifier-activating enzyme 1) by TAK243 (1 μM) facilitated SG assembly, while delaying their dissolution (Fig. 1A–C; Appendix Fig. S1). Treatment with TAK243 coupled with HS caused SGs to nucleate and begin assembling earlier than in HS-treated cells (Fig. 1B,C). SG disassembly following recovery from heat stress, at 37 °C, was also delayed (Fig. 1A,B,D; Appendix Fig. S1).

To determine whether UBA1 activity affects SG liquidity, we performed fluorescence recovery after photobleaching (FRAP) on cells under HS, following UBA1 inhibition by TAK243. Inhibition of UBA1 delayed fluorescence recovery dynamics of G3BP1-mCherry, relative to HS-treated cells (Fig. 1E,F; Appendix Fig. S2A). These observations indicate that UBA1 activity is required for maintaining SG liquidity, consistent with previous reports (Gwon et al, 2021).

**Figure 2:**
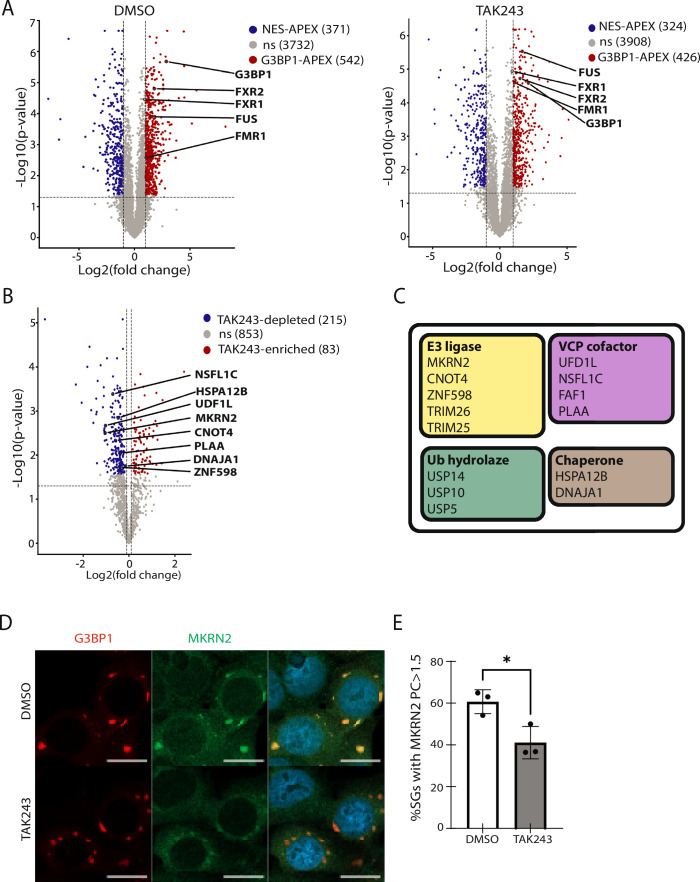
Ubiquitination affects stress granule proteomic composition. (**A**) Volcano plot of proteins enriched at the proximity of G3BP1-APEX relative to NES-APEX cytoplasm control (x-axis = Log2 fold change) and statistical significance (student’s *t*-test with FDR; y-axis = Log10 *p* value). G3BP1/2 KO cells expressing G3BP1-APEX were used to label SG proteins. Four biological replicates per group. (**B**) Volcano plot displaying the ubiquitin-dependent enrichment in SG proteins. Eighty-three proteins were enriched and 215 proteins depleted from SGs following TAK243 treatment. (student’s *t*-test with FDR; x-axis = LogFC; y-axis = log10 *p* value). Several E3 ligases, ubiquitin hydrolases, and chaperones are depleted. Four biological replicates per group. (**C**) Table showing proteostasis factors depleted from SGs upon inhibition of ubiquitination. (**D**) Representative images of G3BP1-mCherry cells treated with TAK243 and stained for endogenous MKRN2. Scale bar = 20 um. (**E**) Quantification of the partition coefficient of MKRN2 in SGs in cells treated with DMSO or TAK243. The proportion of SGs with PC >1.5 is shown. Average of three biological repeats, ~1000 SGs per repeat. *p* = 0.0279. Welch’s two-sided *t*-test. Error bars = s.d. *p* value * = <0.05; **<0.01, ****<0.0001. Source data are available online for this figure.

To characterize the composition of SGs and identify players of the ubiquitination pathway that are involved in maintaining SG dynamics, we used APEX proximity labeling coupled to mass spectrometry (APEX-MS). G3BP1/2 KO U2OS cells expressing G3BP1-APEX were used to label SG proteins, while U2OS cells expressing nuclear export signal (NES)-APEX were used to label the surrounding cytoplasm. Heat stress was induced (43 °C; 90 min), UBA1 was inhibited with TAK243, and proximity labeling was induced with biotin phenol (500 μM) and a short hydrogen peroxide pulse (1 mM, 60 s). Western blot was done to ensure efficient biotinylation activity in all samples (Appendix Fig. S2B). Biotin-labeled proteins were isolated with streptavidin magnetic beads and analyzed by mass spectrometry. Samples without biotin phenol were used to identify and subtract non-APEX-derived biotinylation background. Peptide reads were mapped to proteins with the MaxQuant search engine (v1.6.6.0). Relative to the NES cytoplasmic control, ~1000 proteins interacting with G3BP1-APEX were identified, including previously-reported SG proteins (Fig. 2A), providing confidence that our protocol effectively isolated SG proteomes. To understand which proteins are associated with SGs in a UBA1-dependent manner, we next compared the HS-induced SG proteomes without or with TAK243 (Fig. 2B; Appendix Fig. S3A,B). This provided a list of proteins either enriched (83 proteins) or depleted (215 proteins) from SGs upon inhibition of UBA1 (Student’s *T*-test, FDR <0.05; Fig. 2C). These data are provided in Datasets EV1–EV4. Amongst the proteins that associate with SGs in a UBA1-dependent manner, we report HSPA12, DNAJA1, and the VCP cofactors, UFD1L, NSFL1C, FAF1, and PLAA. Interestingly, these chaperones were previously implicated in promoting SG disassembly by preventing aberrant accumulation of stress-damaged proteins (Gwon et al, 2021; Ganassi et al, 2016). Ubiquitin hydrolases and E3 ligases were also associated with SGs in a UBA1-dependent manner, including the RNA-binding ubiquitin ligases (RBULs) MKRN2, CNOT4, TRIM25, TRIM26 and ZNF598. MKRN2 exhibited the highest level of UBA1-dependent SG association since it was the most highly depleted E3 ligase upon treatment with TAK243 (Log2FC = 2; Fig. 2B; Dataset EV1). Importantly, examination of the basal enrichment level of each of these E3 ligases in the APEX data showed that MKRN2 was not the E3 with the highest level of basal SG enrichment (MKRN2 = ~1.37 Log2FC, ZNF598 = ~1.5 Log2FC, CNOT4 = 0.58 Log2FC, TRIM25 = 0.52 Log2FC, and TRIM26 = 0.58 Log2FC; Dataset EV3), supporting the interpretation that MKRN2’s localization to SG is dependent on UBA1 activity and is not simply due to a generally higher level of SG localization.

### The E3 ligase MKRN2 affects SG dynamics

**Figure 3:**
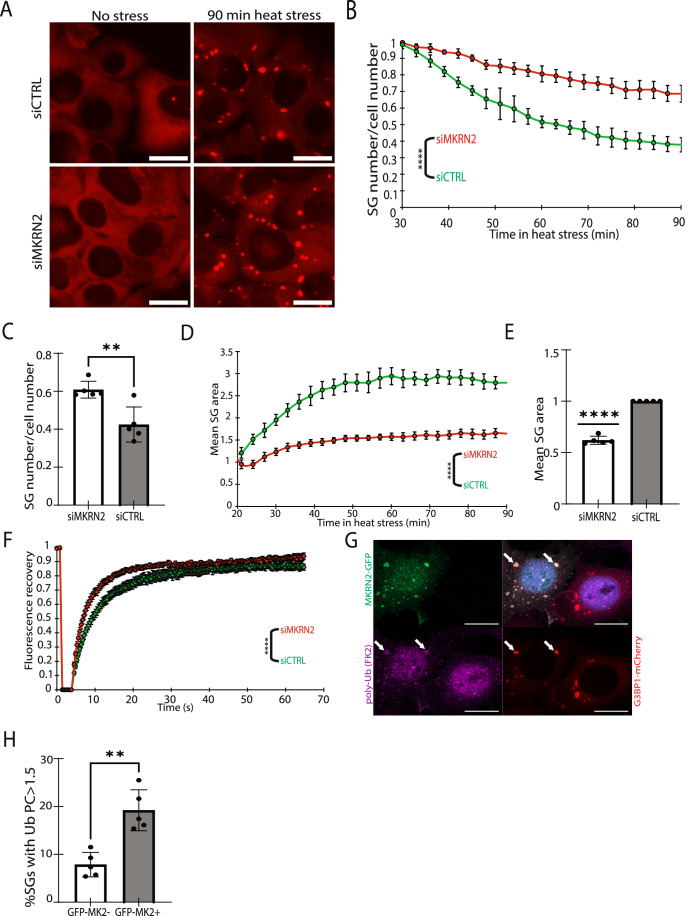
MKRN2 promotes SG assembly and increases SG ubiquitin content. (**A**) Representative images of G3BP1-mCherry cells with siMKRN2 vs siCtrl before HS and at 90 min HS at 43 °C. Scale bar = 20 um. (**B**) Quantification of average SG number/cell number from 30 to 90 min HS at 43 °C. Data were normalized internally to the time point with maximal SG assembly. ~1000 SG counted per experiment. Nine fields per condition. Data from one biological repeat out of five independent experiments. *p* < 0.0001. Two-way ANOVA with repeated measures. Error bars = s.d. (**C**) Quantification of SG number/cell number at 90 min HS. Average of five biological repeats shown. *p* = 0.0077. Welch’s *t*-test. Error bars = s.d. (**D**) Average SG area over the 90 min HS at 43 °C. Data from one experiment out of five biological repeats. (Appendix Fig. S8). *p* < 0.0001. Two-way ANOVA with repeated measures. Error bars = s.d. (**E**) Average SG area at 90 min HS at 43 °C. Data were internally normalized to siCtrl. The average of five biological repeats is shown. *p* = 0.0001. One-sample *t*-test. Error bars = s.d. (**F**) Quantification of fluorescence recovery after photobleaching in U2OS cells with either siMKRN2 or siCTRL. Data from one biological repeat out of three is shown (Appendix Fig. S2B). ~20 SGs were counted per experiment. *p* < 0.0001. Two-way ANOVA with repeated measures. Error bars = s.e.m. (**G**) Confocal micrographs of G3BP1-mCherry/MKRN2-GFP U2OS cells stained for poly-ubiquitin (FK2). Scale bar = 20 um. (**H**) FK2 partition coefficient quantification in SGs from either MKRN2-GFP+ vs MKRN2-GFP− cells within the same coverslip. The proportion of SGs with PC >1.5 is shown. Average of five biological repeats shown. >1000 SGs per repeat. Error bars = s.d. *p* = 0.0017. Welch’s two-sided *t*-test. *p* value * = <0.05; **<0.01, ****<0.0001. Source data are available online for this figure.

**Figure 4:**
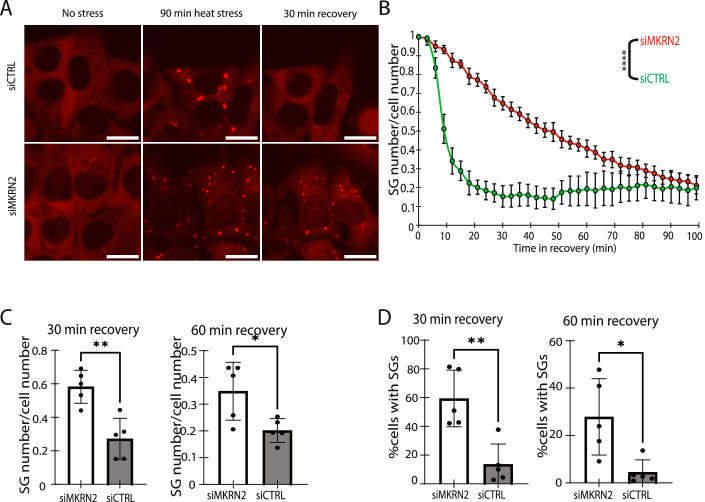
MKRN2 promotes SG disassembly. (**A**) Representative images of G3BP1-mCherry cells with siMKRN2 vs siCtrl before HS, at 90 min HS at 43 °C and at 30 min recovery at 37 °C. Scale bar = 20 um. (**B**) Live imaging quantification of average SG number/cell number during recovery at 37 °C. Data were internally normalized to 90 min of HS. Nine sites per condition. Data from one experiment out of five biological repeats. *p* < 0.0001. Two-way ANOVA with repeated measures. Error bars = s.d. (**C**) Quantification of SG number/cell number at 30 min (*p* = 0.0025) or 60 min (*p* = 0.0353) recovery for siMKRN2 vs siCTRL. Average of five biological repeats shown. Welch’s *t*- test. Error bars = s.d. (**D**) Quantification of percent cells with SGs at 30 min (*p* = 0.0036) or 60 min (*p* = 0.0286) recovery for siMKRN2 vs siCTRL. Average of five biological repeats shown. Welch’s *t*-test. Error bars = s.d. *p* value * = <0.05; **<0.01, ****<0.0001. Source data are available online for this figure.

The human genome encodes two E1s and ~30 E2 enzymes However, ~600 E3 ligases are conferring substrate specificity in targeting specific proteins and cellular pathways (Clague et al, 2015). To determine whether UBA1-sensitive RBULs control SG dynamics, we knocked down CNOT4, ZNF598, and MKRN2 in U2OS cells that were subjected to HS, followed by recovery (43 °C, 90 min -> 37 °C). Knockdown of CNOT4, ZNF598, or MKRN2 all impacted SG dynamics to varying degrees (Appendix Fig. S4A,B). Efficient KD was validated by RT-qPCR and western blot (Appendix Figs. S4C–E and S5A,B). MKRN2 depletion affected both SG assembly and disassembly, with cells forming smaller SGs that took longer to disassemble upon recovery (Figs. 3 and 4). ZNF598 similarly affected SG size but had a smaller impact on disassembly. CNOT4 had a minimal effect on SG assembly, with a modest effect on their disassembly (Appendix Fig. S4A,B). We orthogonally validated the UBA1-dependent SG localization of the most SG-depleted E3 ligase, MKRN2, by immunofluorescence in U2OS cells fixed after 90 min HS, without or with TAK243 (Fig. 2D,E). In agreement with APEX-MS results, endogenous MKRN2 exhibited decreased SG partitioning in response to TAK243 treatment (Fig. 2E).

Previously published SG proximity labeling approaches identified MKRN2 as an SG component but did not find CNOT4 and ZNF598 (Youn et al, 2018; Markmiller et al, 2018). Based on our and others’ mass spectrometry data, indicating MKRN2 as the most highly SG-depleted E3 upon TAK243 treatment and its strongest impact on SG dynamics, we focused mechanistic studies on MKRN2. The analysis of endogenous MKRN2 confirms its distribution is predominantly cytoplasmic, with a minor nuclear presence under both basal and HS conditions (Appendix Fig. S6A,B). This localization pattern aligns with established literature (Shin et al, 2017; Wolf et al, 2020). To determine whether MKRN2 is necessary for inducing ubiquitin localization to SGs, we KD MKRN2 and measured ubiquitin partitioning to SGs using the FK2 antibody (Appendix Fig. S7A,B). We could not detect a significant decrease in FK2 partitioning to SGs following MKRN2 KD, pointing to compensatory activity of other SG-engaged E3 ligases. To investigate the role of MKRN2 E3 catalytic activity in its SG localization, we created an MKRN2 RING mutant, designed to be ubiquitination-deficient by mutating all critical Cys and His residues. This mutant displayed a significant reduction in partitioning into SGs, compared to the wild-type protein, suggesting that a functional RING domain is important for the efficient recruitment/retention of MKRN2 within these biomolecular condensates (Appendix Fig. S7C,D). Furthermore, no difference in VCP partitioning to SGs was detected in response to MKRN2 KD (Appendix Fig. S7E), consistent with the unaffected total SG ubiquitin content (Appendix Fig. S7A,B).

Using live imaging, we investigated the consequences of MKRN2 KD on SG dynamics under HS. SG assembly under HS typically involves a reduction in SG numbers over time, as smaller condensates coalesce to form larger but fewer SGs (Chernov et al, 2009; Hu et al, 2023; Van Treeck and Parker, 2019; Seguin et al, 2014). The knockdown of MKRN2 resulted in a higher number of SGs at 90 min of HS (Fig. 3A–C), and the average size of SGs was almost half as small compared to the control (Fig. 3D,E; Appendix Fig. S8). These data suggest that SGs fail to coalesce and mature upon MKRN2 KD. Having established that MKRN2 depletion affects SG number and size, we then measured their liquid-like properties by performing FRAP experiments, using the mobility of G3BP1 as readout. Consistently, FRAP analysis demonstrated that MKRN2 KD resulted in a slight increase in G3BP1 fluorescence recovery rate in SGs, which is interpreted as increased SG liquidity (Fig. 3F,) and may be associated with decreased coalescence into dense and mature SGs (Van Treeck and Parker, 2019; Wheeler et al, 2016).

We next tested the impact of MKRN2 on SG disassembly following stress removal. MKRN2 KD delayed the disassembly of SGs, and the effect was pronounced 30 and 60 min after cessation of HS (Fig. 4A–D; Appendix Fig. S9). In response to sodium arsenite stress, no effect on SG disassembly was observed following recovery, although the cells did form significantly smaller SGs. (Appendix Fig. S10A–D). Therefore, MKRN2 is required for maintaining proper SG disassembly under HS.

To further test the impact of MKRN2 on SG dynamics, we overexpressed MKRN2-GFP, which exhibited both nuclear and cytoplasmic localization, and was recruited to SGs under heat stress (Fig. 3G). Thus, similar to endogenous MKRN2, MKRN2-GFP localized to both the nuclear and cytoplasmic compartments but displayed a stronger cytoplasmic bias (Appendix Fig. S11A,B). Additionally, overexpression of MKRN2-GFP significantly enhanced the recruitment of mono- and polyubiquitinated proteins to SGs, compared to non-expressing neighboring cells in the same polyclonal mosaic cultures (Fig. 3G,H). Together, these data indicate that MKRN2 promotes SG coalescence during HS and SG disassembly following recovery from HS and may be associated with increased ubiquitination within SGs, as suggested by co-localization. While MKRN2 is sufficient to drive ubiquitin partitioning into SGs, it is not strictly necessary, perhaps due to functional redundancy with alternative E3 ligases (Appendix Fig. S7B).

### MKRN2 regulates the accumulation of DRiPs in SGs

**Figure 5:**
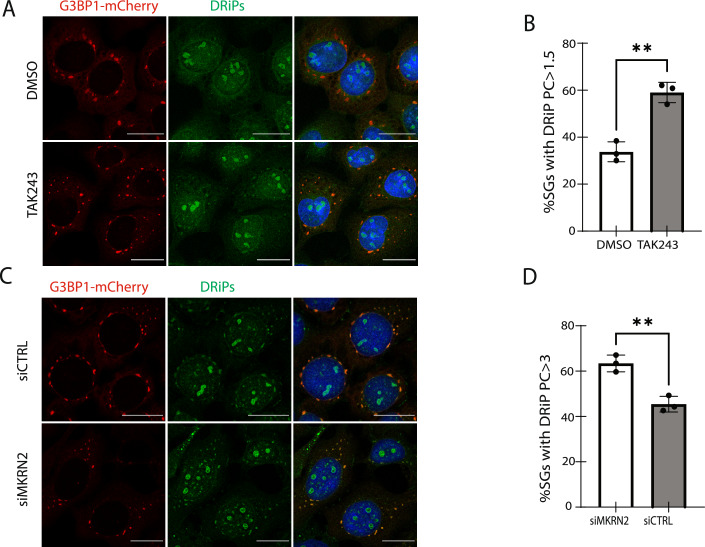
Ubiquitination is required for preventing DRiP accumulation in SGs. (**A**) Representative images of DMSO vs TAK243 treated G3BP1-mCherry expressing U2OS cells, treated with 25 μM OP-puro to label DRiPs and heat-stressed at 43 °C for 60 min to induce SGs. Scale bar = 20 um. (**B**) Quantification of the partition coefficient of DRiPs in SGs from cells treated with either DMSO or TAK243. The proportion of SGs with PC >1.5 is shown. Average of three biological repeats. *p* = 0.002. Welch’s *t*-test. Error bars = s.d. (**C**) Representative images of siMKRN2 vs siCTRL G3BP1-mCherry U2OS cells treated with 25 μM OP-puro to label DRiPs and heat-stressed at 43 °C. Scale bar = 20 um. (**D**) Quantification of the partition coefficient of DRiPs in SGs from cells treated with either siMKRN2 or siCTRL. The proportion of SGs with PC >3 is shown. Average of three biological repeats. *p* = 0.0036. Welch’s *t*-test. Error bars = s.d. **<0.01. Source data are available online for this figure.

DRiPs are truncated peptides that are prone to aggregation and DRiPs ubiquitination affects their solubility and clearance (Schubert et al, 2000; Seguin et al, 2014; Qian et al, 2006). Proteostasis impairment causes aberrant DRiP accumulation inside SGs and impairs SG disassembly. To test the idea that ubiquitination impacts a subset of DRiPs in SGs, we induced SG formation by HS in the absence or presence of the UBA1 inhibitor TAK243. Concomitantly, we treated the cells with O-propargyl puromycin (OP-puro, 25 uM), which is incorporated into nascent polypeptide chains, causing polysome disassembly and the emergence of DRiPs. We observed increased partitioning of OP-puro labeled DRiPs in SGs upon inhibition of UBA1 relative to control (Fig. 5A,B). These data suggest that UBA1 activity prevents the accumulation of DRiPs inside SGs. To understand whether the accumulation of a fraction of DRiPs into SGs is regulated by MKRN2, we then performed a similar analysis in proficient and MKRN2-deficient cells. We measured increased partitioning of OP-puro labeled DRiPs in SGs upon knockdown of MKRN2, relative to control (Fig. 5C,D). For the MKRN2 KD cells, a cutoff of 3 was chosen to specifically highlight the population of SGs that are strongly enriched with DRiPs, which was significantly higher in MKRN2 KD cells compared to control (Fig. 5D). Therefore, these data suggest that MKRN2 regulates the accumulation of a subset of DRiPs into SGs, plausibly via ubiquitination activity.

Together, our study provides evidence for a new role for UBA1 activity and for the E3 ligase MKRN2 in the control of SG dynamics and DRiPs turnover. Using APEX proximity labeling and mass spectrometry, we demonstrate that UBA1 activity regulates SG proteomic composition and functions under heat stress by controlling the recruitment of several molecular regulators of proteostasis into SGs.

Specifically, UBA1 activity regulates the localization of several proteostasis-related proteins to SGs, including four VCP cofactors (UFD1L, PLAA, NSFL1C and FAF1), two of which (UFD1L and PLAA) have been shown to regulate SG dynamics and sequestration of DRiPs (Seguin et al, 2014). This suggests that ubiquitin-binding VCP cofactors are recruited to SGs through their interaction with ubiquitinated proteins within these condensates. Furthermore, the localization of the heat shock protein HSP70 (HSPA12B) and its co-chaperone DNAJA1 to SGs is also regulated by UBA1 activity. This finding is consistent with similar behavior observed under sodium arsenite stress (Pan et al, 2025) and is likely to influence the accumulation of DRiPs in SGs (Ganassi et al, 2016).

Several E3 ubiquitin ligases were depleted from SGs in the absence of UBA1 activity, namely MKRN2, ZNF598, CNOT4, TRIM25, and TRIM26, whereas the E3 RNF20 was enriched in SGs. A detailed study of the E3 ligase MKRN2 demonstrated UBA1 activity-dependent partitioning of endogenous MKRN2 into SGs. This was further supported by decreased SG partitioning of RING-mutant MKRN2 compared to WT. Our experiments demonstrate that MKRN2 localization to SGs depends at least partially on its ubiquitination capacity, but recruitment via other mechanisms, such as its binding affinity for RNA or ubiquitinated proteins, cannot be ruled out. All in all, this suggests the intriguing possibility that the localization of a subset of E3 ligases into SGs is linked to their capacity to ubiquitinate target proteins.

Pan et al, reported enrichment of several HECT-domain E3 ligases in SGs following inhibition of UBA1 by TAK243 (Pan et al, 2025), suggesting that this process is specific and potentially regulated in a stress- or protein-specific manner. Notably, this study utilized oxidative stress by sodium arsenite treatment, while our current study focused on heat stress as the cellular stress model. The identification of different SG-engaged E3 ligases thus suggests that oxidative and heat stress each activate distinct ubiquitination pathways, contributing to our understanding of how different stress types differ biologically. Consistent with this, MKRN2 was dispensable for arsenite SG disassembly, but was required for heat stress SG disassembly. Additionally, all five of the E3 ligases identified in our study as exhibiting UBA1-dependent SG association are RBULs (Guseva et al, 2024; Williams et al, 2019; Garzia et al, 2017; Kulkarni et al, 2025), and their capacity to bind RNA may be particularly intriguing for SG-engaged E3s.

Endogenous and ectopically expressed MKRN2 were both localized to SGs. MKRN2 has a functional role in regulating SG dynamics under heat stress, and its knockdown results in more numerous and smaller SGs, as well as a significant delay in SG disassembly following recovery from stress. The combined role of MKRN2 on both SG assembly and disassembly is consistent with published literature on VCP. This phenotype was also seen upon VCP depletion, which resulted in the formation of smaller “dispersed SGs” (Seguin et al, 2014) as well as delayed SG disassembly (Buchan et al, 2013; Gwon et al, 2021; Tolay and Buchberger, 2021). Consistent with our findings regarding VCP depletion (Seguin et al, 2014), we propose that the unregulated accumulation of DRiPs within early SG foci following MKRN2 depletion prevents their proper fusion and growth into normal, mature SGs. Accordingly, immature SGs are more diffusive and recover faster than mature SGs after photobleaching (Wheeler et al, 2016).

We also show that UBA1 activity regulates a subset of DRiP homeostasis in SGs, suggesting that ubiquitination is required for preventing excessive DRiPs accumulation in SGs under heat stress. We further show that the RBUL MKRN2 similarly prevents accumulation of DRiPs in SGs, suggesting that it may execute ubiquitin-mediated extraction of DRiPs from SGs. We note that our experiments cannot delineate whether ubiquitination of DRiPs by MKRN2 occurs inside SGs or in the cytoplasm. However, the finding that MKRN2 is enriched in SGs under stress, coupled with the fact that its overexpression increases SG ubiquitin content, suggests that MKRN2 controls DRiP accumulation into SGs via polyubiquitination of SG-localized substrates. Notably, we cannot conclusively determine that MKRN2-mediated prevention of DRiP accumulation in SGs results from its own ubiquitination-ligase activity, since we have not analyzed MKRN2 catalytic functions. These experiments should be followed up on in subsequent studies.

VCP activity has also been associated with the prevention of DRiP accumulation in SG. Our results suggest that MKRN2 is dispensable for VCP recruitment to SGs, likely because the total pool of polyubiquitinated proteins, the primary signal for VCP localization, remains largely unchanged in its absence. This functional redundancy of MKRN2 with other E3 ligases, coupled with recently identified SG-engaged E3 ligases, suggests that the SG ubiquitin landscape is complex and is governed by a diverse network of E3 ligases acting on multiple substrate types. In summary, MKRN2 is the first E3 ligase shown to play a role in preventing DRiP accumulation in SGs, thereby tying it to the process of granulostasis previously associated with molecular chaperones such as HSP70 and VCP. The phenotype of smaller, more numerous SGs concomitant with increased DRiP partitioning, also reported upon inhibition of autophagy and VCP function (Seguin et al, 2014; Tolay and Buchberger, 2021; Buchan et al, 2013), suggests that MKRN2 may act in the same pathway.

We note that the SG disassembly phenotype upon MKRN2 knockdown is less pronounced than that observed with TAK243 treatment. Therefore, MKRN2 may target a subset of DRiPs, consistent with the role of additional SG-localized E3 ligases that regulate SG dynamics. In addition, the effect of UBA1 inhibition is expected to be broader than inhibiting a single E3 ligase like MKRN2 and likely affects ubiquitination of a broader range of substrates. For example, K63-linked G3BP1 ubiquitination is known to be required for SG disassembly, which likely contributes to the phenotype seen upon TAK243 treatment. Overall, our results support the conclusion that proper SG dynamics depend on preventing the accumulation of DRiPs in these condensates and suggest that MKRN2 is a novel proteostasis factor regulating this process.

Limitations: The direct MKRN2 substrates and ubiquitin-chain types (K63/K48) are currently unknown. Additionally, we have not demonstrated a conclusive association of MKRN2 catalytic activity with its SG-related functions. In addition, we have not performed APEX proteomics at multiple time points during SG assembly and disassembly and therefore may have missed dynamic recruitment of proteins during the SG life-cycle. In addition, it is interesting to understand if DRiP solubility is dependent on ubiquitination or on specific E3 ligases, such as MKRN2.

In sum, UBA1 activity promotes the recruitment of key SG-engaged chaperones, including HSP70 and VCP, as well as a subset of E3 ubiquitin ligases. We identify the E3 ligase MKRN2 as a novel regulator of SG dynamics and proteostatic function. Prevention of accumulation of DRiPs into SGs requires UBA1 activity and is mediated in part by MKRN2. These insights refine our understanding of how SGs coordinate the triage of stress-damaged proteins with condensate dynamics during proteotoxic stress. Ultimately, defining ubiquitination-dependent regulation of SG composition and function may illuminate new therapeutic avenues for restoring proteostasis in diseases that involve protein aggregation.

## Methods

**Table:** Reagents and tools table

Reagent/resource	Reference or source	Identifier or catalog number
**Experimental models**		
G3BP1/2 KO U2OS cells expressing G3BP1-mCherry	10.1083/jcb.201508028	
G3BP1/2 KO U2OS cells expressing G3BP1-APEX	10.1016/j.molcel.2020.10.032	
U2OS cells expressing NES-APEX	10.1016/j.molcel.2020.10.032	
**Recombinant DNA**		
pcDNA™4/TO	Thermo Fisher	V102020
GFP-pcDNA™4/TO	10.1016/j.molcel.2020.10.032	
GFP-MKRN2-pcDNA™4/TO	This study	Addgene ID 257245
GFP-MKRN2-RINGmut-pcDNA™4/TO	This study	Addgene ID 257246
**Antibodies**		
Mouse anti-ubiquitin (FK2 clone)	Cayman Chemical	14220
Rabbit anti-MKRN2	Sigma-Aldrich	HPA037559-100UL
Rabbit anti-VCP	Abcam	ab155146
Mouse anti-G3BP1	Santa Cruz	sc-365338
Mouse anti-GAPDH	Santa Cruz	sc-47724
Goat anti-Rabbit IgG (H + L) Highly Cross-Adsorbed Secondary Antibody, Alexa Fluor™ 647	Thermo Fisher	A-21245
Rabbit anti-Mouse IgG Recombinant Secondary Antibody, Alexa Fluor™ Plus 488	Thermo Fisher	A56580-50UL
**Oligonucleotides and other sequence-based reagents**		
RT-qPCR primers	This study	Table EV1
Cloning primers	This study	Table EV1
**Chemicals, enzymes and other reagents**		
Streptavidin-HRP	Sigma-Aldrich	RABHRP3-600UL
Tetracycline	Sigma-Aldrich	T7660
Biotin-Tyramide	Iris Biotech	41994-02-9
Hydrogen peroxide	JT Baker	2186-01
Sodium azide	Sigma-Aldrich	S2002
Sodium ascorbate	Alfa Aesar	A17759
Trolox	Sigma-Aldrich	238813
Sodium deoxycholate	Sigma-Aldrich	D6750
cOmplete Protease Inhibitor Cocktail	Roche	4693116001
PhosSTOP	Roche	4906837001
Bradford reagent	Bio-Rad	5000006
Streptavidin magnetic beads	Thermo Fisher	88817
CAS-Block	Thermo Fisher	008120
Fluoroshield™ with DAPI	Thermo Fisher	F6057
DMEM	Sartorius	01-052-1 A
Fetal bovine serum	Sigma	F7524
Penicillin-streptomycin-amphotericin B	Sartorius	03-033-1B
TAK243	Med Chem Express	HY-100487
Lipofectamine RNAiMAX	Thermo Fisher	13778075
Lipofectamine 2000	Thermo Fisher	11668027
siGENOME Human MKRN2 (23609) siRNA - SMARTpool	Dharmacon	M-006960-01-0005
siGENOME Non-targeting pool	Dharmacon	D-001206-13-05
Dimethyl sulfoxide	Sigma-Aldrich	D2650
In-Fusion® Snap Assembly	Takara Clontech	638947
O-Propargyl-Puromycin	Med Chem Express	HY-15680
**Software**		
Imaris v10.0.0	https://imaris.oxinst.com/	
Cellprofiler v4.2.5	https://cellprofiler.org/	
Perseus 2.0.7.0	https://maxquant.net/perseus/	
Maxquant	https://maxquant.org/	
Leica LAS X Office	https://www.leica-microsystems.com/products/microscope-software/p/leica-las-x-ls/	
Zeiss ZEN lite	https://www.zeiss.com/microscopy/en/products/software/zeiss-zen-lite.html	
**Other**		

### Mammalian cell culture

U2OS cells were cultured in growth media consisting of high glucose DMEM (Sartorius 01-052-1A), 10% FBS (Sigma F7524), and 1% penicillin-streptomycin-amphotericin B (Sartorius 03-033-1B) in a 37 °C incubator supplemented with 5% CO_2_. To induce heat stress, cells were placed into a dedicated incubator set to 43 °C supplemented with 5% CO_2_. For UBA1 inhibition, cells were pre-treated with 1-uM TAK243 (Med Chem Express; Cat# HY-100487) prior to heat stress. For siRNA KD, cells were transfected with siRNA (Dharmacon siGENOME SMARTpool) using Lipofectamine RNAiMAX (Cat#: 13778075) and cultured for 72 h before analysis. Cells were routinely tested for mycoplasma contamination.

### Molecular cloning

Molecular cloning of MKRN2-GFP was performed using In-Fusion® Snap Assembly (Cat# 638947; Takara Clontech) and inserted into the pcDNA4.0 vector with puromycin resistance. To create the RING mutant, restriction-free cloning was used. All primers used in cloning are listed in Table EV1. G3BP1-mCherry cells were transfected with Lipofectamine 2000 (Thermo Fisher Scientific, Cat# 11668027) and cultured with 5 ng/ml puromycin for 7 days.

### RNA isolation and RT qPCR

RNA was isolated using the Qiagen RNeasy Mini Kit (Cat# 74104). Reverse transcription was done using High-Capacity cDNA Reverse Transcription Kit (Applied Biosystems; Cat# 4368814), and qPCR was performed using KAPA SYBR® FAST qPCR Master Mix (2X) Kit (KAPA Biosystems; Cat# KK4601). Primers used for qPCR are listed in Table EV1.

### Microscopy

SG live imaging was performed in a Zeiss CD7 widefield microscope equipped with an Axiocam 702 CMOS camera using a 20X air objective with an additional 2X post-magnification (effective magnification of 40X, excitation wavelength 590 nm). G3BP1-mCherry cells were seeded into optical 24-well plates (Cellvis P24-1.5H-N) and placed into the heated microscope incubator chamber (37 °C, 5% CO_2_). After one cycle of imaging, the microscope incubator temperature was increased to 43 °C. After 90 min of heat stress, the temperature was lowered back down to 37 °C to image SG disassembly. SG number and area was analysed using the surfaces feature in Imaris software. Immunofluorescent staining was performed on 12 mm round glass coverslips inside 24-well plates. Immunofluorescent images were captured in a Zeiss LSM900 confocal microscope using a 63X oil objective lens. Analysis of immunofluorescent images was done in Cellprofiler.

### FRAP

G3BP1-mCherry cells were seeded into 3.5 mm dishes with a central coverslip (MatTek P35GC-1.5-14-C). Cells were imaged in a Leica SP8 STED microscope using a 100X objective. To induce heat stress, the microscope incubator temperature was increased to 43 °C, and an objective heater was attached to the objective and heated to 43 °C. Cells were left in the heated microscope incubator chamber for 60 min, and FRAP was performed for 30 min thereafter. To photobleach SGs, a 561 nm laser set to max power was used to bleach the entire SGs. In order to monitor fluorescence recovery, images were collected every 400 ms for 63 s. Fluorescence recovery data were collected using the Leica Las X software. Analysis corrected for background fluorescence intensity and normalized to the pre-bleach SG intensity to calculate the fraction of fluorescence recovery at each time point.

### APEX proximity labeling

G3BP1-APEX or NES-APEX U2OS (Marmor-Kollet et al, 2020) cells were seeded into T125 flasks with 50 ng/ml tetracycline (Sigma T7660) and allowed to incubate for 24 h before performing the APEX experiment. Four technical repeats were used for each condition. Following pretreatment with TAK243, cells were supplemented with 500-uM Biotin-Tyramide (Iris Biotech 41994-02-9) for 90 min to allow for labeling activity. Labeling was then induced by the addition of H_2_O_2_ (JT Baker 2186-01) for 60 s. APEX activity was extinguished with quenching solution (10 mM sodium azide (Sigma cat number S2002), 10 mM sodium ascorbate (Alfa Aesar A17759), 5 mM Trolox (Sigma 238813) in PBS. Cells were then scraped in PBS, centrifuged at 3000×*g* for 10 min at 4 °C, and the cell pellet was lysed in ice-cold RIPA buffer (50 mM Tris-HCl pH8, 150 mM NaCl, 1% NP-40, 0.5% sodium deoxycholate, and 0.1% SDS) supplemented with cOmplete Protease Inhibitor Cocktail (Roche, 4693116001) and PhosSTOP (Roche, 4906837001). Lysates were centrifuged at 15,000×*g* for 10 min at 4 °C, and protein concentration was quantified using the Bradford assay (Bio-Rad 5000006). Streptavidin magnetic beads (Thermo Fisher cat number 88817) were incubated with the protein extracts at a ratio of 100-ul beads per 500-ug protein extract overnight at 4 °C with rotation.

### Liquid chromatography and mass spectrometry

Samples were subjected to on-bead tryptic digestion. The resulting peptides were analysed using nanoflow liquid chromatography (nanoAcquity) coupled to high-resolution, high mass accuracy mass spectrometry (Q-Exactive Plus). Each sample was analysed on the instrument separately in a random order in discovery mode. Raw data were processed with MaxQuant v1.6.6.0. The data were searched with the Andromeda search engine against the human proteome database, appended with common lab protein contaminants and the default modifications. Quantification was based on the LFQ method, based on unique/all peptides.

### Proteomics statistical analysis

The ProteinGroups output table from MaxQuant was imported into Perseus for analysis. First, contaminants were removed. Raw intensity values were log_2_ transformed and missing values imputed for proteins with at least four valid values using an artificial normal distribution with a downshift of 1.8 standard deviations and a width of 0.3 of the original ratio distribution. To identify proteins non-specifically bound to streptavidin beads, a student’s *t*-test with FDR ≤0.05 was used to compare each sample to respective no BP control samples. These non-specific binders were filtered out, and proteins were then compared between G3BP1-APEX and NES-APEX baits. LFQ values were log_2_ transformed, and missing values were imputed for proteins with at least two valid values in at least one group using an artificial normal distribution with a downshift of 1.8 standard deviations and a width of 0.3 of the original ratio distribution. Proteins were then compared between the G3BP1-APEX bait and the NES-APEX bait using a Student’s *t*-test with FDR ≤0.05 to determine which proteins were significantly enriched in proximity to G3BP1 compared to NES. G3BP1-APEX values were then normalized by the mean of their corresponding NES-APEX values. The NES-normalized values were then compared between the TAK243 vs DMSO- treated samples using Student’s *t*-test with FDR ≤0.05.

### Labeling of nascent peptides with OP-puro and a high-content imaging-based assay

Cells were grown on polylysine-coated glass coverslip. Newly synthesized proteins were labeled by incubating the cells with 25 mM *O*-Propargyl-puromycin (OP-puro) for the indicated time points and CuAAC detection of OP-puro labeled DRiPs was performed as previously described, using Alexa Fluor 594 azide (A10270, Life Technologies) (Liu et al, 2012). Cells were next processed for immunofluorescence microscopy. Blocking and incubation with primary and secondary antibodies were performed in PBS containing 3% BSA and 0.1% Triton X-100.

DRiPs enrichment inside SGs was measured using the Scan^R Analysis software (Olympus). SGs were segmented based on the G3BP1 signal using an edge detection algorithm. The mean fluorescence intensity of OP-puro labeled DRiPs was detected in each detected SG and was additionally measured in an area surrounding each SG. The relative enrichment of OP-puro labeled DRiPs in individual SGs was calculated as a ratio of mean fluorescence intensity inside the SG divided by mean intensity in the region surrounding the SG, as previously described (Ganassi et al, 2016).

### Statistical analysis

Statistical analysis of live imaging data was done using repeated-measures ANOVA test with contrast analysis in R. Pairwise comparisons were done using one-sample *t*-test, two-sample *t*-test (two-way) or Welch’s *t*-test (two-way; in cases where variance was not equal between groups) in Graphpad Prism. No blinding was needed since data quantification was performed using automated image analysis software. Sample sizes were chosen based on standard protocols in the field and are consistent with established practices. Statistical *p* values <0.05 were considered significant.

## Supplementary information

Table EV1

Appendix

Peer Review File

Dataset EV1

Dataset EV2

Dataset EV3

Dataset EV4

Source data Fig. 1

Source data Fig. 2

Source data Fig. 3

Source data Fig. 4

Source data Fig. 5

Source data for appendix figs S1-S5

Source data for appendix figs S6-S11

## Data Availability

The datasets generated in this study are available at the following repositories: Imaging data: The microscopy imaging data have been deposited in the BioImage Archive repository with the dataset identifier S-BIAD3204. https://www.ebi.ac.uk/biostudies/bioimages/studies/S-BIAD3204. Proteomics data: The mass spectrometry proteomics data have been deposited to the ProteomeXchange Consortium via the PRIDE partner repository with the dataset identifier PXD076622. The source data of this paper are collected in the following database record: biostudies:S-SCDT-10_1038-S44319-026-00832-2.
