# Supplementary material for: The E3 ligase MKRN2 prevents DRiP accumulation in stress granules and maintains granulostasis
Source: EMBO Rep. 2026 Jun 15;27(15):4238–51. doi: 10.1038/s44319-026-00832-2 (PMC13458048; doi:10.1038/s44319-026-00832-2)
Supplement: Supplementary file 1 [file 44319_2026_832_MOESM2_ESM.pdf]

Appendix

Appendix Figure S1. SG disassembly live imaging repeats with TAK243 1-2

Appendix Figure S2: FRAP repeats 3-4

Appendix Figure S3. APEX samples 5-6

Appendix Figure S4. Live imaging with MKRN2, ZNF598 and CNOT4 7-8

Appendix Figure S5: Expression of G3BP1 following MKRN2 KD 9-10

Appendix Figure S6: Subcellular localization of endogenous MKRN2 11-12

Appendix Figure S7: Ubiquitin, VCP and MKRN2-RING partitioning to SGs 13-14

Appendix Figure S8: SG assembly live imaging repeats with MKRN2 KD 15-16

Appendix Figure S9. SG disassembly live imaging repeats with MKRN2 KD 17-18

Appendix Figure S10: Live imaging with sodium arsenite 19-20

Appendix Figure S11: Subcellular localization of overexpressed MKRN2-GFP 21-22

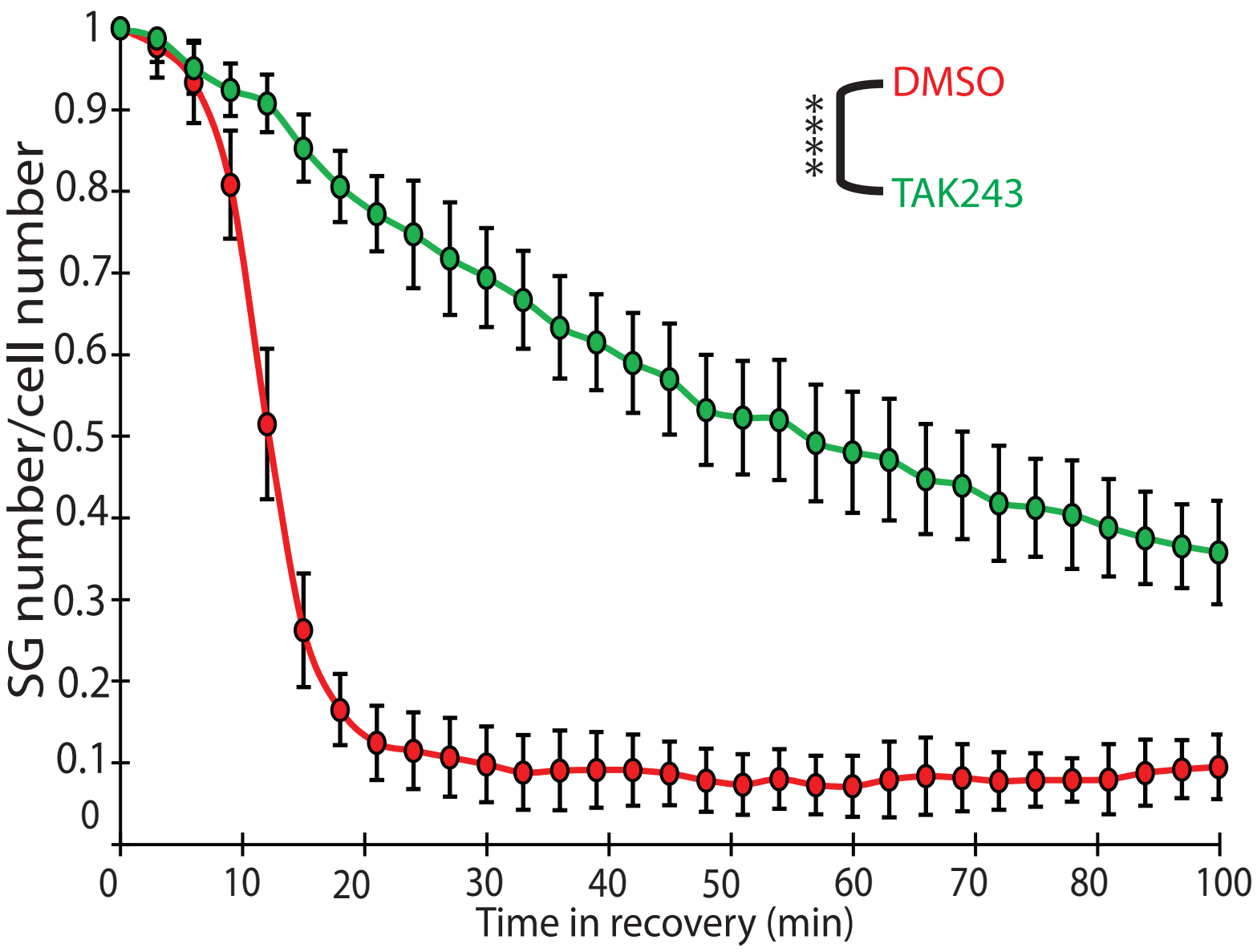
SG number/cell number
1
0.9
0.8
0.7
0.6
0.5
0.4
0.3
0.2
0.1
0
0 10 20 30 40 50 60 70 80 100
Time in recovery (min)
DMSO
****
TAK243

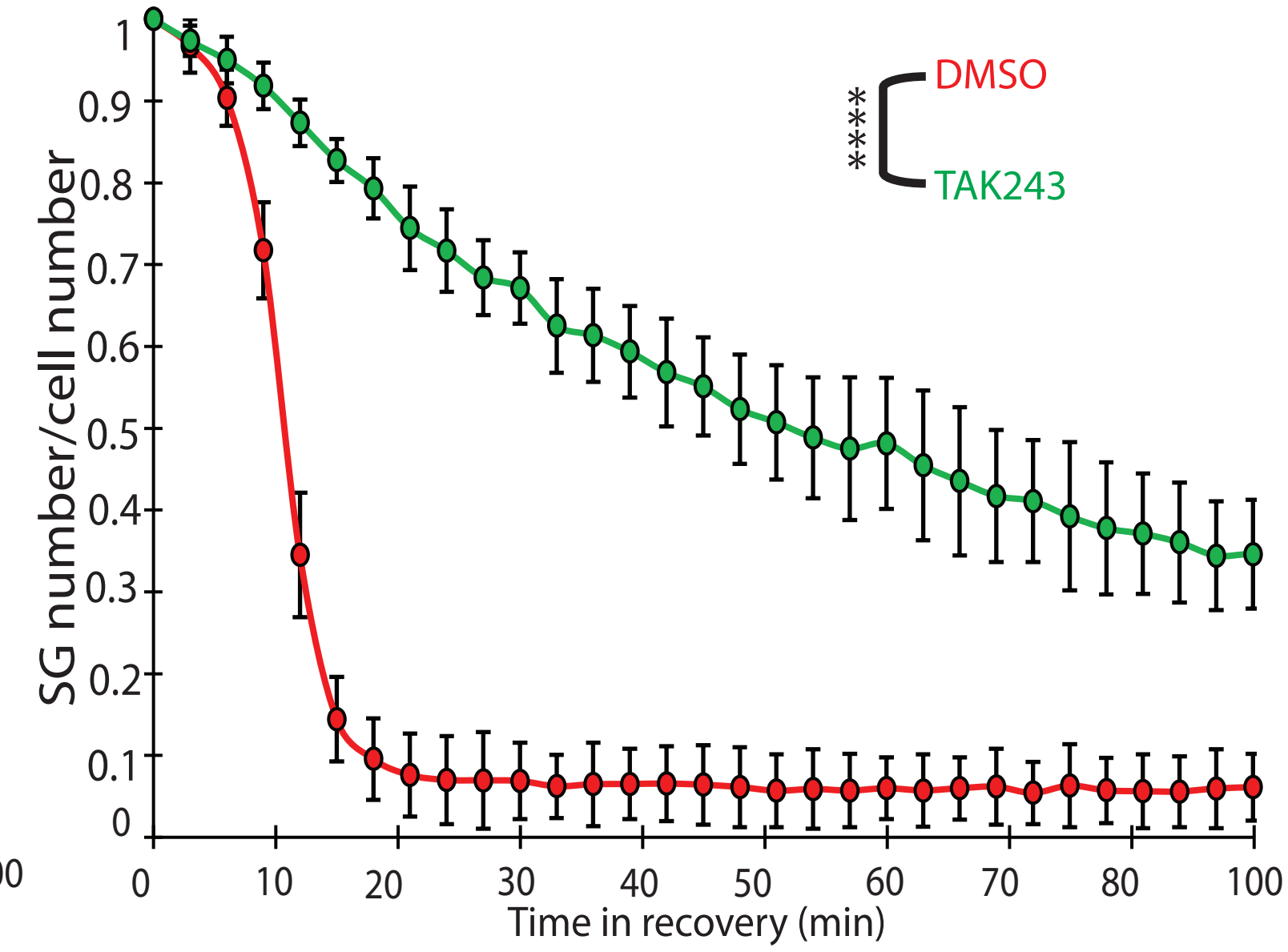
SG number/cell number
1
0.9
0.8
0.7
0.6
0.5
0.4
0.3
0.2
0.1
0
0 10 20 30 40 50 60 70 80 100
Time in recovery (min)
DMSO
****
TAK243

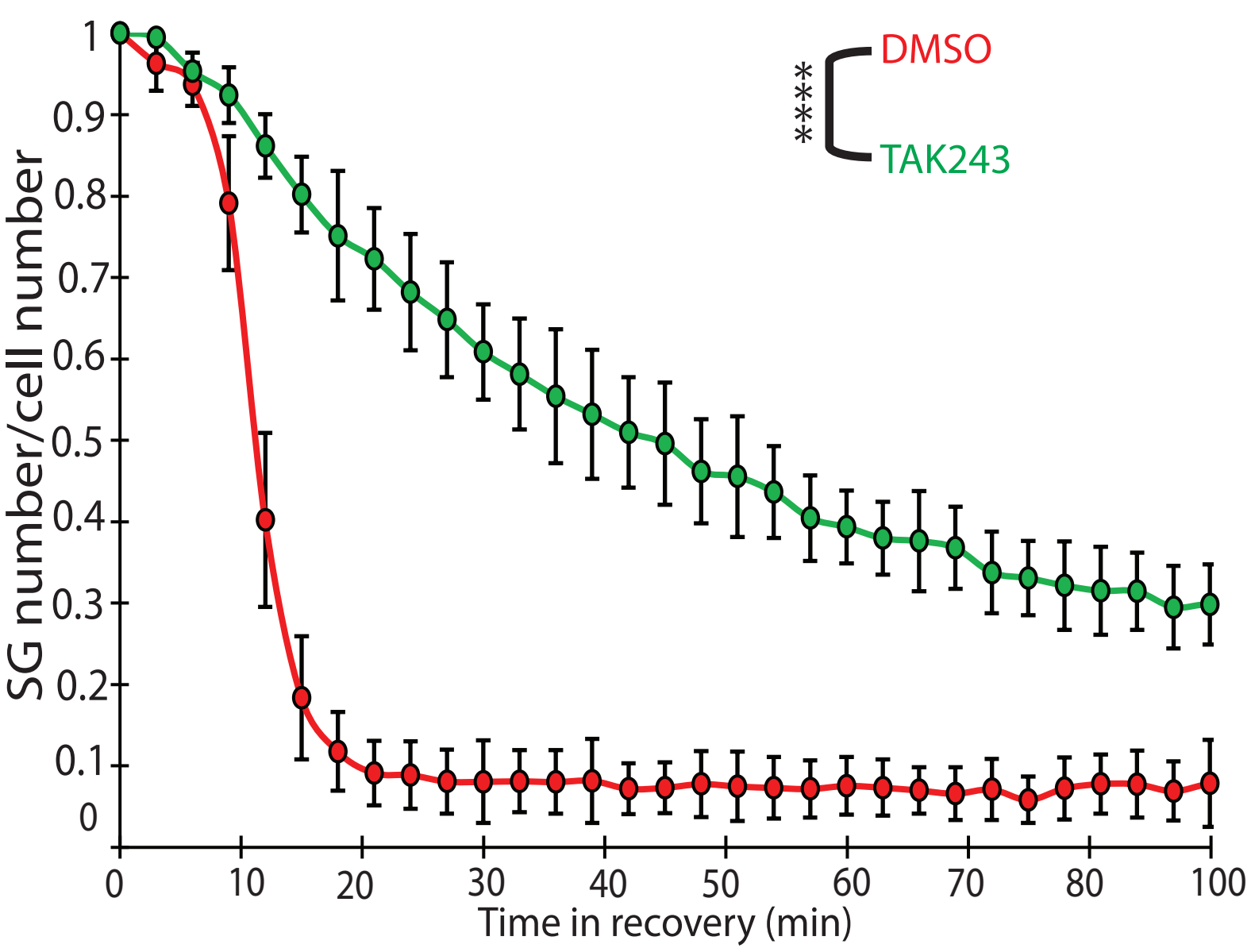
SG number/cell number
1
0.9
0.8
0.7
0.6
0.5
0.4
0.3
0.2
0.1
0
0 10 20 30 40 50 60 70 80 100
Time in recovery (min)
DMSO
****
TAK243

Appendix Figure S1. SG disassembly live imaging repeats with TAK243. Live imaging quantification of SG number/cell number during recovery at 37°C. Data were internally normalized to 90 min of HS. 9 sites per condition. Three biological repeats shown. Two-way ANOVA with repeated measures. Error bars = s.d. **** = <0.0001.

A

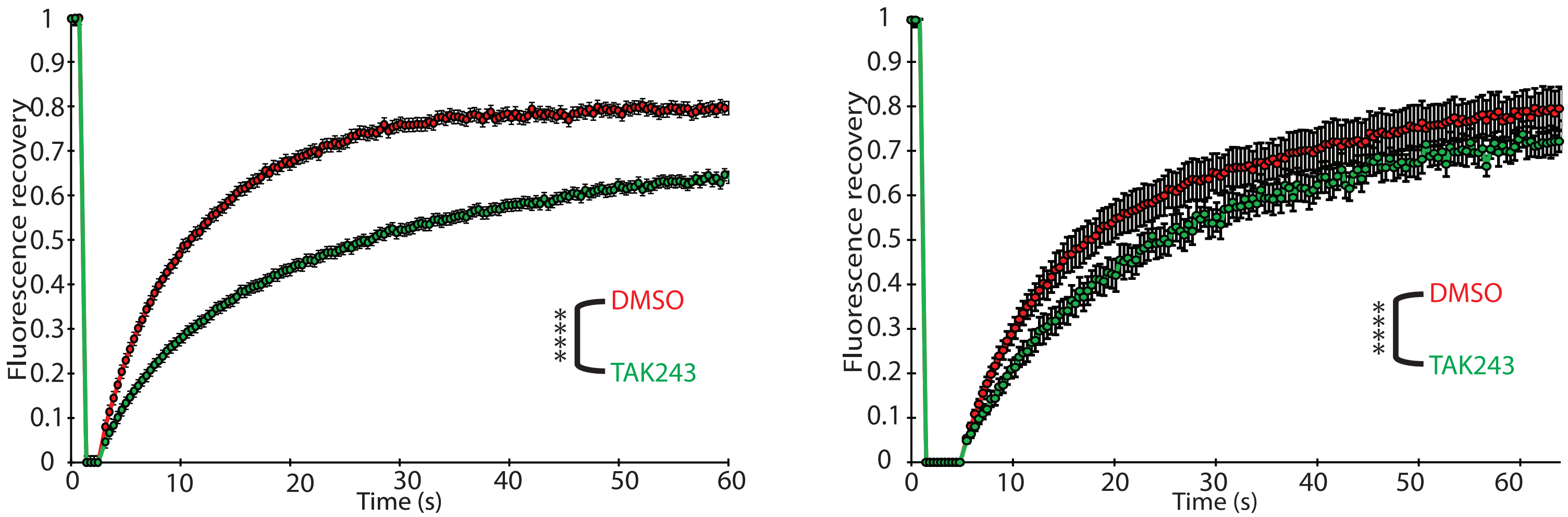

Fluorescence recovery
Time (s)
DMSO
TAK243
****
Fluorescence recovery
Time (s)
DMSO
TAK243
****

B

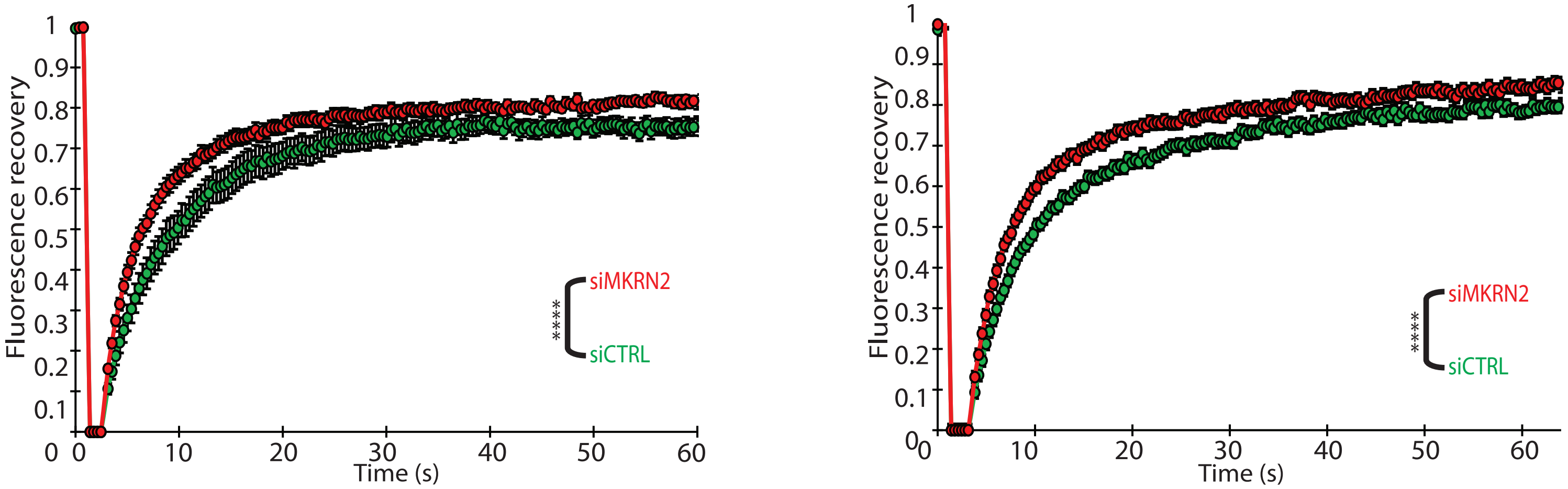

Fluorescence recovery
Time (s)
siMKRN2
siCTRL
****
Fluorescence recovery
Time (s)
siMKRN2
siCTRL
****

Appendix Figure S2: FRAP repeats. **A.** Quantification of fluorescence recovery after photobleaching in TAK243 vs control cells. Two biological repeats shown (third repeat in Fig. 1F). ~20 SGs per repeat. Two way ANOVA with repeated measures. Error bars = s.e.m. **B.** Quantification of fluorescence recovery after photobleaching in TAK243 vs control cells. Two biological repeats shown (third repeat in Fig. 3F). ~20 SGs per repeat. Two way ANOVA with repeated measures. Error bars = s.e.m. **** = <0.0001.

A

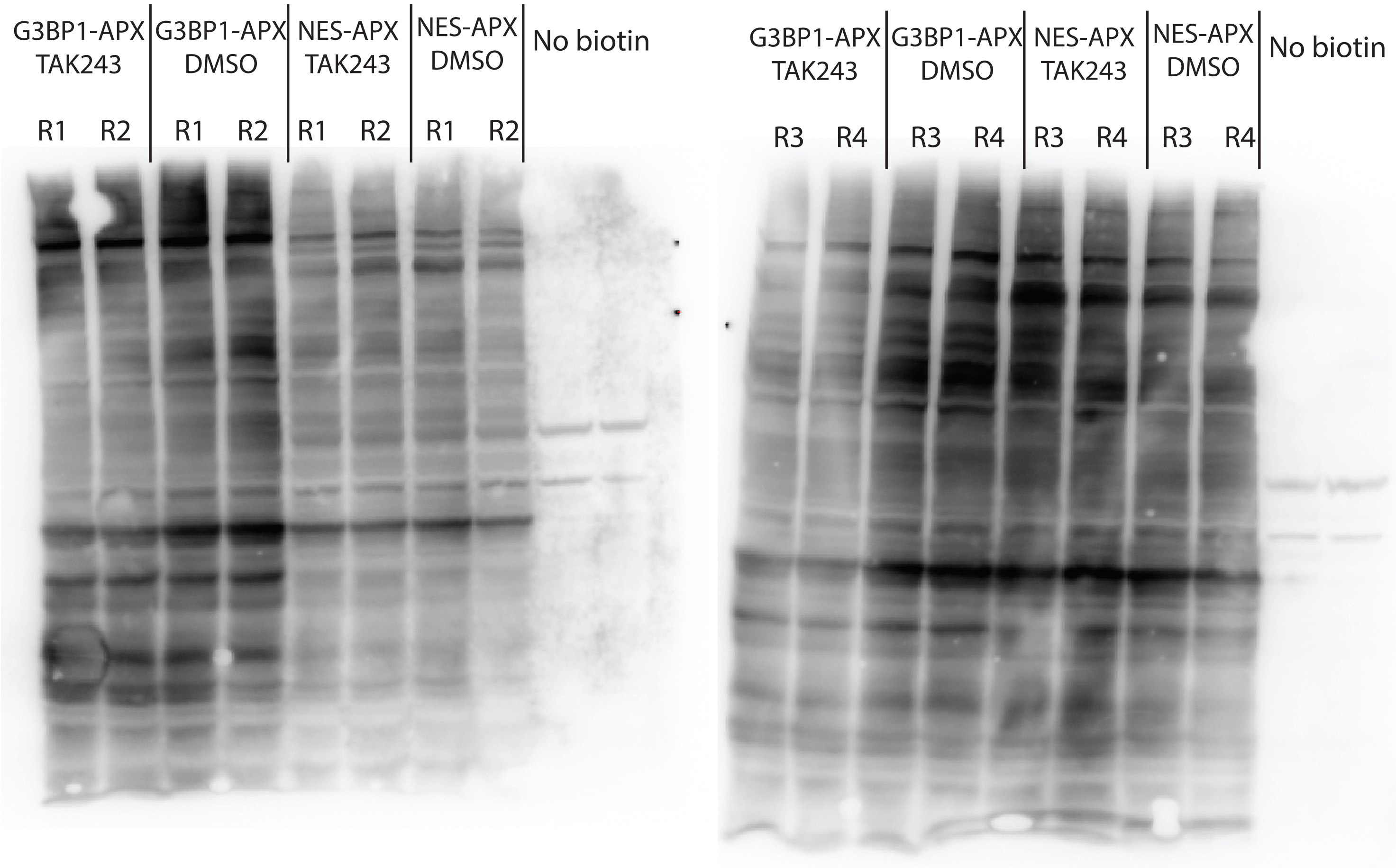
G3BP1-APX TAK243
G3BP1-APX DMSO
NES-APX TAK243
NES-APX DMSO
No biotin
R1 R2 R1 R2 R1 R2 R1 R2
G3BP1-APX TAK243
G3BP1-APX DMSO
NES-APX TAK243
NES-APX DMSO
No biotin
R3 R4 R3 R4 R3 R4 R3 R4

B

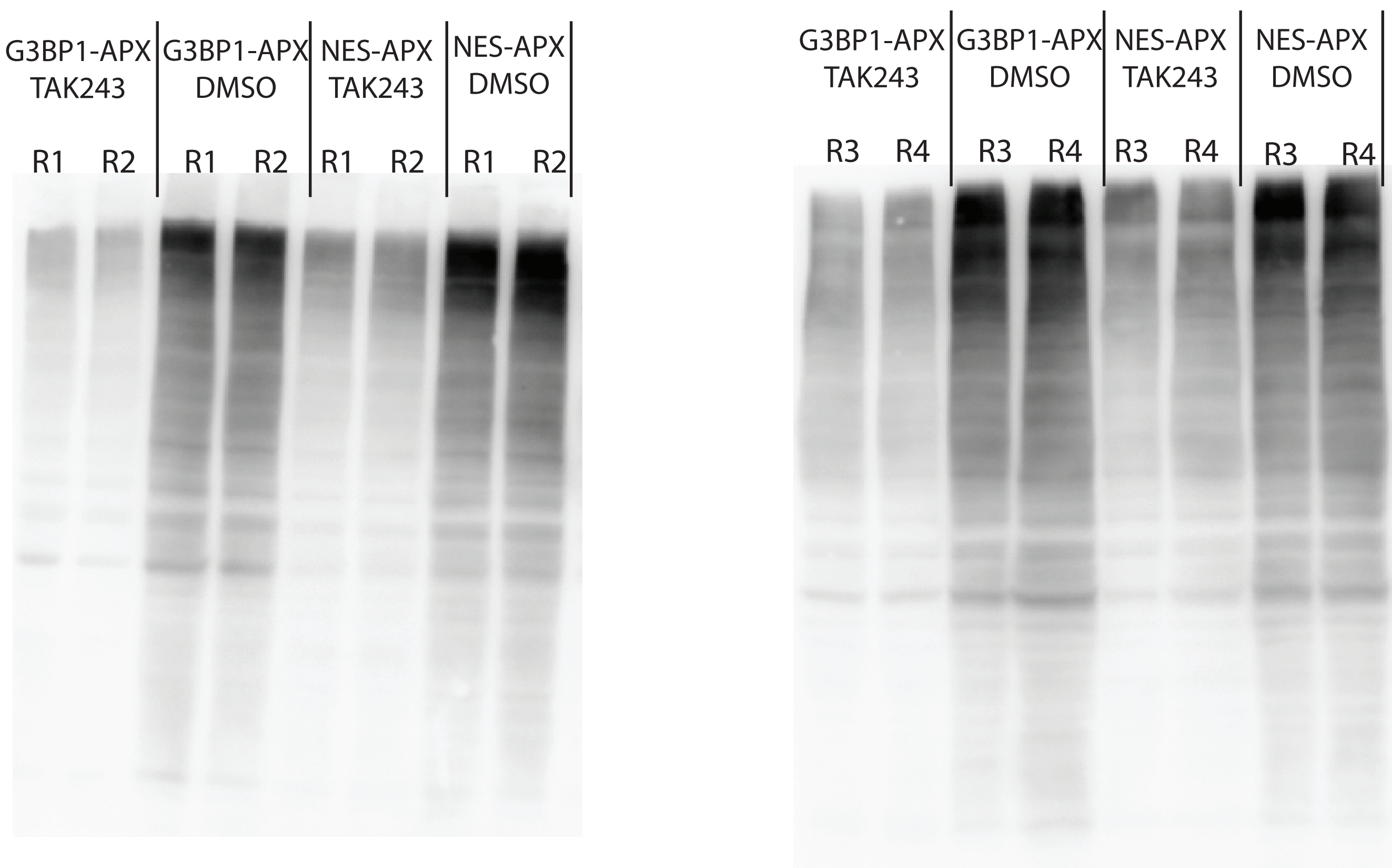
G3BP1-APX TAK243
G3BP1-APX DMSO
NES-APX TAK243
NES-APX DMSO
R1 R2 R1 R2 R1 R2 R1 R2
G3BP1-APX TAK243
G3BP1-APX DMSO
NES-APX TAK243
NES-APX DMSO
R3 R4 R3 R4 R3 R4 R3 R4

Appendix Figure S3. APEX samples. **A.** Anti-Biotin Western blot of total cell lysates for samples used in APEX proteomics experiment. **B.** Anti-ubiquitin (FK2) Western blot for samples used in APEX proteomics experiment.

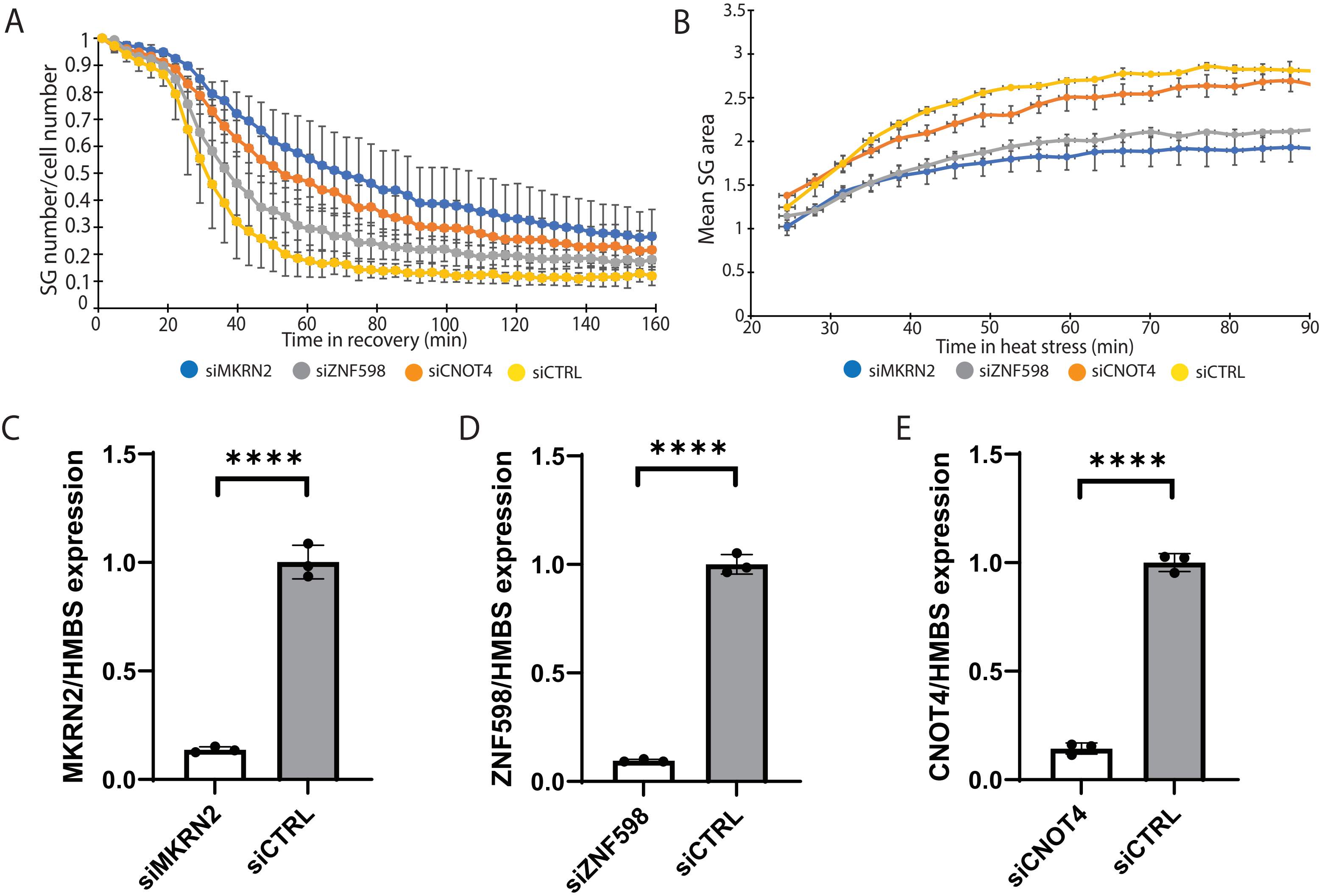
A
SG number/cell number
Time in recovery (min)
siMKRN2
siZNF598
siCNOT4
siCTRL
B
Mean SG area
Time in heat stress (min)
siMKRN2
siZNF598
siCNOT4
siCTRL
C
MKRN2/HMBS expression
****
siMKRN2
siCTRL
D
ZNF598/HMBS expression
****
siZNF598
siCTRL
E
CNOT4/HMBS expression
****
siCNOT4
siCTRL

Appendix Figure S4. Live imaging with MKRN2, ZNF598 and CNOT4. **A.** SG number/cell number during stress recovery (normalized to 90 min HS). Error bars = s.d. **B.** Average SG area over time in HS. Three biological repeats. Error bars = s.d. **C.** RT-qPCR expression of MKRN2 following MKRN2 KD. Three biological repeats. Two sample t test (two sided). Error bars = s.d.**D.** RT-qPCR expression of ZNF598 following ZNF598 KD. Three biological repeats. Two sample t test (two sided). Error bars = s.d. **E.** RT-qPCR expression of CNOT4 following CNOT4 KD. Two sample t test (two sided). Three biological repeats. Error bars = s.d. **** = <0.0001.

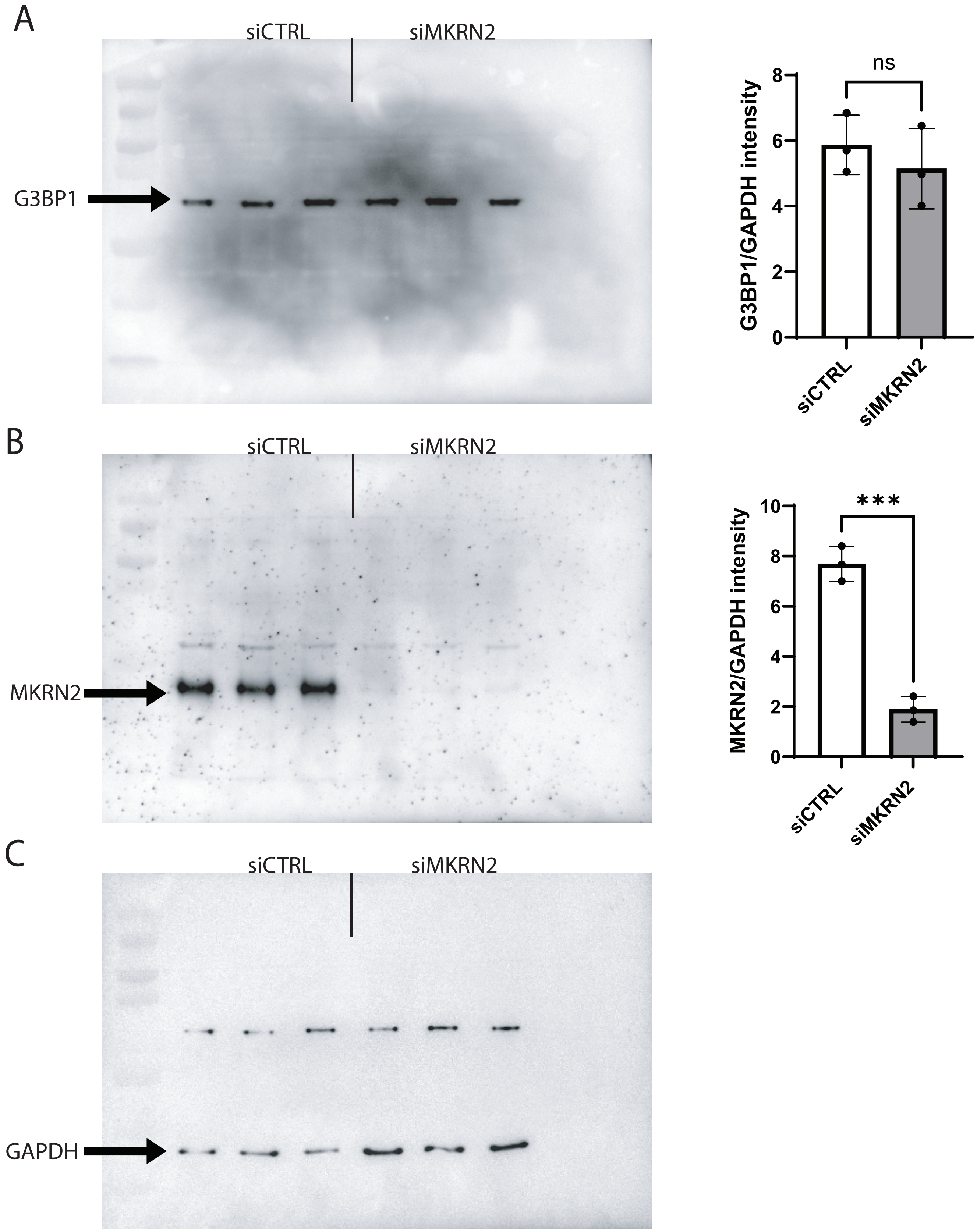
A
siCTRL
siMKRN2
G3BP1
ns
G3BP1/GAPDH intensity
8
6
4
2
0
siCTRL
siMKRN2
B
siCTRL
siMKRN2
MKRN2
***
MKRN2/GAPDH intensity
10
8
6
4
2
0
siCTRL
siMKRN2
C
siCTRL
siMKRN2
GAPDH

Appendix Figure S5: Expression of G3BP1 following MKRN2 KD. **A.** Western blot for G3BP1 following MKRN2 KD with quantification. Three biological repeats. Two sample t test (two sided). Error bars = s.d. **B.** Western blot for MKRN2 following MKRN2 KD with quantification. Three biological repeats. Two sample t test (two sided). **C.** Western blot for GAPDH as loading control. Error bars = s.d. *** = <0.001.

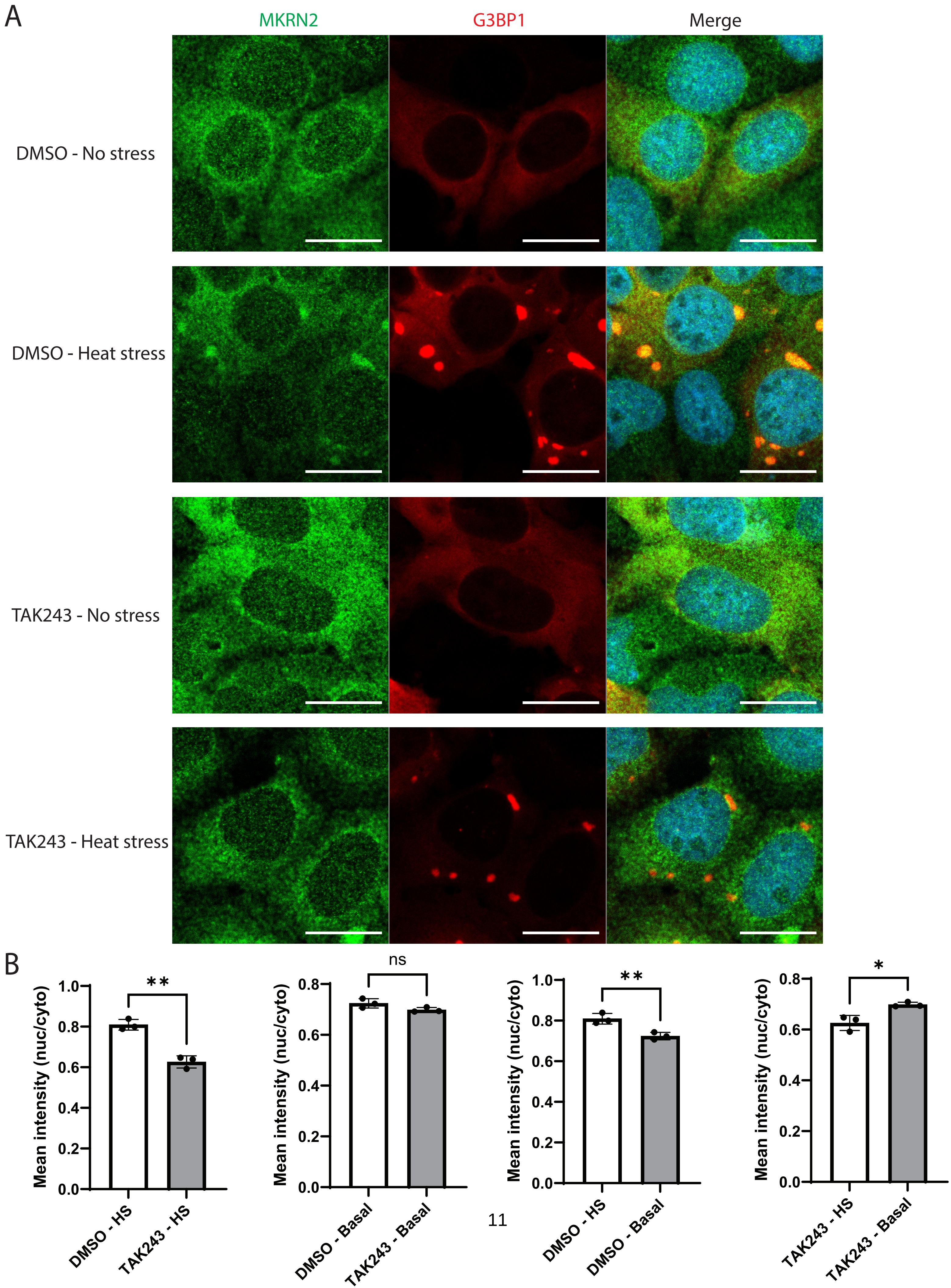
A
MKRN2
G3BP1
Merge
DMSO - No stress
DMSO - Heat stress
TAK243 - No stress
TAK243 - Heat stress
B
Mean intensity (nuc/cyto)
**
DMSO - HS
TAK243 - HS
ns
DMSO - Basal
TAK243 - Basal
**
DMSO - HS
DMSO - Basal
*
TAK243 - HS
TAK243 - Basal

Appendix Figure S6: Subcellular localization of endogenous MKRN2. **A.** Representative images of G3BP1-mCherry U2OS cells stained for endogenous MKRN2 and treated with the indicated treatment conditions. Scale bar = 20um **B.** Quantification of nuclear vs cytoplasmic expression level of endogenous MKRN2 in each condition. The ratio of the mean intensity in the nucleus and the mean intensity in the cytoplasm (nuc/cyto) is shown. Three biological repeats. Error bars = s.d. Two sample t test (two sided). * = <0.05; **<0.01.

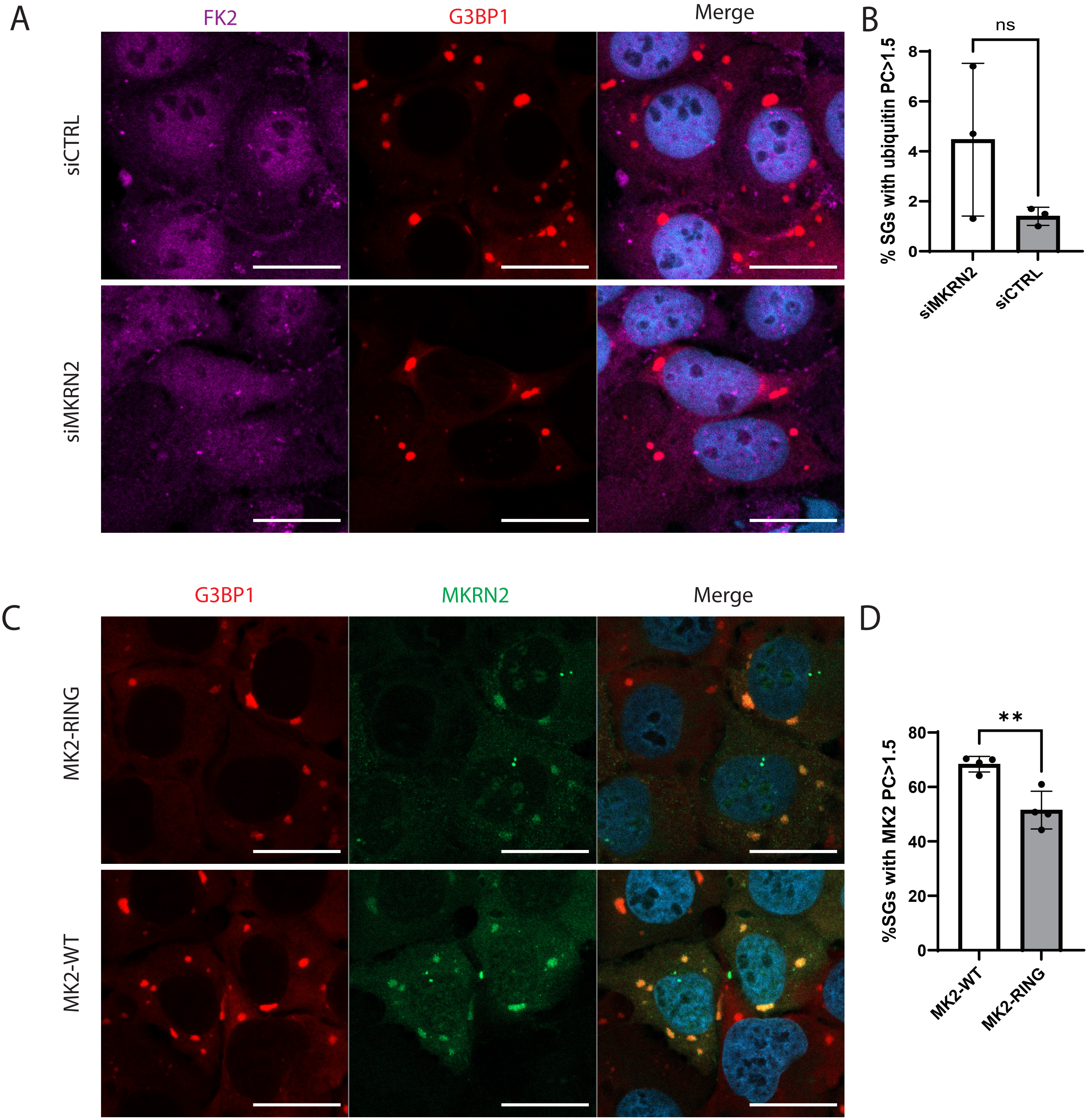
A
FK2
G3BP1
Merge
siCTRL
siMKRN2
B
ns
% SGs with ubiquitin PC>1.5
8
6
4
2
0
siMKRN2
siCTRL
C
G3BP1
MKRN2
Merge
MK2-RING
MK2-WT
D
**
%SGs with MK2 PC>1.5
80
60
40
20
0
MK2-WT
MK2-RING

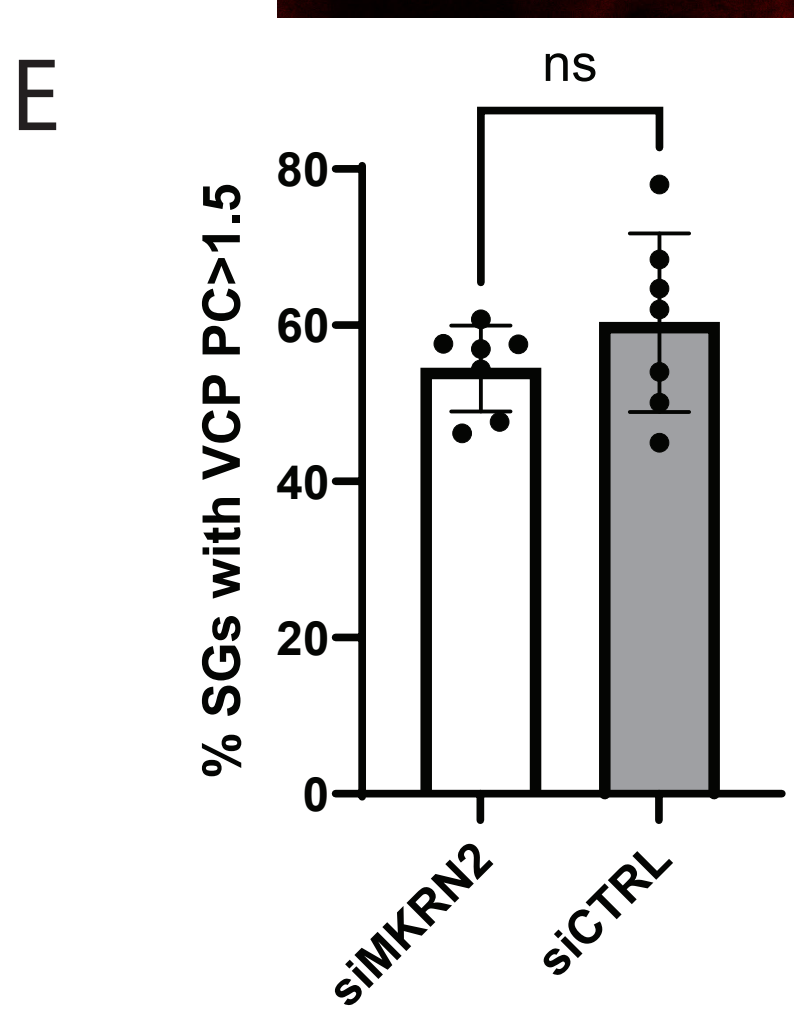
E
ns
% SGs with VCP PC>1.5
80
60
40
20
0
siMKRN2
siCTRL

Appendix Figure S7: Ubiquitin, VCP and MKRN2-RING partitioning to SGs. **A.** Representative images of G3BP1-mCherry cells following MKRN2 KD. Scale bar = 20um. **B.** Quantification of the partition coefficient of ubiquitin (FK2) in SGs following MKRN2 KD. The proportion of SGs with PC>1.5 is shown. Three biological repeats. Two sample t test (two sided). Error bars = s.d. **C.** Representative images of G3BP1-mCherry cells expressing GFP fusion proteins of either WT MKRN2 (MK2-WT) or a RING-mutant predicted to impair E3 ligase activity (MKRN2-RING). Scale bar = 20um. **D.** Quantification of the partition coefficient of MKRN2-GFP in SGs of cells expressing either MK2-WT or MK2-RING. The proportion of SGs with PC>1.5 is shown. Four biological repeats. Two sample t test (two sided). Error bars = s.d. **E.** Quantification of the partition coefficient of VCP in SGs following MKRN2 KD. The proportion of SGs with PC>1.5 is shown. Seven biological repeats. Error bars = s.d. Two sample t test. * = <0.01.

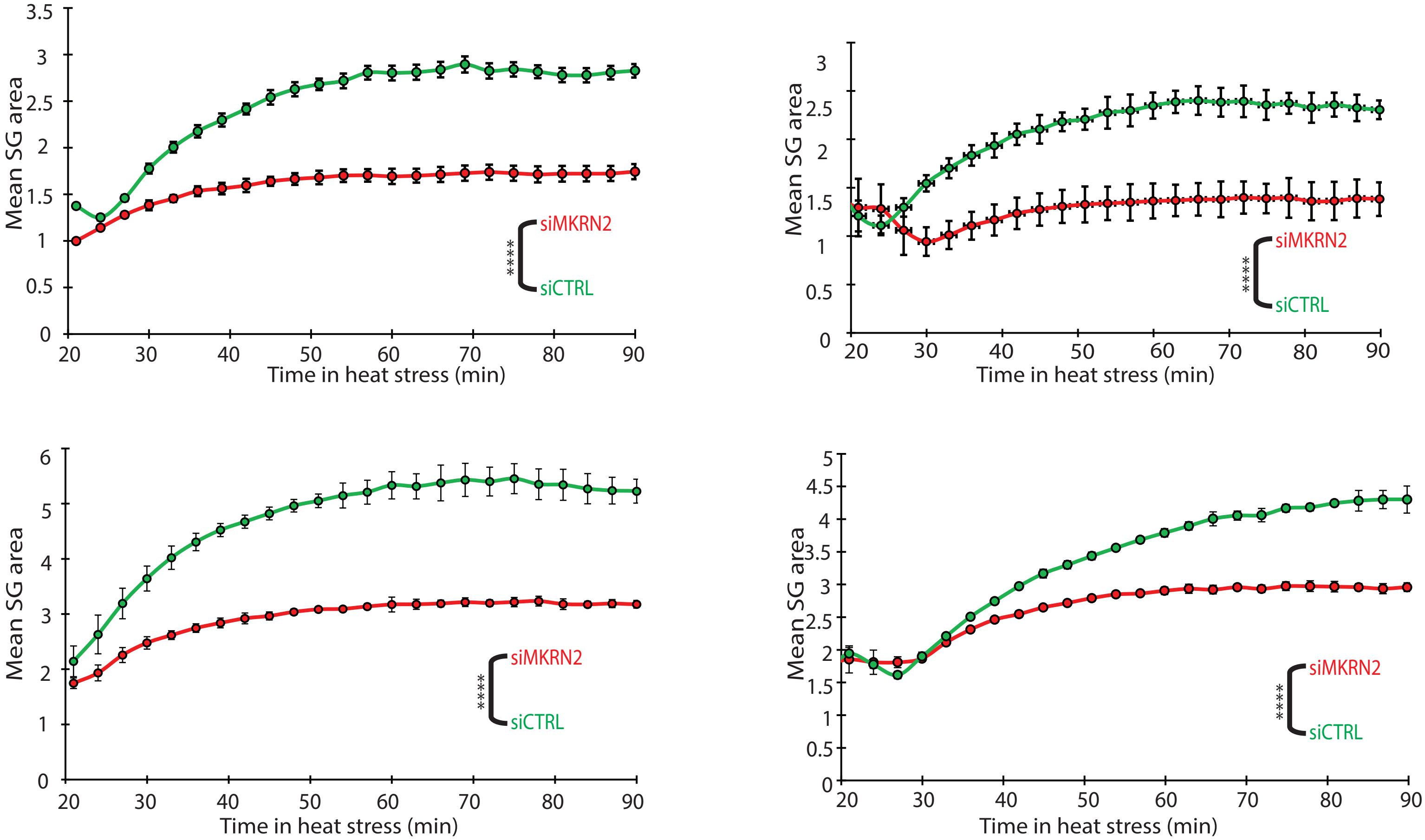
Mean SG area
Time in heat stress (min)
siMKRN2
siCTRL
****
Mean SG area
Time in heat stress (min)
siMKRN2
siCTRL
****
Mean SG area
Time in heat stress (min)
siMKRN2
siCTRL
****
Mean SG area
Time in heat stress (min)
siMKRN2
siCTRL
****

Appendix Figure S8: SG assembly live imaging repeats with MKRN2 KD. Live imaging quantification of SG area during HS at 43°C. 9-12 sites per condition. Four biological repeats shown (fifth repeat shown in Fig. 3C). Error bars = s.d. Two-way ANOVA with repeated measures. **** = <0.0001.

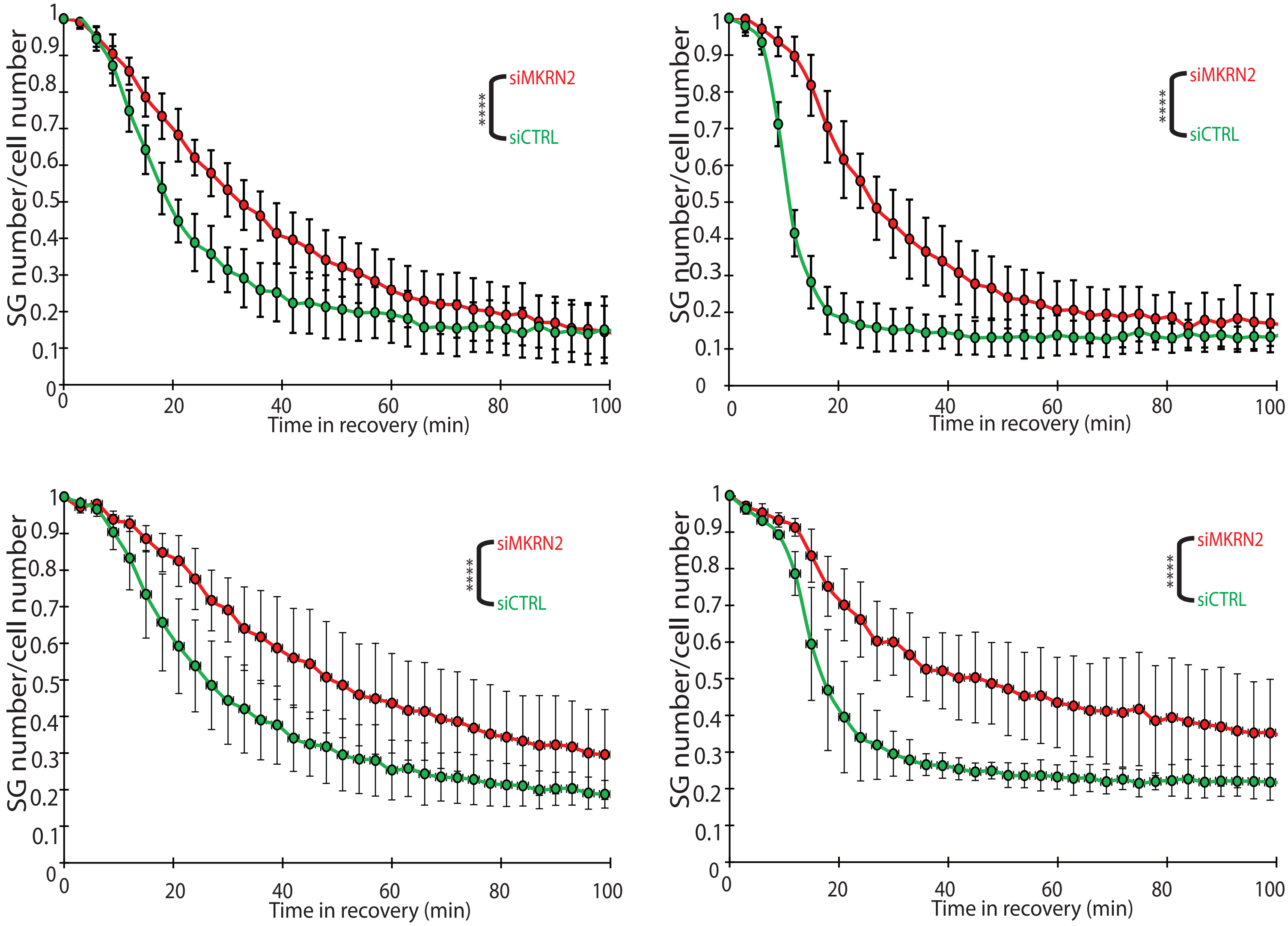
SG number/cell number
Time in recovery (min)
siMKRN2
siCTRL
****
SG number/cell number
Time in recovery (min)
siMKRN2
siCTRL
****
SG number/cell number
Time in recovery (min)
siMKRN2
siCTRL
****
SG number/cell number
Time in recovery (min)
siMKRN2
siCTRL
****

Appendix Figure S9. SG disassembly live imaging repeats with MKRN2 KD. Live imaging quantification of SG number/cell number during recovery at 37°C. Data were internally normalized to 90 min of HS. 9-12 sites per condition. Four biological repeats shown (fifth repeat shown in Fig. 4B). Error bars = s.d. Two-way ANOVA with repeated measures. **** = <0.0001.

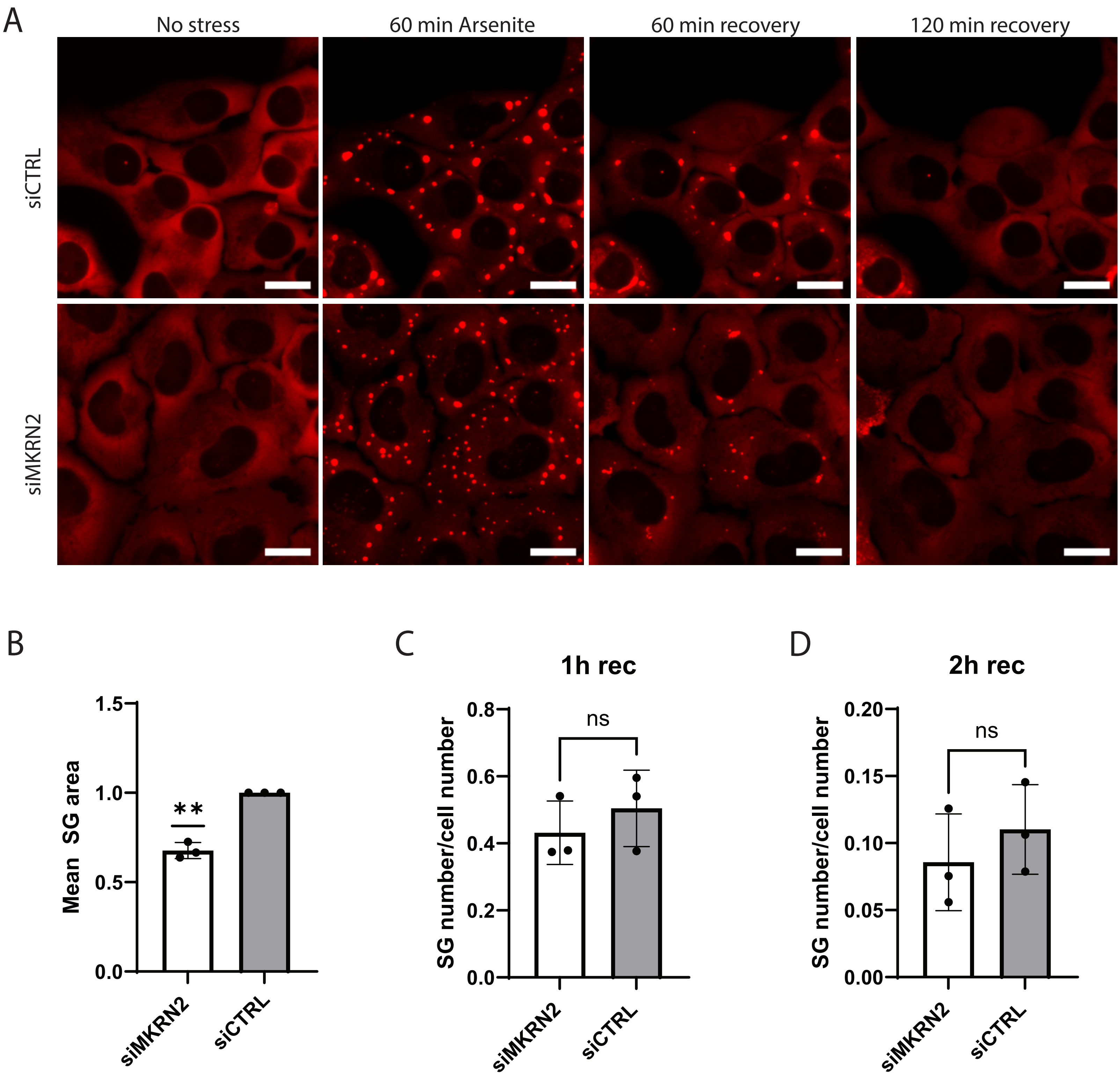
A
No stress
60 min Arsenite
60 min recovery
120 min recovery
siCTRL
siMKRN2
B
Mean SG area
1.5
1.0
0.5
0.0
**
siMKRN2
siCTRL
C
1h rec
SG number/cell number
0.8
0.6
0.4
0.2
0.0
ns
siMKRN2
siCTRL
D
2h rec
SG number/cell number
0.20
0.15
0.10
0.05
0.00
ns
siMKRN2
siCTRL

Appendix Figure S10. Live imaging with sodium arsenite **A.** Representative images of G3BP1-mCherry cells either without stress, with oxidative stress (sodium arsenite; 250uM; 60 min), and during stress recovery after removal of stress. Scale bar = 20um. **B.** Quantification of average SG area following 60 min of sodium arsenite stress. Data were internally normalized to siCTRL. The average of three biological repeats is shown. One sample t-test. Error bars = s.d. **C.** Quantification of SG number/cell number at 60 min recovery for siMKRN2 vs siCTRL. Average of three biological repeats shown. Data were normalized to 60 min of arsenite stress to track disassembly progress. Error bars = s.d. Two sample t test (two sided). **D.** Quantification of SG number/cell number at 120 min recovery for siMKRN2 vs siCTRL. Average of three biological repeats shown. Data were normalized to 60 min of arsenite stress to track disassembly progress. Error bars = s.d. Two sample t test (two sided).* = <0.01.

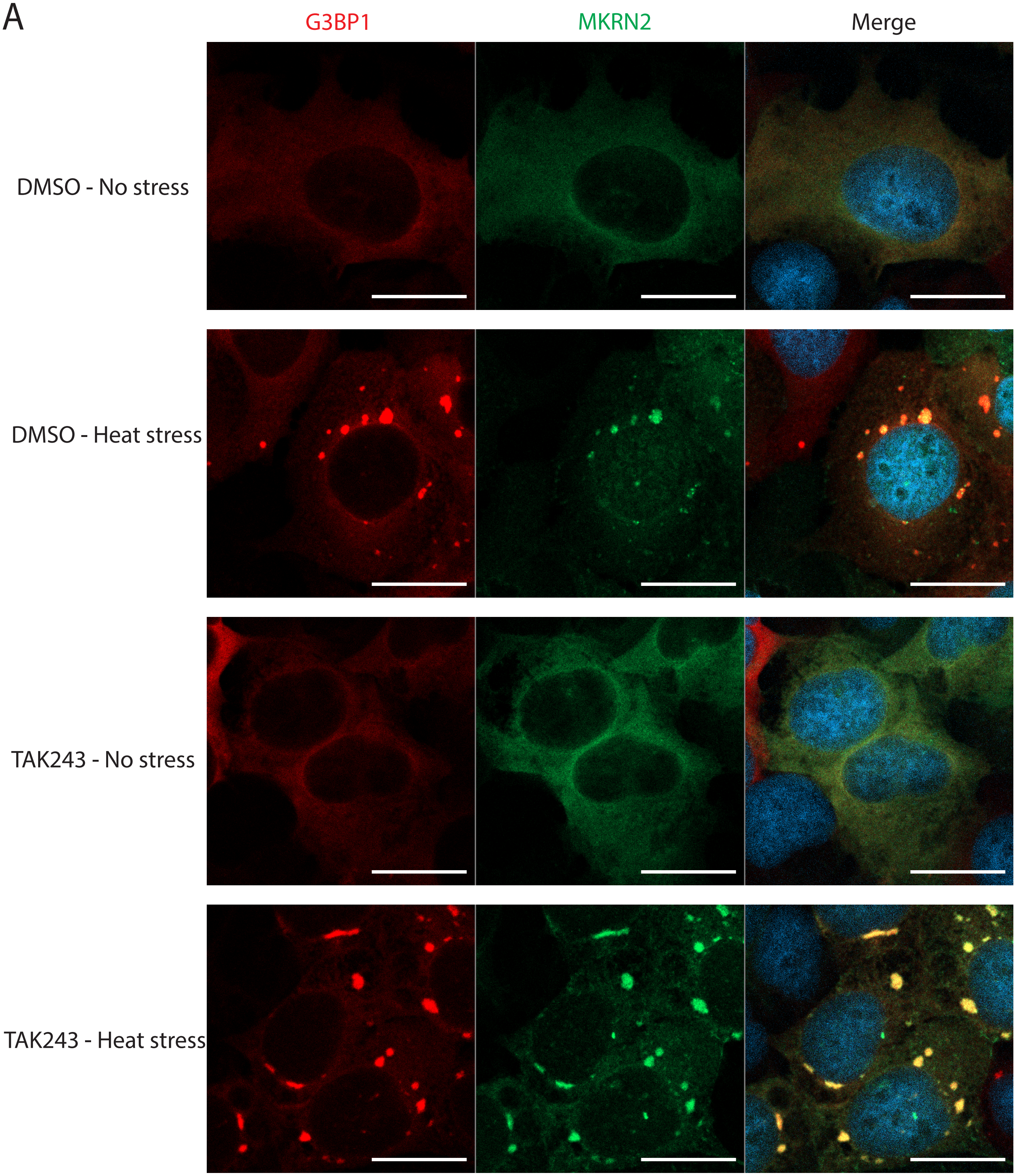
A
G3BP1
MKRN2
Merge
DMSO - No stress
DMSO - Heat stress
TAK243 - No stress
TAK243 - Heat stress

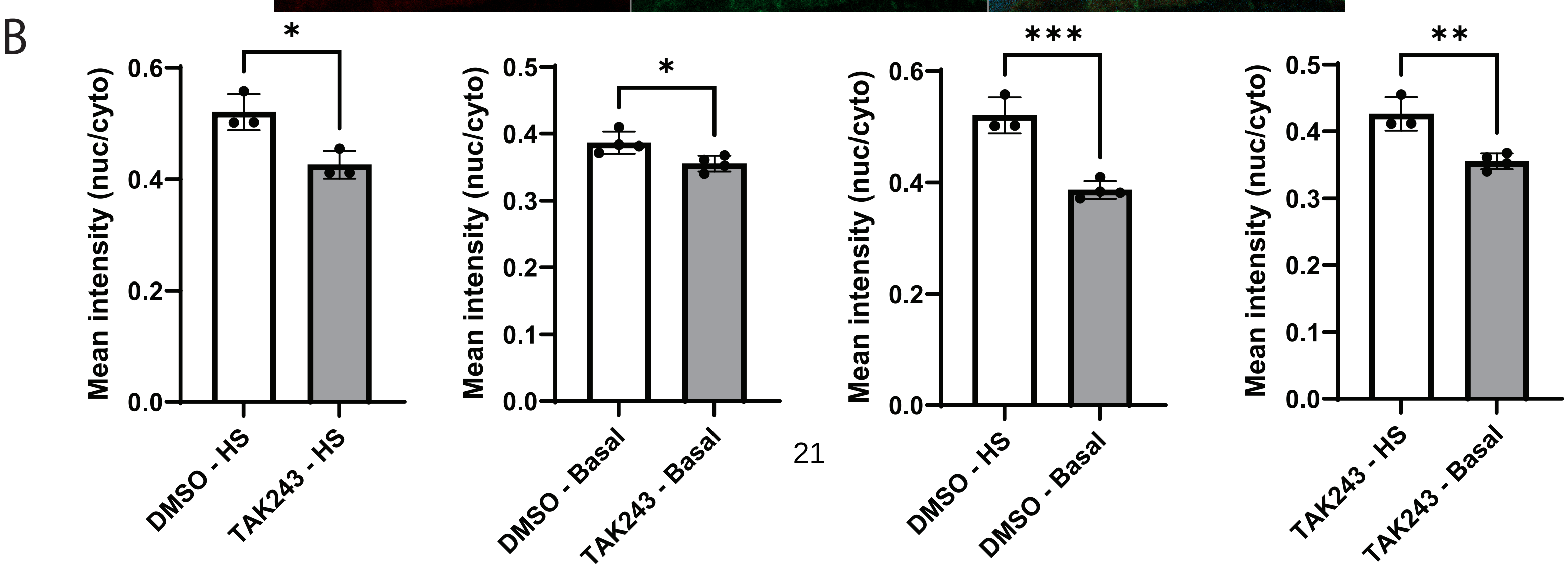
B
Mean intensity (nuc/cyto)
0.0
0.2
0.4
0.6
*
DMSO - HS
TAK243 - HS
Mean intensity (nuc/cyto)
0.0
0.1
0.2
0.3
0.4
0.5
*
DMSO - Basal
TAK243 - Basal
Mean intensity (nuc/cyto)
0.0
0.2
0.4
0.6
***
DMSO - HS
DMSO - Basal
Mean intensity (nuc/cyto)
0.0
0.1
0.2
0.3
0.4
0.5
**
TAK243 - HS
TAK243 - Basal

Appendix Figure S11: Subcellular localization of overexpressed MKRN2-GFP **A.** Representative images of G3BP1-mCherry U2OS cells expressing MKRN2-GFP and treated with the indicated treatment conditions. Scale bar = 20um. **B.** Quantification of nuclear vs cytoplasmic expression level of MKRN2-GFP in each condition. The ratio of the mean intensity in the nucleus and the mean intensity in the cytoplasm (nuc/cyto) is shown. Three biological repeats. Error bars = s.d. Two sample t test (two sided). * = <0.001.
