# Supplementary material for: The E3 ligase MKRN2 prevents DRiP accumulation in stress granules and maintains granulostasis
Source: EMBO Rep. 2026 Jun 15;27(15):4238–51. doi: 10.1038/s44319-026-00832-2 (PMC13458048; doi:10.1038/s44319-026-00832-2)
Supplement: Supplementary file 2 [file 44319_2026_832_MOESM3_ESM.pdf]

EMBO reports

The E3 ligase MKRN2 prevents DRiP accumulation in stress granules and maintains granulostasis

Emmanuel Amzallag, Yehuda Danino, Valentina Secco, Serena Carra, and Eran Hornstein

Corresponding author(s): Eran Hornstein (eran.hornstein@weizmann.ac.il)

Review Timeline:

Transfer Date:	19th Jan 26
Editorial Decision:	28th Jan 26
Revision Received:	7th Apr 26
Editorial Decision:	12th May 26
Revision Received:	14th May 26
Accepted:	22nd May 26

Editor: Martina Rembold

Transaction Report: This manuscript was transferred to EMBO Reports following peer review at Review Commons.

Review COMMONS

(Note: With the exception of the correction of typographical or spelling errors that could be a source of ambiguity, letters and reports are not edited. Depending on transfer agreements, referee reports obtained elsewhere may or may not be included in this compilation. Referee reports are anonymous unless the Referee chooses to sign their reports.)

Review #1

1. Evidence, reproducibility and clarity:

Evidence, reproducibility and clarity (Required)

Summary:

In this study, the authors used proximity proteomics in U2OS cells to identify several E3 ubiquitin ligases recruited to stress granules (SGs), and they focused on MKRN2 as a novel regulator. They show that MKRN2 localization to SGs requires active ubiquitination via UBA1. Functional experiments demonstrated that MKRN2 knockdown increases the number of SG condensates, reduces their size, slightly raises SG liquidity during assembly, and slows disassembly after heat shock. Overexpression of MKRN2-GFP combined with confocal imaging revealed co-localization of MKRN2 and ubiquitin in SGs. By perturbing ubiquitination (using a UBA1 inhibitor) and inducing defective ribosomal products (DRiPs) with O-propargyl puromycin, they found that both ubiquitination inhibition and MKRN2 depletion lead to increased accumulation of DRiPs in SGs. The authors conclude that MKRN2 supports granulostasis, the maintenance of SG homeostasis , through its ubiquitin ligase activity, preventing pathological DRiP accumulation within SGs.

Major comments:

- Are the key conclusions convincing?

The key conclusions are partially convincing. The data supporting the role of ubiquitination and MKRN2 in regulating SG condensate dynamics are coherent, well controlled, and consistent with previous literature, making this part of the study solid and credible. However, the conclusions regarding the ubiquitin-dependent recruitment of MKRN2 to SGs, its relationship with UBA1 activity, the functional impact of the MKRN2 knockdown for DRiP accumulation are less thoroughly supported. These aspects would benefit from additional mechanistic evidence, validation in complementary model systems, or the use of alternative methodological approaches to strengthen the causal connections drawn by the authors.
- Should the authors qualify some of their claims as preliminary or speculative, or remove them altogether?
The authors should qualify some of their claims as preliminary.

1) MKRN2 recruitment to SGs (ubiquitin-dependent): The proteomics and IF data are a

reasonable starting point, but they do not yet establish that MKRN2 is recruited from its physiological localization to SGs in a ubiquitin-dependent manner. To avoid overstating this point the authors should qualify the claim and/or provide additional controls: show baseline localization of endogenous MKRN2 under non-stress conditions (which is reported in literature to be nuclear and cytoplasmatic), include quantification of nuclear/cytoplasmic distribution, and demonstrate a shift into bona fide SG compartments after heat shock. Moreover, co-localization of overexpressed GFP-MKRN2 with poly-Ub (FK2) should be compared to a non-stress control and to UBA1-inhibition conditions to support claims of stress- and ubiquitination-dependent recruitment.

2) Use and interpretation of UBA1 inhibition: UBA1 inhibition effectively blocks ubiquitination globally, but it is non-selective. The manuscript should explicitly acknowledge this limitation when interpreting results from both proteomics and functional assays. Proteomics hits identified under UBA1 inhibition should be discussed as UBA1-dependent associations rather than as evidence for specific E3 ligase recruitment. The authors should consider orthogonal approaches before concluding specificity.

3) DRiP accumulation and imaging quality: The evidence presented in Figure 5 is sufficient to substantiate the claim that DRiPs accumulate in SGs upon ubiquitination inhibition or MKRN2 depletion but to show that the event of the SGs localization and their clearance from SGs during stress is promoted by MKRN3 ubiquitin ligase activity more experiments would be needed.
- Would additional experiments be essential to support the claims of the paper? Request additional experiments only where necessary for the paper as it is, and do not ask authors to open new lines of experimentation.
Yes, a few targeted experiments would strengthen the conclusions without requiring the authors to open new lines of investigation.

1) Baseline localization of MKRN2: It would be important to show the baseline localization of endogenous and over-expressed MKRN2 (nuclear and cytoplasmic) under non-stress conditions and prior to ubiquitination inhibition. This would provide a reference to quantify redistribution into SGs and demonstrate recruitment in response to heat stress or ubiquitination-dependent mechanisms.

2) Specificity of MKRN2 ubiquitin ligase activity: to address the non-specific effects of UBA1 inhibition and validate that observed phenotypes depend on MKRN2's ligase activity, the authors could employ a catalytically inactive MKRN2 mutant in rescue experiments. Comparing wild-type and catalytic-dead MKRN2 in the knockdown background would

clarify the causal role of MKRN2 activity in SG dynamics and DRiP clearance.

3) Ubiquitination linkage and SG marker levels: While the specific ubiquitin linkage type remains unknown, examining whether MKRN2 knockdown or overexpression affects total levels of key SG marker proteins would be informative. This could be done via Western blotting of SG markers along with ubiquitin staining, to assess whether MKRN2 influences protein stability or turnover through degradative or non-degradative ubiquitination. Such data would strengthen the mechanistic interpretation while remaining within the current study's scope.

- Are the suggested experiments realistic in terms of time and resources? It would help if you could add an estimated cost and time investment for substantial experiments.

The experiments suggested in points 1 and 3 are realistic and should not require substantial additional resources beyond those already used in the study.

- Point 1 (baseline localization of MKRN2): This involves adding two control conditions (no stress and no ubiquitination inhibition) for microscopy imaging. The setup is essentially the same as in the current experiments, with time requirements mainly dependent on cell culture growth and imaging. Overall, this could be completed within a few weeks.
- Point 3 (SG marker levels and ubiquitination): This entails repeating the existing experiment and adding a Western blot for SG markers and ubiquitin. The lab should already have the necessary antibodies, and the experiment could reasonably be performed within a couple of weeks.
- Point 2 (catalytically inactive MKRN2 mutant and rescue experiments): This is likely more time-consuming. Designing an effective catalytic-dead mutant depends on structural knowledge of MKRN2 and may require additional validation to confirm loss of catalytic activity. If this expertise is not already present in the lab, it could significantly extend the timeline. Therefore, this experiment should be considered only if similarly recommended by other reviewers, as it represents a higher resource and time investment.

Overall, points 1 and 3 are highly feasible, while point 2 is more substantial and may require careful planning.

- Are the data and the methods presented in such a way that they can be reproduced?

Yes. The methodologies used in this study to analyze SG dynamics and DRiP accumulation are well-established in the field and should be reproducible, particularly by researchers experienced in stress granule biology. Techniques such as SG assembly and disassembly assays, use of G3BP1 markers, and UBA1 inhibition are standard and clearly described.
The data are generally presented in a reproducible manner; however, as noted above, some results would benefit from additional controls or complementary experiments to fully

support specific conclusions.
- Are the experiments adequately replicated and statistical analysis adequate?

Overall, the experiments in the manuscript appear to be adequately replicated, with most assays repeated between three and five times, as indicated in the supplementary materials. The statistical analyses used are appropriate and correctly applied to the datasets presented. However, for Figure 5 the number of experimental replicates is not reported. This should be clarified, and if the experiment was not repeated sufficiently, additional biological replicates should be performed. Given that this figure provides central evidence supporting the conclusion that DRiP accumulation depends on ubiquitination-and partly on MKRN2's ubiquitin ligase activity-adequate replication is essential.

Minor comments:

- Specific experimental issues that are easily addressable.
- For the generation and the validation of MKRN2 knockdown in UOS2 cells data are not presented in the results or in the methods sections to demonstrate the effective knockdown of the protein of interest. This point is quite essential to demonstrate the validity of the system used
- In the supplementary figure 2 it would be useful to mention if the Western Blot represent the input (total cell lysates) before the APEX-pulldown or if it is the APEX-pulldown loaded for WB. There is no consistence in the difference of biotynilation between different replicates shown in the 2 blots. For example in R1 and R2 G3BP1-APX TAK243 the biotynilation is one if the strongest condition while on the left blot, in the same condition comparison samples R3 and R4 are less biotinilated compared to others. It would be useful to provide an explanation for that to avoid any confusion for the readers.
- In Figure 2D, endogenous MKRN2 localization to SGs appears reduced following UBA1 inhibition. However, it is not clear whether this reduction reflects a true relocalization or a decrease in total MKRN2 protein levels. To support the interpretation that UBA1 inhibition specifically affects MKRN2 recruitment to SGs rather than its overall expression, the authors should provide data showing total MKRN2 levels remain unchanged under UBA1 inhibition, for example via Western blot of total cell lysates.
- DRIPs accumulation is followed during assembly but in the introduction is highlighted the fact that ubiquitination events, other reported E3 ligases and in this study data on MKRN2 showed that they play a crucial role in the disassembly of SGs which is also related with cleareance of DRIPs. Authors could add tracking DRIPs accumulation during disassembly to be added to Figure 5. I am not sure about the timeline required for this but I am just adding as optional if could be addressed easily.

- The authors should clarify in the text why the cutoff used for the quantification in Figure 5D (PC > 3) differs from the cutoff used elsewhere in the paper (PC > 1.5). Providing a rationale for this choice will help the reader understand the methodological consistency and ensure that differences in thresholds do not confound interpretation of the results.
- For Figure 3G, the authors use over-expressed MKRN2-GFP to assess co-localization with ubiquitin in SGs. Given that a reliable antibody for endogenous MKRN2 is available and that a validated MKRN2 knockdown line exists as an appropriate control, this experiment would gain significantly in robustness and interpretability if co-localization were demonstrated using endogenous MKRN2. In the current over-expression system, MKRN2-GFP is also present in the nucleus, whereas the endogenous protein does not appear nuclear under the conditions shown. This discrepancy raises concerns about potential over-expression artifacts or mislocalization. Demonstrating co-localization using endogenous MKRN2 would avoid confounding effects associated with over-expression. If feasible, this would be a relatively straightforward experiment to implement, as it relies on tools (antibody and knockdown line) already described in the manuscript.
- Are prior studies referenced appropriately?

- From line 54 to line 67, the manuscript in total cites eight papers regarding the role of ubiquitination in SG disassembly. However, given the use of UBA1 inhibition in the initial MS-APEX experiment and the extensive prior literature on ubiquitination in SG assembly and disassembly under various stress conditions, the manuscript would benefit from citing additional relevant studies to provide more specifc examples. Expanding the references would provide stronger context, better connect the current findings to prior work, and emphasize the significance of the study in relation to established literature
- At line 59, it would be helpful to note that G3BP1 is ubiquitinated by TRIM21 through a Lys63-linked ubiquitin chain. This information provides important mechanistic context, suggesting that ubiquitination of SG proteins in these pathways is likely non-degradative and related to functional regulation of SG dynamics rather than protein turnover.
- When citing references 16 and 17, which report that the E3 ligases TRIM21 and HECT regulate SG formation, the authors should provide a plausible explanation for why these specific E3 ligases were not detected in their proteomics experiments. Differences could arise from the stress stimulus used, cell type, or experimental conditions. Similarly, since MKRN2 and other E3 ligases identified in this study have not been reported in previous works, discussing these methodological or biological differences would help prevent readers from questioning the credibility of the findings. It would also be valuable to clarify in the Conclusion that different types of stress may activate distinct ubiquitination pathways, highlighting context-dependent regulation of SG assembly and disassembly.
- Line 59-60: when referring to the HECT family of E3 ligases involved in ubiquitination and

SG disassembly, it would be more precise to report the specific E3 ligase identified in the cited studies rather than only the class of ligase. This would provide clearer mechanistic context and improve accuracy for readers.

- The specific statement on line 182 "SG E3 ligases that depend on UBA1 activity are RBULs" should be supported by reference.
- Are the text and figures clear and accurate?
- In Supplementary Figure 1, DMSO is shown in green and the treatment in red, whereas in the main figures (Figure 1B and 1F) the colours in the legend are inverted. To avoid confusion, the colour coding in figure legends should be consistent across all figures throughout the manuscript.
- At line 79, the manuscript states that "inhibition of ubiquitination delayed fluorescence recovery dynamics of G3BP1-mCherry, relative to HS-treated cells (Figure 1F, Supplementary Fig. 6A)." However, the data shown in Figure 1F appear to indicate the opposite effect: the TAK243-treated condition (green curve) shows a faster fluorescence recovery compared to the control (red curve). This discrepancy between the text and the figure should be corrected or clarified, as it may affect the interpretation of the role of ubiquitination in SG dynamics.
- Line 86: adjust a missing bracket
- There appears to be an error in the legend of Supplementary Figure 3: the legend states that the red condition (MKRN2) forms larger aggregates, but both the main Figure 3C of the confocal images and the text indicate that MKRN2 (red) forms smaller aggregates. Please correct the legend and any corresponding labels so they are consistent with the main figure and the text. The authors should also double-check that the figure panel order, color coding, and statistical annotations match the legend and the descriptions in the Results section to avoid reader confusion.
- At lines 129-130, the manuscript states that "FRAP analysis demonstrated that MKRN2 KD resulted in a slight increase in SG liquidity (Fig. 3F, Supplementary Fig. 6B)." However, the data shown in Figure 3F appear to indicate the opposite trend: the MKRN2 KD condition (red curve) exhibits a faster fluorescence recovery compared to the control (green curve). This discrepancy between the text and the figure should be corrected or clarified, as it directly affects the interpretation of MKRN2's role in SG disassembly. Ensuring consistency between the written description and the plotted FRAP data is essential for accurate interpretation.
- At lines 132-133, the manuscript states: "Then, to further test the impact of MKRN2 on SG dynamics, we overexpressed MKRN2-GFP and observed that it was recruited to SG (Fig. 3G)." This description should be corrected or clarified, as the over-expressed MKRN2-GFP also appears to localize to the nucleus.
- At lines 134-135, the manuscript states that the FK2 antibody detects "free ubiquitin." This

is incorrect. FK2 does not detect free ubiquitin; it recognizes only ubiquitin conjugates, including mono-ubiquitinated and poly-ubiquitinated proteins. The text should be corrected accordingly to avoid misinterpretation of the immunostaining data.
- Figure 5A suffers from poor resolution, and no scale bar is provided, which limits interpretability. Additionally, the ROI selected for the green channel (DRIPs) appears to capture unspecific background staining, while the most obvious DRIP spots are localized in the nucleus. The authors should clarify this in the text, improve the image quality if possible, and ensure that the ROI accurately represents DRIP accumulation - in SGs rather than background signal.

Do you have suggestions that would help the authors improve the presentation of their data and conclusions?

- In the first paragraph following the APEX proteomics results, the authors present validation data exclusively for MKRN2, justifying this early focus by stating that MKRN2 is the most SG-depleted E3 ligase. However, in the subsequent paragraph they introduce the RBULs and present knockdown data for MKRN2 along with two additional E3 ligases identified in the screen, before once again emphasizing that MKRN2 is the most SG-depleted ligase and therefore the main focus of the study. For clarity and logical flow, the manuscript would benefit from reordering the narrative. Specifically, the authors should first present the validation data for all three selected E3 ligases, and only then justify the decision to focus on MKRN2 for in-depth characterization. In addition to the extent of its SG depletion, the authors may also consider providing biologically relevant reasons for prioritizing MKRN2 (e.g., domain architecture, known roles in stress responses, or prior evidence of ubiquitination-related functions). Reorganizing this section would improve readability and better guide the reader through the rationale for the study's focus.
- At lines 137-138, the manuscript states: "Together these data indicate that MKRN2 regulates the assembly dynamics of SGs by promoting their coalescence during HS and can increase SG ubiquitin content." While Figure 3G shows some co-localization of MKRN2 with ubiquitin, immunofluorescence alone is insufficient to claim an increase in SG ubiquitin content. This conclusion should be supported by orthogonal experiments, such as Western blotting, in vitro ubiquitination assays, or immunoprecipitation of SG components. Including a control under no-stress conditions would also help demonstrate that ubiquitination increases specifically in response to stress. The second part of the statement should therefore be rephrased to avoid overinterpretation, for example:"...and may be associated with increased ubiquitination within SGs, as suggested by co-localization, pending further validation by complementary assays."
- At line 157, the statement: "Therefore, we conclude that MKRN2 ubiquitinates a subset of

DRiPs, avoiding their accumulation inside SGs" should be rephrased as a preliminary observation. While the data support a role for MKRN2 in SG disassembly and a reduction of DRIPs, direct ubiquitination of DRIPs by MKRN2 has not been demonstrated. A more cautious phrasing would better reflect the current evidence and avoid overinterpretation.

2. Significance:

Significance (Required)

General assessment: provide a summary of the strengths and limitations of the study. What are the strongest and most important aspects? What aspects of the study should be improved or could be developed?

- This study provides a valuable advancement in understanding the role of ubiquitination in stress granule (SG) dynamics and the clearance of SGs formed under heat stress. A major strength is the demonstration of how E3 ligases identified through proteomic screening, particularly MKRN2, influence SG assembly and disassembly in a ubiquitination- and heat stress-dependent manner. The combination of proteomics, imaging, and functional assays provides a coherent mechanistic framework linking ubiquitination to SG homeostasis. Limitations of the study include the exclusive use of a single model system (U2OS cells), which may limit generalizability. Additionally, some observations-such as MKRN2-dependent ubiquitination within SGs and changes in DRIP accumulation under different conditions-would benefit from orthogonal validation experiments (e.g., Western blotting, immunoprecipitation, or in vitro assays) to confirm and strengthen these findings. Addressing these points would enhance the robustness and broader applicability of the conclusions.

Advance: compare the study to the closest related results in the literature or highlight results reported for the first time to your knowledge; does the study extend the knowledge in the field and in which way? Describe the nature of the advance and the resulting insights (for example: conceptual, technical, clinical, mechanistic, functional,...).

- The closest related result in literature is - Yang, Cuiwei et al. "Stress granule homeostasis is modulated by TRIM21-mediated ubiquitination of G3BP1 and autophagy-dependent elimination of stress granules." Autophagy vol. 19,7 (2023): 1934-1951. doi:10.1080/15548627.2022.2164427 - demonstrating that TRIM21, an E3 ubiquitin ligase, catalyzes K63-linked ubiquitination of G3BP1, a core SG nucleator, under oxidative stress. This ubiquitination by TRIM21 inhibits SG formation, likely by altering G3BP1's propensity for phase separation. In contrast, the MKRN2 study identifies a different E3 (MKRN2) that

regulates SG dynamics under heat stress and appears to influence both assembly and disassembly. This expands the role of ubiquitin ligases in SG regulation beyond those previously studied (like TRIM21).
- Gwon and colleagues (Gwon Y, Maxwell BA, Kolaitis RM, Zhang P, Kim HJ, Taylor JP. Ubiquitination of G3BP1 mediates stress granule disassembly in a context-specific manner. Science. 2021;372(6549):eabf6548. doi:10.1126/science.abf6548) have shown that K63-linked ubiquitination of G3BP1 is required for SG disassembly after heat stress. This ubiquitinated G3BP1 recruits the segregase VCP/p97, which helps extract G3BP1 from SGs for disassembly. The MKRN2 paper builds on this by linking UBA1-dependent ubiquitination and MKRN2's activity to SG disassembly. Specifically, they show MKRN2 knockdown affects disassembly, and suggest MKRN2 helps prevent accumulation of defective ribosomal products (DRiPs) in SGs, adding a new layer to the ubiquitin-VCP model.
- Ubiquitination's impact is highly stress- and context-dependent (different chain types, ubiquitin linkages, and recruitment of E3s). The MKRN2 work conceptually strengthens this idea: by showing that MKRN2's engagement with SGs depends on active ubiquitination via UBA1, and by demonstrating functional consequences (SG dynamics + DRIP accumulation), the study highlights how cellular context (e.g., heat stress) can recruit specific ubiquitin ligases to SGs and modulate their behavior.
- There is a gap in the literature: very few (if any) studies explicitly combine the biology of DRIPs, stress granules, and E3 ligase mediated ubiquitination, especially in mammalian cells. There are relevant works about DRIP biology in stress granules, but those studies focus on chaperone-based quality control, not ubiquitin ligase-mediated ubiquitination of DRIPs. This study seems to be one of the first to make that connection in mammalian (or human-like) SG biology. A work on the plant DRIP-E3 ligase TaSAP5 (Zhang N, Yin Y, Liu X, et al. The E3 Ligase TaSAP5 Alters Drought Stress Responses by Promoting the Degradation of DRIP Proteins. Plant Physiol. 2017;175(4):1878-1892. doi:10.1104/pp.17.01319) shows that DRIPs can be directly ubiquitinated by E3s in other biological systems - which supports the plausibility of the MKRN2 mechanism, but it's not the same context.
- A very recent review (Yuan, Lin et al. "Stress granules: emerging players in neurodegenerative diseases." Translational neurodegeneration vol. 14,1 22. 12 May. 2025, doi:10.1186/s40035-025-00482-9) summarizes and reinforces the relationship among SGs and the pathogenesis of different neurodegenerative diseases (NDDs). By identifying MKRN2 as a new ubiquitin regulator in SGs, the current study could have relevance for neurodegeneration and proteotoxic diseases, providing a new candidate to explore in disease models.

Audience: describe the type of audience ("specialized", "broad", "basic research",

"translational/clinical", etc...) that will be interested or influenced by this research; how will this research be used by others; will it be of interest beyond the specific field?

The audience for this paper is primarily specialized, including researchers in stress granule biology, ubiquitin signaling, protein quality control, ribosome biology, and cellular stress responses. The findings will also be of interest to scientists working on granulostasis, nascent protein surveillance, and proteostasis mechanisms. Beyond these specific fields, the study provides preliminary evidence linking ubiquitination to DRIP handling and SG dynamics, which may stimulate new research directions and collaborative efforts across complementary areas of cell biology and molecular biology.

Please define your field of expertise with a few keywords to help the authors contextualize your point of view. Indicate if there are any parts of the paper that you do not have sufficient expertise to evaluate.

I work in ubiquitin biology, focusing on ubiquitination signaling in physiological and disease contexts, with particular expertise in the identification of E3 ligases and their substrates across different cellular systems and in vivo models. I have less expertise in stress granule dynamics and DRiP biology, so my evaluation of those aspects is more limited and relies on interpretation of the data presented in the manuscript.

3. How much time do you estimate the authors will need to complete the suggested revisions:

Estimated time to Complete Revisions (Required)

(Decision Recommendation)

Between 3 and 6 months

4. *Review Commons* values the work of reviewers and encourages them to get credit for their work. Select 'Yes' below to register your reviewing activity at Web of Science Reviewer Recognition Service (formerly Publons); note that the content of your review will not be visible on Web of Science.

Web of Science Reviewer Recognition

Yes

Review #2

1. Evidence, reproducibility and clarity:

Evidence, reproducibility and clarity (Required)

This study identifies the E3 ubiquitin ligase Makorin 2 (MKRN2) as a novel regulator of stress granule (SG) dynamics and proteostasis. Using APEX proximity proteomics, the authors demonstrate that inhibition of the ubiquitin-activating enzyme UBA1 with TAK243 alters the SG proteome, leading to depletion of several E3 ligases, chaperones, and VCP cofactors. Detailed characterization of MKRN2 reveals that it localizes to SGs in a ubiquitination-dependent manner and is required for proper SG assembly, coalescence, and disassembly. Functionally, MKRN2 prevents the accumulation of defective ribosomal products (DRiPs) within SGs, thereby maintaining granulostasis. The study provides compelling evidence that ubiquitination, mediated specifically by MKRN2, plays a critical role in surveilling stress-damaged proteins within SGs and maintaining their dynamic liquid-like properties.

Major issues:

1. Figures 1-2: Temporal dynamics of ubiquitination in SGs. The APEX proteomics was performed at a single timepoint (90 min heat stress), yet the live imaging data show that SG dynamics and TAK243 effects vary considerably over time:
- The peak or SG nucleation was actually at 10-30 min (Figure 1B).
- TAK243 treatment causes earlier SG nucleation (Figure 1B) but delayed disassembly (Figure 1A-B, D).

A temporal proteomic analysis at multiple timepoints (e.g., 30 min, 60 min, 90 min of heat stress, and during recovery) would reveal whether MKRN2 and other ubiquitination-dependent proteins are recruited to SGs dynamically during the stress response. It would also delineate whether different E3 ligases predominate at different stages of the SG lifecycle.
While such experiments may be beyond the scope of the current study, the authors should at minimum discuss this limitation and acknowledge that the single-timepoint analysis may miss dynamic changes in SG composition.

2. Figures 2D-E, 3G: MKRN2 localization mechanism requires clarification. The authors demonstrate that MKRN2 localization to SGs is dependent on active ubiquitination, as TAK243 treatment significantly reduces MKRN2 partitioning into SGs (Figure 2D-E). However, several mechanistic questions remain:
- Does MKRN2 localize to SGs through binding to ubiquitinated substrates within SGs, or does MKRN2 require its own ubiquitination activity to enter SGs?

- The observation that MKRN2 overexpression increases SG ubiquitin content (Figure 3G-H) could indicate either: (a) MKRN2 actively ubiquitinates substrates within SGs, or (b) MKRN2 recruitment brings along pre-ubiquitinated substrates from the cytoplasm.
- Is MKRN2 localization to SGs dependent on its E3 ligase activity? A catalytically inactive mutant of MKRN2 would help distinguish whether MKRN2 must actively ubiquitinate proteins to remain in SGs or whether it binds to ubiquitinated proteins independently of its catalytic activity.

The authors should clarify whether MKRN2's SG localization depends on its catalytic activity or on binding to ubiquitinated proteins, as this would fundamentally affect the interpretation of its role in SG dynamics.

3. Figures 3-4: Discrepancy between assembly and disassembly phenotypes. MKRN2 knockdown produces distinct phenotypes during SG assembly versus disassembly. During assembly: smaller, more numerous SGs that fail to coalesce (Figure 3A-E), while during disassembly: delayed SG clearance (Figure 4A-D).

These phenotypes may reflect different roles for MKRN2 at different stages, but the mechanism underlying this stage-specificity is unclear:

- Does MKRN2 have different substrates or utilize different ubiquitin chain types during assembly versus disassembly?
- The increased SG liquidity upon MKRN2 depletion (Figure 3F) seems paradoxical with delayed disassembly- typically more liquid condensates disassemble faster. The authors interpret this as decreased coalescence into "dense and mature SGs," but this requires clarification.
- How does prevention of DRiP accumulation relate to the assembly defect? One would predict that DRiP accumulation would primarily affect disassembly (by reducing liquidity), yet MKRN2 depletion impacts both assembly dynamics and DRiP accumulation.

The authors should discuss how MKRN2's role in preventing DRiP accumulation mechanistically connects to both the assembly and disassembly phenotypes.

4. Figure 5: Incomplete characterization of MKRN2 substrates. While the authors convincingly demonstrate that MKRN2 prevents DRiP accumulation in SGs (Figure 5C-D), the direct substrates of MKRN2 remain unknown. The authors acknowledge in the limitations that "the direct MKRN2 substrates and ubiquitin-chain types (K63/K48) are currently unknown." However, several approaches could strengthen the mechanistic understanding:

- Do DRiPs represent direct MKRN2 substrates? Co-immunoprecipitation of MKRN2 followed by ubiquitin-chain specific antibodies (K48 vs K63) could reveal whether MKRN2 mediates degradative (K48) or non-degradative (K63) ubiquitination.
- Given that VCP cofactors (such as UFD1L, PLAA) are depleted from SGs upon UBA1 inhibition (Figure 2C) and these cofactors recognize ubiquitinated substrates, does MKRN2

function upstream of VCP recruitment? Testing whether MKRN2 depletion affects VCP cofactor localization to SGs would clarify this pathway.
- The authors note that MKRN2 knockdown produces a phenotype reminiscent of VCP inhibition-smaller, more numerous SGs with increased DRiP partitioning. This similarity suggests MKRN2 may function in the same pathway as VCP. Direct epistasis experiments would strengthen this connection.
5. Alternative explanations for the phenotype of delayed disassembly with TAK243 or MKRN2 depletion- the authors attribute this to DRiP accumulation, but TAK243 affects global ubiquitination. Could impaired degradation of other SG proteins (not just DRiPs) contribute to delayed disassembly? Does proteasome inhibition (MG-132 treatment) phenocopy the MKRN2 depletion phenotype? This would support that MKRN2-mediated proteasomal degradation (via K48 ubiquitin chains) is key to the phenotype.
6. Comparison with other E3 ligases (Supplementary Figure 5):
The authors show that CNOT4 and ZNF598 depletion also affect SG dynamics, though to lesser extents than MKRN2. However:
- Do these E3 ligases also prevent DRiP accumulation in SGs? Testing OP-puro partitioning in CNOT4- or ZNF598-depleted cells would reveal whether DRiP clearance is a general feature of SG-localized E3 ligases or specific to MKRN2.
- Are there redundant or compensatory relationships between these E3 ligases? Do double knockdowns have additive effects?
- The authors note that MKRN2 is "the most highly SG-depleted E3 upon TAK243 treatment"-does this mean MKRN2 has the strongest dependence on active ubiquitination for its SG localization, or simply that it has the highest basal level of SG partitioning?

2. Significance:

Significance (Required)

This is a well-executed study that identifies MKRN2 as an important regulator of stress granule dynamics and proteostasis. The combination of proximity proteomics, live imaging, and functional assays provides strong evidence for MKRN2's role in preventing DRiP accumulation and maintaining granulostasis. However, key mechanistic questions remain, particularly regarding MKRN2's direct substrates, the ubiquitin chain types it generates, and how its enzymatic activity specifically prevents DRiP accumulation while promoting both SG coalescence and disassembly. Addressing the suggested revisions, particularly those related to MKRN2's mechanism of SG localization and substrate specificity, would significantly strengthen the manuscript and provide clearer insights into how ubiquitination maintains the dynamic properties of stress granules under proteotoxic stress.

3. How much time do you estimate the authors will need to complete the suggested revisions:

Estimated time to Complete Revisions (Required)

(Decision Recommendation)

Between 3 and 6 months

4. *Review Commons* values the work of reviewers and encourages them to get credit for their work. Select 'Yes' below to register your reviewing activity at [Web of Science Reviewer Recognition Service]() (formerly Publons); note that the content of your review will not be visible on Web of Science.

Web of Science Reviewer Recognition

Yes

Review #3

1. Evidence, reproducibility and clarity:

Evidence, reproducibility and clarity (Required)

In this paper, Amzallag et al. investigate the relationship between ubiquitination and the dynamics of stress granules (SGs). They utilize proximity ligation coupled mass spectrometry to identify SG components under conditions where the proteasome is inhibited by a small drug that targets UBiquitin-like modifier Activating enzyme 1 (UBA1), which is crucial for the initial step in the ubiquitination of misfolded proteins. Their findings reveal that the E3 ligase Makorin2 (MKRN2) is a novel component of SGs. Additionally, their data suggest that MKRN2 is necessary for processing damaged ribosome-associated proteins (DRIPs) during heat shock (HS). In the absence of MKRN2, DRIPs accumulate in SGs, which affects their dynamics.

Major comments:

Assess the knockdown efficiency (KD) for CNOT1, ZNF598, and MKRN2 to determine if the significant effect observed on SG dynamics upon MKRN2 depletion is due to the protein's function rather than any possible differences in KD efficiency.
Since HS-induced stress granules (SGs) are influenced by the presence of TAK-243 or

MKRN2 depletion, could it be that these granules become more mature and thus acquire more defective ribosomal products (DRIPs)? Do HS cells reach the same level of DRIPs, as assessed by OP-Puro staining, at a later time point?
Incorporating OP-Puro can lead to premature translation termination, potentially confounding results. Consider treating cells with a short pulse (i.e., 5 minutes) of OP-Puro just before fixation.
Is MKRN2's dependence limited to HS-induced SGs?

Minor comments:

Abstract:

Introduce UBA1.
Introduction:

The reference [2] should be replaced with 25719440.

Results:

Line 70, 'G3BP1 and 2 genes,' is somewhat misleading. Consider rephrasing into 'G3BP1 and G3BP2 genes'.
Line 103: considers rephrasing 'we orthogonally validated the ubiquitin-dependent interaction' to 'we orthogonally validated the ubiquitin-dependent stress granule localization'.
Line 125: '(fig.3C, EI Supplementary fig. 3)' Remove 'I'.
Methods:

line 260: the reference is not linked (it should be ref. [26]).
Line 225: Are all the KDs being performed using the same method? Please specify.

Fig.2C: Consider adding 'DEPLETED' on top of the scheme.

2. Significance:

Significance (Required)

The study offers valuable insights into the degradative processes associated with SGs. The figures are clear, and the experimental quality is high. The authors do not overstate or overinterpret their findings, and the results effectively support their claims. However, the

study lacks orthogonal methods to validate the findings and enhance the results. For instance, incorporating biochemical and reporter-based methods to measure degradation-related intermediate products (DRIPs) would be beneficial. Additionally, utilizing multiple methods to block ubiquitination, studying the dynamics of MKRN2 on SGs, and examining the consequences of excessive DRIPs on the cell fitness of SGs would further strengthen the research.

3. How much time do you estimate the authors will need to complete the suggested revisions:

Estimated time to Complete Revisions (Required)

(Decision Recommendation)

Between 3 and 6 months

4. *Review Commons* values the work of reviewers and encourages them to get credit for their work. Select 'Yes' below to register your reviewing activity at Web of Science Reviewer Recognition Service (formerly Publons); note that the content of your review will not be visible on Web of Science.

Web of Science Reviewer Recognition

Yes

Dear Prof. Hornstein

Thank you for the submission of your manuscript to EMBO reports. I have now read your paper, the accompanying referee reports and your revision plan and discussed it within the editorial team. We all appreciated that you identified MKRN2 as a novel regulator of SG assembly and disassembly but also noted the absence of physiological context provided. I have additionally consulted one of the referees and discussed your study and the advance provided further, taking this limitation into account. Taking all information and feedback into account, we have decided to invite you to revise your study along the lines proposed in your revision plan for potential publication at EMBO reports. Please address all referee concerns in a complete point-by-point response. Acceptance of the manuscript will depend on a positive outcome of a second round of review. It is EMBO reports policy to allow a single round of major revision only and acceptance or rejection of the manuscript will therefore depend on the completeness of your responses included in the next, final version of the manuscript.

We realize that it is difficult to revise to a specific deadline. In the interest of protecting the conceptual advance provided by the work, we recommend a revision within 3 months (30th Apr 2026). Please discuss the revision progress ahead of this time with the editor if you require more time to complete the revisions.

IMPORTANT NOTE: we perform an initial quality control of all revised manuscripts before re-review. Your manuscript will FAIL this control and the handling will be DELAYED if the following APPLIES:

1) A data availability section providing access to data deposited in public databases is missing. If you have not deposited any data, please add a sentence to the data availability section that explains that.
2) Your manuscript contains statistics and error bars based on n=2. Please use scatter blots in these cases. No statistics should be calculated if n=2.

When submitting your revised manuscript, please review the submission guidelines (https://link.springer.com/journal/44319/submission-guidelines#cms-Revised-submissions) and carefully review the instructions that follow below. Failure to include requested items will delay the evaluation of your revision.

1) a .docx formatted version of the manuscript text (including legends for main figures, EV figures and tables). Please make sure that the changes are highlighted to be clearly visible.

2) individual production quality figure files as .eps, .tif, .jpg (one file per figure). See hhttps://media.springernature.com/original/springer-cms/rest/v1/content/27825798/data/v1 for more info on how to prepare your figures.

3) We replaced Supplementary Information with Expanded View (EV) Figures and Tables that are collapsible/expandable online. A maximum of 5 EV Figures can be typeset. EV Figures should be cited as 'Figure EV1, Figure EV2" etc... in the text and their respective legends should be included in the main text after the legends of regular figures.

- For the figures that you do NOT wish to display as Expanded View figures, they should be bundled together with their legends in a single PDF file called *Appendix*, which should start with a short Table of Content. Appendix figures should be referred to in the main text as: "Appendix Figure S1, Appendix Figure S2" etc. See 'Expanded View' section of the submission guidelines.

- Additional Tables/Datasets should be labeled and referred to as Table EV1, Dataset EV1, etc. Legends have to be provided in a separate tab in case of .xls files. Alternatively, the legend can be supplied as a separate text file (README) and zipped together with the Table/Dataset file.

4) a .docx formatted letter INCLUDING the reviewers' reports and your detailed point-by-point responses to their comments. As part of the EMBO Press transparent editorial process, the point-by-point response is part of the Review Process File (RPF), which will be published alongside your paper.

5) a complete author checklist, which you can download from our author guidelines. Please insert information in the checklist that is also reflected in the manuscript. The completed author checklist will also be part of the RPF.

6) Please note that all corresponding authors are required to supply an ORCID ID for their name upon submission of a revised manuscript (), which can be linked to your profile in the manuscript submission system.

7) Before submitting your revision, primary datasets produced in this study need to be deposited in an appropriate public database. Please remember to provide a reviewer password if the datasets are not yet public. The accession numbers and database should be listed in a formal "Data Availability" section placed after Methods. Please note that the Data Availability Section is restricted to new primary data that are part of this study. * Note - All links should resolve to a page where the data can be accessed. *

If your study has not produced novel datasets, please mention this fact in the Data Availability Section.

8) At EMBO Press we ask authors to provide source data for the main manuscript figures. You will receive a separate email with instructions for providing source data with your revised manuscript, including how to upload and organize the files.

9) Our journal also encourages inclusion of *data citations in the reference list* to directly cite datasets that were re-used and obtained from public databases. Data citations in the article text are distinct from normal bibliographical citations and should directly link to the database records from which the data can be accessed. In the main text, data citations are formatted as follows: "Data ref: Smith et al, 2001" or "Data ref: NCBI Sequence Read Archive PRJNA342805, 2017". In the Reference list, data citations must be labeled with "[DATASET]". A data reference must provide the database name, accession number/identifiers and a resolvable link to the landing page from which the data can be accessed at the end of the reference.

10) Regarding data quantification, the following points must be specified in each figure legend:

- the name of the statistical test used to generate error bars and P values,

- the number (n) of independent experiments (please specify technical or biological replicates) underlying each data point,

- the nature of the bars and error bars (s.d., s.e.m.),

- If the data are obtained from n Program fragment delivered error ``Can't locate object method "less" via package "than" (perhaps you forgot to load "than"?) at //ejpvfs23/sites23b/embor_www/letters/embor_decision_rc_revise_and_rereview.txt line 54.' 2, use scatter blots showing the individual data points.

Discussion of statistical methodology can be reported in the materials and methods section, but figure legends should contain a basic description of n, P and the test applied.

- Please also include scale bars in all microscopy images.

11) The journal requires a statement specifying whether or not authors have competing interests (defined as all potential or actual interests that could be perceived to influence the presentation or interpretation of an article). In case of competing interests, this must be specified in your disclosure statement.

12) All Materials and Methods need to be described in the main text using our 'Structured Methods' format, which is required for all research articles. According to this format, the Methods section includes a Reagents and Tools Table (listing key reagents, experimental models, software and relevant equipment and including their sources and relevant identifiers) followed by a Methods and Protocols section describing the methods using a step-by-step protocol format. The aim is to facilitate adoption of the methodologies across labs. More information on how to adhere to this format as well as a downloadable template (.docx) for the Reagents and Tools Table can be found in our author guidelines.

We would also welcome the submission of cover suggestions, or motifs to be used by our Graphics Illustrator in designing a cover.

As part of the EMBO publication's Transparent Editorial Process, EMBO reports publishes online a Review Process File (RPF) to accompany accepted manuscripts. This File will be published in conjunction with your paper and will include the referee reports, your point-by-point response and all pertinent correspondence relating to the manuscript.

You are able to opt out of this by letting the editorial office know (emboreports@embo.org). If you do opt out, the Review Process File link will point to the following statement: "No Review Process File is available with this article, as the authors have chosen not to make the review process public in this case."

I look forward to seeing a revised form of your manuscript when it is ready.

Yours sincerely,

Martina Rembold, PhD
Senior Editor
EMBO reports

Manuscript number: RC-2025-03277

# Corresponding author(s): Eran Hornstein

1. General Statements [optional]

Dear Editor,

Below is a point-by-point reply to the reviewer comments for our manuscript entitled "Ubiquitination regulates granulostasis and DRiP accumulation in SGs under heat stress via the E3 ligase MKRN2" by Amzallag et al.

Our study performed proteomic profiling of stress granules following small molecule inhibition of the E1 ubiquitin ligase enzyme UBA1, where we discovered that several proteostasis factors localized to stress granules in a UBA1-dependent manner. In the latter part of our study, we used a variety of microscopy techniques to perform a focused analysis of the effect of depletion of the E3 ligase MKRN2 on stress granule homeostasis, which was a prominent hit in our proteomics screen. Our study's central finding is that MKNR2 is the first E3 ligase, regulating the accumulation of misfolding-prone peptides (DRiPs) in stress granules.

As agreed in the revisions plan, we note that Reviewer #2 requested that we explicitly define the specific MKRN2 substrates, determine whether MKRN2 mediates K48- or K63-linked ubiquitination, and further characterize other E3 ligases identified in the initial screen. While interesting, these requests go beyond the scope of our original aims and conclusions and hence they were not addressed in part of the current manuscript.

Eran Hornstein, MD/ PhD

Weizmann Institute of Science

2. Point-by-point description of the revisions

Reviewer #1 (Evidence, reproducibility and clarity (Required)):

Summary:

In this study, the authors used proximity proteomics in U2OS cells to identify several E3 ubiquitin ligases recruited to stress granules (SGs), and they focused on MKRN2 as a novel regulator. They show that MKRN2 localization to SGs requires active ubiquitination via UBA1. Functional experiments demonstrated that MKRN2 knockdown increases the number of SG condensates, reduces their size, slightly raises SG liquidity during assembly, and slows disassembly after heat shock. Overexpression of MKRN2-GFP combined with confocal imaging revealed co-localization of MKRN2 and ubiquitin in SGs. By perturbing ubiquitination (using a UBA1 inhibitor) and inducing defective ribosomal products (DRiPs) with O-propargyl puromycin, they found that both ubiquitination inhibition and MKRN2 depletion lead to increased accumulation of DRiPs in SGs. The authors conclude that MKRN2 supports granulostasis, the maintenance of SG homeostasis , through its ubiquitin ligase activity, preventing pathological DRiP accumulation within SGs.

Authors: We thank Reviewer #1 for their insightful and positive review.

Major comments:

- Are the key conclusions convincing?

The key conclusions are partially convincing. The data supporting the role of ubiquitination and MKRN2 in regulating SG condensate dynamics are coherent, well controlled, and consistent with previous literature, making this part of the study solid and credible. However, the conclusions regarding the ubiquitin-dependent recruitment of MKRN2 to SGs, its relationship with UBA1 activity, the functional impact of the MKRN2 knockdown for DRiP accumulation are less thoroughly supported. These aspects would benefit from additional mechanistic evidence, validation in complementary model systems, or the use of alternative methodological approaches to strengthen the causal connections drawn by the authors.

- Should the authors qualify some of their claims as preliminary or speculative, or remove them altogether?

The authors should qualify some of their claims as preliminary.

1) MKRN2 recruitment to SGs (ubiquitin-dependent): The proteomics and IF data are a reasonable starting point, but they do not yet establish that MKRN2 is recruited from its physiological localization to SGs in a ubiquitin-dependent manner. To avoid overstating this point the authors should qualify the claim and/or provide additional controls: show baseline localization of endogenous MKRN2 under non-stress conditions (which is reported in literature to be nuclear and cytoplasmatic), include quantification of nuclear/cytoplasmic distribution, and demonstrate a shift into bona fide SG compartments after heat shock.

Moreover, co-localization of overexpressed GFP-MKRN2 with poly-Ub (FK2) should be compared to a non-stress control and to UBA1-inhibition conditions to support claims of stress- and ubiquitination-dependent recruitment.

Authors: We have characterized the subcellular localization of endogenous MKRN2 at both basal state and under heat stress to provide better context for our subsequent observations (Fig. S6).

2) Use and interpretation of UBA1 inhibition: UBA1 inhibition effectively blocks ubiquitination globally, but it is non-selective. The manuscript should explicitly acknowledge this limitation when interpreting results from both proteomics and functional assays. Proteomics hits identified under UBA1 inhibition should be discussed as UBA1-dependent associations rather than as evidence for specific E3 ligase recruitment. The authors should consider orthogonal approaches before concluding specificity.

Authors: We have acknowledged the limitation of using only TAK243 in our study by rephrasing statements about dependency on “ubiquitination” to “UBA1-dependent associations”.

3) DRiP accumulation and imaging quality: The evidence presented in Figure 5 is sufficient to substantiate the claim that DRiPs accumulate in SGs upon ubiquitination inhibition or MKRN2 depletion but to show that the event of the SGs localization and their clearance from SGs during stress is promoted by MKRN3 ubiquitin ligase activity more experiments would be needed.

Authors: As suggested by the reviewer, rescue experiments would entail high time and resource investment along with requiring specific expertise for validation of the mutant’s loss of ubiquitination activity, preventing us from pursuing this experimental avenue at this time. As per the reviewer’s suggestion we have qualified our statements regarding MKRN2’s activity on

DRiPs in SGs to reflect this reality. We refer to MKRN2 as “preventing accumulation of DRiPs in SGs” instead of “promoting DRiPs clearance from SGs”. This phrasing is more conservative in its interpretation of the data. We have also explicitly stated in the Discussion section that we cannot conclusively claim that MKRN2’s effects on SGs result from its own ubiquitination activity (see lines 261-264 and 288-289).

- Would additional experiments be essential to support the claims of the paper? Request additional experiments only where necessary for the paper as it is, and do not ask authors to open new lines of experimentation.

Yes, a few targeted experiments would strengthen the conclusions without requiring the authors to open new lines of investigation.

1) Baseline localization of MKRN2: It would be important to show the baseline localization of endogenous and over-expressed MKRN2 (nuclear and cytoplasmic) under non-stress conditions and prior to ubiquitination inhibition. This would provide a reference to quantify redistribution into SGs and demonstrate recruitment in response to heat stress or ubiquitination-dependent mechanisms.

Authors: We thank the reviewer for bringing this important control. We have performed microscopic analysis of endogenous MKRN2 immunofluorescent staining and overexpressed MKRN2-GFP. Both endogenous MKRN2 and overexpressed MKRN2-GFP exhibited predominantly cytoplasmic localization with some nuclear presence as well, in agreement with published observations (Shin et al. 2017; Wolf et al. 2020). These results are summarized in Fig S6 and S11.

2) Specificity of MKRN2 ubiquitin ligase activity: to address the non-specific effects of UBA1 inhibition and validate that observed phenotypes depend on MKRN2's ligase activity, the authors could employ a catalytically inactive MKRN2 mutant in rescue experiments. Comparing wild-type and catalytic-dead MKRN2 in the knockdown background would clarify the causal role of MKRN2 activity in SG dynamics and DRiP clearance.

Authors: In accordance with Reviewer #1 advice below, we agree the experiments proposed represents a higher resource and time investment. Therefore, we have relaxed our claims of MKRN2-mediated ubiquitination in the Discussion to reflect the lack of these experiments: “Therefore, these data suggest that MKRN2 regulates the accumulation of a subset of DRiPs into SGs, plausibly via ubiquitination activity.” (lines 201-202), “Notably, we cannot conclusively determine that MKRN2-mediated prevention of DRiP accumulation in SGs results from its own ubiquitination-ligase activity, since we have not analyzed MKRN2 catalytic functions. These experiments should be followed up in subsequent studies.” (lines 261-264) and ”Additionally, we

have not demonstrated a conclusive association of MKRN2 catalytic activity with its SG-related functions." (lines 287-288).

3) Ubiquitination linkage and SG marker levels: While the specific ubiquitin linkage type remains unknown, examining whether MKRN2 knockdown or overexpression affects total levels of key SG marker proteins would be informative. This could be done via Western blotting of SG markers along with ubiquitin staining, to assess whether MKRN2 influences protein stability or turnover through degradative or non-degradative ubiquitination. Such data would strengthen the mechanistic interpretation while remaining within the current study's scope.

Authors: We addressed the reviewer request by performing MKRN2 KD. No significant difference in G3BP1 expression could be seen via Western blotting following MKRN2 KD. These data are summarized in Fig S5. Thus, MKRN2 likely does not function in the degradation of G3BP1. Characterizing whether MKRN2 affects specific ubiquitin linkage types, while interesting, is not within the scope of the current study.

- Are the suggested experiments realistic in terms of time and resources? It would help if you could add an estimated cost and time investment for substantial experiments.

The experiments suggested in points 1 and 3 are realistic and should not require substantial additional resources beyond those already used in the study.

• Point 1 (baseline localization of MKRN2): This involves adding two control conditions (no stress and no ubiquitination inhibition) for microscopy imaging. The setup is essentially the same as in the current experiments, with time requirements mainly dependent on cell culture growth and imaging. Overall, this could be completed within a few weeks.

Authors: The baseline localization of both endogenous (Fig. S6) and overexpressed MKRN2-GFP (Fig. S11) were characterized in accordance with the reviewer's request. MKRN2 at baseline is predomionantly cytoplasmic, but is evident also in the nucleus as previously reported.

• Point 3 (SG marker levels and ubiquitination): This entails repeating the existing experiment and adding a Western blot for SG markers and ubiquitin. The lab should already have the necessary antibodies, and the experiment could reasonably be performed within a couple of weeks.

Authors: We have analyzed the protein expression level of the central SG nucleating protein G3BP1 and found that MKRN2 does not affect its expression level (Fig. S5A). Characterizing ubiquitin linkage types associated with MKRN2 would be interesting, however is beyond the scope of the current study.

• Point 2 (catalytically inactive MKRN2 mutant and rescue experiments): This is likely more time-consuming. Designing an effective catalytic-dead mutant depends on structural knowledge of MKRN2 and may require additional validation to confirm loss of catalytic activity. If this expertise is not already present in the lab, it could significantly extend the timeline. Therefore, this experiment should be considered only if similarly recommended by other reviewers, as it represents a higher resource and time investment.

Authors: We agree with the reviewer that this would be too time consuming at this stage. We have instead qualified our statements on the role of MKRN2 on DRiPs and SGs to reflect this reality. We also clearly state this caveat of our study in the Discussion (lines 201-202, 261-264 and 287-289).

Overall, points 1 and 3 are highly feasible, while point 2 is more substantial and may require careful planning.

- Are the data and the methods presented in such a way that they can be reproduced?

Yes. The methodologies used in this study to analyze SG dynamics and DRiP accumulation are well-established in the field and should be reproducible, particularly by researchers experienced in stress granule biology. Techniques such as SG assembly and disassembly assays, use of G3BP1 markers, and UBA1 inhibition are standard and clearly described. The data are generally presented in a reproducible manner; however, as noted above, some results would benefit from additional controls or complementary experiments to fully support specific conclusions.

- Are the experiments adequately replicated and statistical analysis adequate?

Overall, the experiments in the manuscript appear to be adequately replicated, with most assays repeated between three and five times, as indicated in the supplementary materials. The statistical analyses used are appropriate and correctly applied to the datasets presented. However, for Figure 5 the number of experimental replicates is not reported. This should be clarified, and if the experiment was not repeated sufficiently, additional biological replicates should be performed. Given that this figure provides central evidence

supporting the conclusion that DRiP accumulation depends on ubiquitination-and partly on MKRN2's ubiquitin ligase activity-adequate replication is essential.

Authors: The number of experimental repeats was added to the Figure 5 legend and both Fig. 5B and 5D consist of three experimental repeats each.

Minor comments:

- Specific experimental issues that are easily addressable.

• For the generation and the validation of MKRN2 knockdown in UOS2 cells data are not presented in the results or in the methods sections to demonstrate the effective knockdown of the protein of interest. This point is quite essential to demonstrate the validity of the system used

Authors: We addressed this request by performing MKRN2 KD. Western blot (Fig S5B, roughly fourfold) and RT-qPCR (Fig S4C, roughly 90% reduction). These new data reveal that MKRN2 is effectively knocked down.

• In the supplementary figure 2 it would be useful to mention if the Western Blot represent the input (total cell lysates) before the APEX-pulldown or if it is the APEX-pulldown loaded for WB. There is no consistence in the difference of biotynilation between different replicates shown in the 2 blots. For example in R1 and R2 G3BP1-APX TAK243 the biotynilation is one if the strongest condition while on the left blot, in the same condition comparison samples R3 and R4 are less biotinilated compared to others. It would be useful to provide an explanation for that to avoid any confusion for the readers.

Authors: We have added a mention in the legend of Figure S2 that these are total cell lysates before pulldown. The apparent differences in biotin staining are small and not sufficient to question the results of our APEX-proteomics.

• In Figure 2D, endogenous MKRN2 localization to SGs appears reduced following UBA1 inhibition. However, it is not clear whether this reduction reflects a true relocalization or a decrease in total MKRN2 protein levels. To support the interpretation that UBA1 inhibition specifically affects MKRN2 recruitment to SGs rather than its overall expression, the authors should provide data showing total MKRN2 levels remain unchanged under UBA1 inhibition, for example via Western blot of total cell lysates.

Authors: Based on first principles in regulation of gene expression, it is unlikely that total MKRN2 expression levels would decrease appreciably through transcriptional or translational

regulation within the short timescale of these experiments (1 h TAK243 pretreatment followed by 90 min of heat stress). Therefore, the decreased partitioning of MKRN2 to SGs following UBA1 inhibition plausibly reflects a change in protein localization.

• DRIPs accumulation is followed during assembly but in the introduction is highlighted the fact that ubiquitination events, other reported E3 ligases and in this study data on MKRN2 showed that they play a crucial role in the disassembly of SGs which is also related with cleareance of DRIPs. Authors could add tracking DRIPs accumulation during disassembly to be added to Figure 5. I am not sure about the timeline required for this but I am just adding as optional if could be addressed easily.

Authors: We thank the reviewer for proposing this experimental direction. However, in our previous study (Ganassi et al. 2016), we demonstrated that DRiP accumulation during the stress granule assembly phase drives conversion to a solid-like state and delays stress granule disassembly. It is therefore critical to assess DRiP enrichment within stress granules immediately after their formation, rather than during the stress recovery phase, as done here.

• The authors should clarify in the text why the cutoff used for the quantification in Figure 5D (PC > 3) differs from the cutoff used elsewhere in the paper (PC > 1.5). Providing a rationale for this choice will help the reader understand the methodological consistency and ensure that differences in thresholds do not confound interpretation of the results.

Authors: We thank the reviewer for this question. The population of SGs with a DRiP enrichment > 1.5 represents SGs with a significant DRiP enrichment compared to the surrounding (background) signal. As explained in the methods, the intensity of DRiPs inside each SG is corrected by the intensity of DRiPs two pixels outside of each SG. Thus, differences in thresholds between independent experimental conditions (5B versus 5D) do not confound interpretation of the results but depend on overall staining intensity that can different between different experimental conditions. Choosing the cut-off > 3 allows to specifically highlight the population of SGs that are strongly enriched with DRiPs. MKRN2 silencing caused a strong DRiP enrichment in the majority of the SGs analyzed and therefore we chose this way of data representation. Note that the results represent the average of the analysis of 3 independent experiments with high numbers of SGs automatically segmented and analyzed/experiment. Figure 5A, B: n = 3 independent experiments; number of SGs analyzed per experiment: HS + OP-puro (695; 1216; 952); TAK-243 + HS + OP-puro (1852; 2214; 1774). Figure 5C, D: n = 3 independent experiments; number of SGs analyzed per experiment: siRNA control, HS + OP-puro (1984; 1400; 1708); siRNA MKRN2, HS + OP-puro (912; 1074; 1532).

• For Figure 3G, the authors use over-expressed MKRN2-GFP to assess co-localization with ubiquitin in SGs. Given that a reliable antibody for endogenous MKRN2 is available and that

a validated MKRN2 knockdown line exists as an appropriate control, this experiment would gain significantly in robustness and interpretability if co-localization were demonstrated using endogenous MKRN2. In the current over-expression system, MKRN2-GFP is also present in the nucleus, whereas the endogenous protein does not appear nuclear under the conditions shown. This discrepancy raises concerns about potential over-expression artifacts or mislocalization. Demonstrating co-localization using endogenous MKRN2 would avoid confounding effects associated with over-expression. If feasible, this would be a relatively straightforward experiment to implement, as it relies on tools (antibody and knockdown line) already described in the manuscript.

Authors: We thank the reviewer. We addressed the request by MKRN2 KD that was followed by performing ubiquitin (FK2) immunofluorescence microscopy and SG partition coefficient analysis. Using this assay we could not detect a significant decrease in ubiquitin partitioning to SGs following MKRN2 KD (student's t-test p=0.2231). These results are summarized in fig S7A and S7B. We conclude that "Together these data indicate that MKRN2 promotes SG coalescence during HS and SG disassembly following recovery from HS and may be associated with increased ubiquitination within SGs, as suggested by co-localization. While MKRN2 is sufficient to drive ubiquitin partitioning into SGs, it is not strictly necessary, perhaps due to functional redundancy with alternative E3 ligases." (lines 180-184).

- Are prior studies referenced appropriately?

• From line 54 to line 67, the manuscript in total cites eight papers regarding the role of ubiquitination in SG disassembly. However, given the use of UBA1 inhibition in the initial MS-APEX experiment and the extensive prior literature on ubiquitination in SG assembly and disassembly under various stress conditions, the manuscript would benefit from citing additional relevant studies to provide more specifc examples. Expanding the references would provide stronger context, better connect the current findings to prior work, and emphasize the significance of the study in relation to established literature

Authors: We have added citations for the relevant studies.

• At line 59, it would be helpful to note that G3BP1 is ubiquitinated by TRIM21 through a Lys63-linked ubiquitin chain. This information provides important mechanistic context, suggesting that ubiquitination of SG proteins in these pathways is likely non-degradative and related to functional regulation of SG dynamics rather than protein turnover.

Authors: The reviewer is correct. We have added to the text that G3BP1 is ubiquitinated through a Lys63-linked ubiquitin chain.

• When citing references 16 and 17, which report that the E3 ligases TRIM21 and HECT regulate SG formation, the authors should provide a plausible explanation for why these specific E3 ligases were not detected in their proteomics experiments. Differences could arise from the stress stimulus used, cell type, or experimental conditions. Similarly, since MKRN2 and other E3 ligases identified in this study have not been reported in previous works, discussing these methodological or biological differences would help prevent readers from questioning the credibility of the findings. It would also be valuable to clarify in the Conclusion that different types of stress may activate distinct ubiquitination pathways, highlighting context-dependent regulation of SG assembly and disassembly.

Authors: We thank the reviewer for this suggestion. We added to the discussion plausible explanations for why our study identified new E3 ligases.

• Line 59-60: when referring to the HECT family of E3 ligases involved in ubiquitination and SG disassembly, it would be more precise to report the specific E3 ligase identified in the cited studies rather than only the class of ligase. This would provide clearer mechanistic context and improve accuracy for readers.

Authors: We have added this detail to the introduction.

• The specific statement on line 182 "SG E3 ligases that depend on UBA1 activity are RBULs" should be supported by reference.

Authors: We have added citations to back up our claim that ZNF598, CNOT4, MKRN2, TRIM25 and TRIM26 exhibit RNA-binding activity.

- Are the text and figures clear and accurate?

• In Supplementary Figure 1, DMSO is shown in green and the treatment in red, whereas in the main figures (Figure 1B and 1F) the colours in the legend are inverted. To avoid confusion, the colour coding in figure legends should be consistent across all figures throughout the manuscript.

Authors: We have made the colors consistent across the main and supplementary figures.

• At line 79, the manuscript states that "inhibition of ubiquitination delayed fluorescence recovery dynamics of G3BP1-mCherry, relative to HS-treated cells (Figure 1F, Supplementary Fig. 6A)." However, the data shown in Figure 1F appear to indicate the opposite effect: the TAK243-treated condition (green curve) shows a faster fluorescence

recovery compared to the control (red curve). This discrepancy between the text and the figure should be corrected or clarified, as it may affect the interpretation of the role of ubiquitination in SG dynamics.

Authors: Good catch. We now fixed the graphical mistake (Figure 1F and S6).

• Line 86: adjust a missing bracket

Authors: Thank you, we fixed it.

• There appears to be an error in the legend of Supplementary Figure 3: the legend states that the red condition (MKRN2) forms larger aggregates, but both the main Figure 3C of the confocal images and the text indicate that MKRN2 (red) forms smaller aggregates. Please correct the legend and any corresponding labels so they are consistent with the main figure and the text. The authors should also double-check that the figure panel order, color coding, and statistical annotations match the legend and the descriptions in the Results section to avoid reader confusion.

Authors: This unfortunate graphical mistake has been corrected.

• At lines 129-130, the manuscript states that "FRAP analysis demonstrated that MKRN2 KD resulted in a slight increase in SG liquidity (Fig. 3F, Supplementary Fig. 6B)." However, the data shown in Figure 3F appear to indicate the opposite trend: the MKRN2 KD condition (red curve) exhibits a faster fluorescence recovery compared to the control (green curve). This discrepancy between the text and the figure should be corrected or clarified, as it directly affects the interpretation of MKRN2's role in SG disassembly. Ensuring consistency between the written description and the plotted FRAP data is essential for accurate interpretation.

Authors: We thank the reviewer and clarify in the legend of Figure 3F and the Results the correct labels: indeed faster fluorescence recovery seen in MKRN2 KD is correctly interpreted as increased liquidity in the text.

• At lines 132-133, the manuscript states: "Then, to further test the impact of MKRN2 on SG dynamics, we overexpressed MKRN2-GFP and observed that it was recruited to SG (Fig. 3G)." This description should be corrected or clarified, as the over-expressed MKRN2-GFP also appears to localize to the nucleus.

Authors: The text has been modified to reflect both the study of MKRN2 localization to SGs and of nuclear localization. “To further test the impact of MKRN2 on SG dynamics, we

overexpressed MKRN2–GFP, which exhibited both nuclear and cytoplasmic localization, and was recruited to SGs under heat stress (Fig. 3G).” (lines 174-176)

• At lines 134-135, the manuscript states that the FK2 antibody detects "free ubiquitin." This is incorrect. FK2 does not detect free ubiquitin; it recognizes only ubiquitin conjugates, including mono-ubiquitinated and poly-ubiquitinated proteins. The text should be corrected accordingly to avoid misinterpretation of the immunostaining data.

Authors: Thank you for pointing out this error. We have corrected it.

• Figure 5A suffers from poor resolution, and no scale bar is provided, which limits interpretability. Additionally, the ROI selected for the green channel (DRIPs) appears to capture unspecific background staining, while the most obvious DRIP spots are localized in the nucleus. The authors should clarify this in the text, improve the image quality if possible, and ensure that the ROI accurately represents DRIP accumulation - in SGs rather than background signal.

Authors: We thank the reviewer for pointing the sub-optimal presentation of this figure. We modified Figure 5A to improve image quality and interpretation. Concerning the comment that “the most obvious DRIP spots are localized in the nucleus”, this is in line with our previous findings demonstrating that a fraction of DRiPs accumulates in nucleoli (Mediani et al. 2019 10.15252/embj.2018101341). To avoid misinterpretation, we modified Figure 5A as follows: (i) we provide a different image for control cells, exposed to heat shock and OP-puro; (ii) we select a ROI that only shows a few stress granules; (iii) we added arrowheads to indicate the nucleoli that are strongly enriched for DRiPs; (iv) we include a dotted line to show the nuclear membrane, helping to distinguish cytoplasm and nucleus in the red and green channel. We also include the scale bars (5 μm) in the image.

Do you have suggestions that would help the authors improve the presentation of their data and conclusions?

• In the first paragraph following the APEX proteomics results, the authors present validation data exclusively for MKRN2, justifying this early focus by stating that MKRN2 is the most SG-depleted E3 ligase. However, in the subsequent paragraph they introduce the RBULs and present knockdown data for MKRN2 along with two additional E3 ligases identified in the screen, before once again emphasizing that MKRN2 is the most SG-depleted ligase and therefore the main focus of the study. For clarity and logical flow, the manuscript would benefit from reordering the narrative. Specifically, the authors should first present the validation data for all three selected E3 ligases, and only then justify the

decision to focus on MKRN2 for in-depth characterization. In addition to the extent of its SG depletion, the authors may also consider providing biologically relevant reasons for prioritizing MKRN2 (e.g., domain architecture, known roles in stress responses, or prior evidence of ubiquitination-related functions). Reorganizing this section would improve readability and better guide the reader through the rationale for the study's focus.

Authors: We thank the reviewer for this suggested improvement to our “storyline”. As suggested by the reviewer, we have moved the IF validation of MKRN2 to the following paragraph in order to improve the flow of the manuscript. We added additional justification to prioritizing MKRN2 citing (Youn et al. 2018; Markmiller et al. 2018).

• At lines 137-138, the manuscript states: "Together these data indicate that MKRN2 regulates the assembly dynamics of SGs by promoting their coalescence during HS and can increase SG ubiquitin content." While Figure 3G shows some co-localization of MKRN2 with ubiquitin, immunofluorescence alone is insufficient to claim an increase in SG ubiquitin content. This conclusion should be supported by orthogonal experiments, such as Western blotting, in vitro ubiquitination assays, or immunoprecipitation of SG components. Including a control under no-stress conditions would also help demonstrate that ubiquitination increases specifically in response to stress. The second part of the statement should therefore be rephrased to avoid overinterpretation, for example:"...and may be associated with increased ubiquitination within SGs, as suggested by co-localization, pending further validation by complementary assays."

Authors: The statement has been rephrased in a softer way as suggested by the reviewer: “Together these data indicate that MKRN2 promotes SG coalescence during HS and SG disassembly following recovery from HS and may be associated with increased ubiquitination within SGs, as suggested by co-localization.” (lines 180-182).

• At line 157, the statement: "Therefore, we conclude that MKRN2 ubiquitinates a subset of DRiPs, avoiding their accumulation inside SGs" should be rephrased as a preliminary observation. While the data support a role for MKRN2 in SG disassembly and a reduction of DRIPs, direct ubiquitination of DRIPs by MKRN2 has not been demonstrated. A more cautious phrasing would better reflect the current evidence and avoid overinterpretation.

Authors: We thank the reviewer. We have altered the statement accordingly: “Therefore, these data suggest that MKRN2 regulates the accumulation of a subset of DRiPs into SGs, plausibly via ubiquitination activity.” (lines 201-202). We have also further qualified our statements on MKRN2 activity on DRiPs in SGs as “preventing accumulation” rather than “promoting clearance” of DRiPs, avoiding over-interpretation of the data.

Reviewer #1 (Significance (Required)):

General assessment: provide a summary of the strengths and limitations of the study. What are the strongest and most important aspects? What aspects of the study should be improved or could be developed?

• This study provides a valuable advancement in understanding the role of ubiquitination in stress granule (SG) dynamics and the clearance of SGs formed under heat stress. A major strength is the demonstration of how E3 ligases identified through proteomic screening, particularly MKRN2, influence SG assembly and disassembly in a ubiquitination- and heat stress-dependent manner. The combination of proteomics, imaging, and functional assays provides a coherent mechanistic framework linking ubiquitination to SG homeostasis. Limitations of the study include the exclusive use of a single model system (U2OS cells), which may limit generalizability. Additionally, some observations-such as MKRN2-dependent ubiquitination within SGs and changes in DRIP accumulation under different conditions-would benefit from orthogonal validation experiments (e.g., Western blotting, immunoprecipitation, or in vitro assays) to confirm and strengthen these findings. Addressing these points would enhance the robustness and broader applicability of the conclusions.

Advance: compare the study to the closest related results in the literature or highlight results reported for the first time to your knowledge; does the study extend the knowledge in the field and in which way? Describe the nature of the advance and the resulting insights (for example: conceptual, technical, clinical, mechanistic, functional,...).

• The closest related result in literature is - Yang, Cuiwei et al. "Stress granule homeostasis is modulated by TRIM21-mediated ubiquitination of G3BP1 and autophagy-dependent elimination of stress granules." Autophagy vol. 19,7 (2023): 1934-1951. doi:10.1080/15548627.2022.2164427 - demonstrating that TRIM21, an E3 ubiquitin ligase, catalyzes K63-linked ubiquitination of G3BP1, a core SG nucleator, under oxidative stress. This ubiquitination by TRIM21 inhibits SG formation, likely by altering G3BP1's propensity for phase separation. In contrast, the MKRN2 study identifies a different E3 (MKRN2) that regulates SG dynamics under heat stress and appears to influence both assembly and disassembly. This expands the role of ubiquitin ligases in SG regulation beyond those previously studied (like TRIM21).

• Gwon and colleagues (Gwon Y, Maxwell BA, Kolaitis RM, Zhang P, Kim HJ, Taylor JP. Ubiquitination of G3BP1 mediates stress granule disassembly in a context-specific manner.

Science. 2021;372(6549):eabf6548. doi:10.1126/science.abf6548) have shown that K63-linked ubiquitination of G3BP1 is required for SG disassembly after heat stress. This ubiquitinated G3BP1 recruits the segregase VCP/p97, which helps extract G3BP1 from SGs for disassembly. The MKRN2 paper builds on this by linking UBA1-dependent ubiquitination and MKRN2's activity to SG disassembly. Specifically, they show MKRN2 knockdown affects disassembly, and suggest MKRN2 helps prevent accumulation of defective ribosomal products (DRiPs) in SGs, adding a new layer to the ubiquitin-VCP model.

• Ubiquitination's impact is highly stress- and context-dependent (different chain types, ubiquitin linkages, and recruitment of E3s). The MKRN2 work conceptually strengthens this idea: by showing that MKRN2's engagement with SGs depends on active ubiquitination via UBA1, and by demonstrating functional consequences (SG dynamics + DRIP accumulation), the study highlights how cellular context (e.g., heat stress) can recruit specific ubiquitin ligases to SGs and modulate their behavior.

• There is a gap in the literature: very few (if any) studies explicitly combine the biology of DRIPs, stress granules, and E3 ligase mediated ubiquitination, especially in mammalian cells. There are relevant works about DRIP biology in stress granules, but those studies focus on chaperone-based quality control, not ubiquitin ligase-mediated ubiquitination of DRIPs. This study seems to be one of the first to make that connection in mammalian (or human-like) SG biology. A work on the plant DRIP-E3 ligase TaSAP5 (Zhang N, Yin Y, Liu X, et al. The E3 Ligase TaSAP5 Alters Drought Stress Responses by Promoting the Degradation of DRIP Proteins. Plant Physiol. 2017;175(4):1878-1892. doi:10.1104/pp.17.01319) shows that DRIPs can be directly ubiquitinated by E3s in other biological systems - which supports the plausibility of the MKRN2 mechanism, but it's not the same context.

• A very recent review (Yuan, Lin et al. "Stress granules: emerging players in neurodegenerative diseases." Translational neurodegeneration vol. 14,1 22. 12 May. 2025, doi:10.1186/s40035-025-00482-9) summarizes and reinforces the relationship among SGs and the pathogenesis of different neurodegenerative diseases (NDDs). By identifying MKRN2 as a new ubiquitin regulator in SGs, the current study could have relevance for neurodegeneration and proteotoxic diseases, providing a new candidate to explore in disease models.

Audience: describe the type of audience ("specialized", "broad", "basic research", "translational/clinical", etc...) that will be interested or influenced by this research; how will this research be used by others; will it be of interest beyond the specific field?

The audience for this paper is primarily specialized, including researchers in stress granule biology, ubiquitin signaling, protein quality control, ribosome biology, and cellular stress responses. The findings will also be of interest to scientists working on granulostasis, nascent protein surveillance, and proteostasis mechanisms. Beyond these specific fields, the study provides preliminary evidence linking ubiquitination to DRIP handling and SG dynamics, which may stimulate new research directions and collaborative efforts across complementary areas of cell biology and molecular biology.

- Please define your field of expertise with a few keywords to help the authors contextualize your point of view. Indicate if there are any parts of the paper that you do not have sufficient expertise to evaluate.

I work in ubiquitin biology, focusing on ubiquitination signaling in physiological and disease contexts, with particular expertise in the identification of E3 ligases and their substrates across different cellular systems and in vivo models. I have less expertise in stress granule dynamics and DRiP biology, so my evaluation of those aspects is more limited and relies on interpretation of the data presented in the manuscript.

Authors: We thank reviewer #1 for a thorough and overall favorable review.

Reviewer #2 (Evidence, reproducibility and clarity (Required)):

This study identifies the E3 ubiquitin ligase Makorin 2 (MKRN2) as a novel regulator of stress granule (SG) dynamics and proteostasis. Using APEX proximity proteomics, the authors demonstrate that inhibition of the ubiquitin-activating enzyme UBA1 with TAK243 alters the SG proteome, leading to depletion of several E3 ligases, chaperones, and VCP cofactors. Detailed characterization of MKRN2 reveals that it localizes to SGs in a ubiquitination-dependent manner and is required for proper SG assembly, coalescence, and disassembly. Functionally, MKRN2 prevents the accumulation of defective ribosomal products (DRiPs) within SGs, thereby maintaining granulostasis. The study provides compelling evidence that ubiquitination, mediated specifically by MKRN2, plays a critical role in surveilling stress-damaged proteins within SGs and maintaining their dynamic liquid-like properties.

Major issues:

1. Figures 1-2: Temporal dynamics of ubiquitination in SGs. The APEX proteomics was performed at a single timepoint (90 min heat stress), yet the live imaging data show that SG dynamics and TAK243 effects vary considerably over time:

• The peak or SG nucleation was actually at 10-30 min (Figure 1B).

Authors: This remark illustrates a crucial aspect of SG biology: the peak at 10–30 min represents the maximum number of early, immature SGs. As previously described (Wheeler et al. 2016), these granules subsequently undergo maturation and fusion. While SG number and area both increase during the immature phase, the maturation phase is characterized by fusion; consequently, SG numbers drop even as total mass continues to grow. Thus, the decrease in SG count through 90 min of heat stress reflects their consolidation into mature SGs.

• TAK243 treatment causes earlier SG nucleation (Figure 1B) but delayed disassembly (Figure 1A-B, D).

A temporal proteomic analysis at multiple timepoints (e.g., 30 min, 60 min, 90 min of heat stress, and during recovery) would reveal whether MKRN2 and other ubiquitination-dependent proteins are recruited to SGs dynamically during the stress response. It would also delineate whether different E3 ligases predominate at different stages of the SG lifecycle.

While such experiments may be beyond the scope of the current study, the authors should at minimum discuss this limitation and acknowledge that the single-timepoint analysis may miss dynamic changes in SG composition.

Authors: We thank the reviewer for identifying this caveat in our methodology. We now discuss this limitation and acknowledge that the single-timepoint analysis may miss dynamic changes in SG composition (lines 288-290).

2. Figures 2D-E, 3G: MKRN2 localization mechanism requires clarification. The authors demonstrate that MKRN2 localization to SGs is dependent on active ubiquitination, as TAK243 treatment significantly reduces MKRN2 partitioning into SGs (Figure 2D-E). However, several mechanistic questions remain:

• Does MKRN2 localize to SGs through binding to ubiquitinated substrates within SGs, or does MKRN2 require its own ubiquitination activity to enter SGs?

• The observation that MKRN2 overexpression increases SG ubiquitin content (Figure 3G-H) could indicate either: (a) MKRN2 actively ubiquitinates substrates within SGs, or (b) MKRN2 recruitment brings along pre-ubiquitinated substrates from the cytoplasm.

• Is MKRN2 localization to SGs dependent on its E3 ligase activity? A catalytically inactive mutant of MKRN2 would help distinguish whether MKRN2 must actively ubiquitinate proteins to remain in SGs or whether it binds to ubiquitinated proteins independently of its catalytic activity. The authors should clarify whether MKRN2's SG localization depends on its catalytic activity or on binding to ubiquitinated proteins, as this would fundamentally affect the interpretation of its role in SG dynamics.

Authors: We thank the reviewer for these suggestions. A catalytically dead MKRN2-GFP was generated by mutating all His and Cys residues in the RING domain to Alanines. We detected a significant decrease in RING-MKRN2 partition to SGs relative to WT-MKRN2 (Fig S7C, S7D). These results strengthen the notion that MKRN2 localization to SGs depends on its E3 activity. It does not preclude SG association by other mechanisms, including via potential binding to "pre-ubiquitinated" substrates. To reflect this, the discussion now reads (lines 224-227): "Our experiments demonstrate that MKRN2 localization to SGs depends at least partially on its ubiquitination capacity, but recruitment via other mechanisms, such as its binding affinity for RNA or ubiquitinated proteins cannot be ruled out"

3. Figures 3-4: Discrepancy between assembly and disassembly phenotypes. MKRN2 knockdown produces distinct phenotypes during SG assembly versus disassembly. During assembly: smaller, more numerous SGs that fail to coalesce (Figure 3A-E), while during disassembly: delayed SG clearance (Figure 4A-D).

These phenotypes may reflect different roles for MKRN2 at different stages, but the mechanism underlying this stage-specificity is unclear:

• Does MKRN2 have different substrates or utilize different ubiquitin chain types during assembly versus disassembly?

Authors: In agreement with the Editor, upon transfer of the work from Review Commons to EMBO Reports, we feel that the experimental needs for determining MKRN2 specific substrates are not available in the lab and the time and resources required to create these falls beyond the scope of the current study.

• The increased SG liquidity upon MKRN2 depletion (Figure 3F) seems paradoxical with delayed disassembly- typically more liquid condensates disassemble faster. The authors interpret this as decreased coalescence into "dense and mature SGs," but this requires clarification.

• How does prevention of DRiP accumulation relate to the assembly defect? One would predict that DRiP accumulation would primarily affect disassembly (by reducing liquidity), yet MKRN2 depletion impacts both assembly dynamics and DRiP accumulation.

The authors should discuss how MKRN2's role in preventing DRiP accumulation mechanistically connects to both the assembly and disassembly phenotypes.

Authors: In the discussion we now clarify that smaller SGs in MKRN2 KD relate to maturation delay: "Consistent with our findings regarding VCP depletion ((Seguin et al. 2014)), we propose that the unregulated accumulation of DRiPs within early SG foci following MKRN2 depletion prevents their proper fusion and growth into normal, mature SGs. Accordingly, immature SGs are more diffusive and recover faster than mature SGs after photobleaching (Wheeler et al. 2016)." (lines 246-250).

4. Figure 5: Incomplete characterization of MKRN2 substrates. While the authors convincingly demonstrate that MKRN2 prevents DRiP accumulation in SGs (Figure 5C-D), the direct substrates of MKRN2 remain unknown. The authors acknowledge in the

limitations that "the direct MKRN2 substrates and ubiquitin-chain types (K63/K48) are currently unknown." However, several approaches could strengthen the mechanistic understanding:

• Do DRiPs represent direct MKRN2 substrates? Co-immunoprecipitation of MKRN2 followed by ubiquitin-chain specific antibodies (K48 vs K63) could reveal whether MKRN2 mediates degradative (K48) or non-degradative (K63) ubiquitination.

Authors: The DRiPs generated in the study represent truncated versions of all the proteins that were in the process of being synthesized by the cell at the moment of the stress, and therefore include both MKRN2 specific substrates and MKRN2 independent substrates. Identifying specific MKRN2 substrates, while interesting as a new research avenue, is not within the scope of the present study. In agreement with the Editor, upon transfer of the work from Review Commons to EMBO Reports, we feel that the experimental needs for determining MKRN2 specific substrates are not available in the lab and the time and resources required to create these falls beyond the scope of the current study.

• Given that VCP cofactors (such as UFD1L, PLAA) are depleted from SGs upon UBA1 inhibition (Figure 2C) and these cofactors recognize ubiquitinated substrates, does MKRN2 function upstream of VCP recruitment? Testing whether MKRN2 depletion affects VCP cofactor localization to SGs would clarify this pathway.

Authors: We thank the reviewer for the request, which we have addressed by performing MKRN2 KD, VCP immunofluorescence microscopy and SG partition coefficient analysis. We did not detect a significant decrease in VCP partitioning to SGs following MKRN2 KD (new Fig. S7E). Thus, in our hands the knockdown of MKRN2 is not sufficient to affect VCP partitioning to SGs.

• The authors note that MKRN2 knockdown produces a phenotype reminiscent of VCP inhibition-smaller, more numerous SGs with increased DRiP partitioning. This similarity suggests MKRN2 may function in the same pathway as VCP. Direct epistasis experiments would strengthen this connection.

Authors: In agreement with the editor, upon transfer of the work from Review Commons to EMBO Reports, we interpreted the request to perform this study as a conditional request that depends on detecting altered VCP partitioning to SGs upon MKRN2 knockdown. Since these results were negative in nature in our hands, we did not proceed to performing a double MKRN2/VCP KD experiment.

5. Alternative explanations for the phenotype of delayed disassembly with TAK243 or MKRN2 depletion- the authors attribute this to DRiP accumulation, but TAK243 affects global ubiquitination. Could impaired degradation of other SG proteins (not just DRiPs) contribute to delayed disassembly? Does proteasome inhibition (MG-132 treatment) phenocopy the MKRN2 depletion phenotype? This would support that MKRN2-mediated proteasomal degradation (via K48 ubiquitin chains) is key to the phenotype.

Authors: We thank the reviewer for this great question. TAK243 represents a much broader perturbation than MKRN2 knockdown, likely involving additional mechanisms in delayed SG disassembly. Specifically, G3BP1 ubiquitination by TRIM21 regulates disassembly (Gwon et al. 2021; Yang et al. 2023) and probably contributes to the TAK243 phenotype. We demonstrate that MKRN2 knockdown does not increase G3BP1 expression (Fig. S5A), suggesting MKRN2 does not mediate G3BP1 degradation. This more widespread impact explains why TAK243 affects SG disassembly more significantly than MKRN2 depletion. We now write in the Discussion: “We note that the SG disassembly phenotype upon MKRN2 knockdown is less pronounced than that observed with TAK243 treatment.”; lines 275-276).

6. Comparison with other E3 ligases (Supplementary Figure 5):

The authors show that CNOT4 and ZNF598 depletion also affect SG dynamics, though to lesser extents than MKRN2. However:

• Do these E3 ligases also prevent DRiP accumulation in SGs? Testing OP-puro partitioning in CNOT4- or ZNF598-depleted cells would reveal whether DRiP clearance is a general feature of SG-localized E3 ligases or specific to MKRN2.

• Are there redundant or compensatory relationships between these E3 ligases? Do double knockdowns have additive effects?

Authors: Our paper presents a study of the E3 ligase MKRN2. Generalizing these observations to ZNF598, CNOT4 and perhaps an even longer list of E3s, may be an interesting question, yet, in agreement with the Editor, upon transfer of the work from Review Commons to EMBO Reports, it remains outside the scope of this manuscript.

• The authors note that MKRN2 is "the most highly SG-depleted E3 upon TAK243 treatment"-does this mean MKRN2 has the strongest dependence on active ubiquitination for its SG localization, or simply that it has the highest basal level of SG partitioning?

Authors: We thank the reviewer for this smart question. MKRN2 has the strongest dependence on active ubiquitination as we now clarify better in the Results (lines 115-120): “Importantly,

examination of the basal enrichment level of each of these E3 ligases in the APEX data showed that MKRN2 was not the E3 with the highest level of basal SG enrichment (MKRN2=~1.37 Log2FC, ZNF598=~1.5 Log2FC, CNOT4=0.58 Log2FC, TRIM25=0.52 Log2FC and TRIM26=0.58 Log2FC; Table S3), supporting the interpretation that MKRN2's localization to SG is dependent on UBA1 activity and not simply due to a generally higher level of SG localization."

Reviewer #2 (Significance (Required)):

This is a well-executed study that identifies MKRN2 as an important regulator of stress granule dynamics and proteostasis. The combination of proximity proteomics, live imaging, and functional assays provides strong evidence for MKRN2's role in preventing DRiP accumulation and maintaining granulostasis. However, key mechanistic questions remain, particularly regarding MKRN2's direct substrates, the ubiquitin chain types it generates, and how its enzymatic activity specifically prevents DRiP accumulation while promoting both SG coalescence and disassembly. Addressing the suggested revisions, particularly those related to MKRN2's mechanism of SG localization and substrate specificity, would significantly strengthen the manuscript and provide clearer insights into how ubiquitination maintains the dynamic properties of stress granules under proteotoxic stress.

Authors: We would like to thank reviewer #2 for their thorough and overall favorable review.

Reviewer #3 (Evidence, reproducibility and clarity (Required)):

In this paper, Amzallag et al. investigate the relationship between ubiquitination and the dynamics of stress granules (SGs). They utilize proximity ligation coupled mass spectrometry to identify SG components under conditions where the proteasome is inhibited by a small drug that targets UBiquitin-like modifier Activating enzyme 1 (UBA1), which is crucial for the initial step in the ubiquitination of misfolded proteins. Their findings reveal that the E3 ligase Makorin2 (MKRN2) is a novel component of SGs. Additionally, their data suggest that MKRN2 is necessary for processing damaged ribosome-associated proteins (DRIPs) during heat shock (HS). In the absence of MKRN2, DRIPs accumulate in SGs, which affects their dynamics.

Major comments:

Assess the knockdown efficiency (KD) for CNOT1, ZNF598, and MKRN2 to determine if the significant effect observed on SG dynamics upon MKRN2 depletion is due to the protein's function rather than any possible differences in KD efficiency.

Authors: We thank the reviewer for the question. To address potential variability in knockdown efficiency, we have quantified CNOT4, ZNF598, and MKRN2 mRNA levels by RT-qPCR following siRNA knockdown (Fig S4C, S4D, S4E). KD efficiency was ~90% for all genes.

Since HS-induced stress granules (SGs) are influenced by the presence of TAK-243 or MKRN2 depletion, could it be that these granules become more mature and thus acquire more defective ribosomal products (DRIPs)? Do HS cells reach the same level of DRIPs, as assessed by OP-Puro staining, at a later time point?

Authors: We thank the reviewer for the question. The study of Mateju et al. carefully characterized the time course of DRiP accumulation in stress granules during heat shock, and revealed it is decreasing after the 90 minutes point (Appendix Figure S7; 10.15252/embj.201695957). We therefore interpret DRiP accumulation after 90 minutes in stress granules as a pathological state, reflecting Ubiquitin (and TAK243) dependent degradation of DRiPs, rather than a normal, more “mature” stress granule state.

Incorporating OP-Puro can lead to premature translation termination, potentially confounding results. Consider treating cells with a short pulse (i.e., 5 minutes) of OP-Puro just before fixation.

Authors: Thank you for this suggestion. Treating the cell with a short pulse of OP-Puro just before fixation will lead to the labelling of a small amount of proteins, which are likely undetectable using conventional microscopy or Western blotting. Furthermore, it will lead to the unwanted labeling of stress-responsive proteins that are translated with non canonical cap-independent mechanisms upon stress.

Is MKRN2's dependence limited to HS-induced SGs?

Authors: We thank the reviewer for this important additional question. We have performed live imaging of SG assembly and disassembly under sodium arsenite (oxidative) stress following MKRN2 KD. No effect on SG disassembly could be detected, suggesting that MKRN2's effect on SGs may be limited to HS SGs.

Minor comments:

Abstract:

Introduce UBA1.

Introduction:

The reference [2] should be replaced with 25719440.

Results:

Line 70, 'G3BP1 and 2 genes,' is somewhat misleading. Consider rephrasing into 'G3BP1 and G3BP2 genes'.

Line 103: considers rephrasing 'we orthogonally validated the ubiquitin-dependent interaction' to 'we orthogonally validated the ubiquitin-dependent stress granule localization'.

Line 125: '(fig.3C, EI Supplementary fig. 3)' Remove 'I'.

Methods:

line 260: the reference is not linked (it should be ref. [26]).

Line 225: Are all the KDs being performed using the same method? Please specify.

Authors: The text has been altered to reflect the reviewer's suggestions in the section "minor comments".

Fig.2C: Consider adding 'DEPLETED' on top of the scheme.

Reviewer #3 (Significance (Required)):

The study offers valuable insights into the degradative processes associated with SGs. The figures are clear, and the experimental quality is high. The authors do not overstate or overinterpret their findings, and the results effectively support their claims. However, the study lacks orthogonal methods to validate the findings and enhance the results. For instance, incorporating biochemical and reporter-based methods to measure degradation-related intermediate products (DRIPs) would be beneficial. Additionally, utilizing multiple methods to block ubiquitination, studying the dynamics of MKRN2 on SGs, and examining the consequences of excessive DRIPs on the cell fitness of SGs would further strengthen the research.

Authors: We would like to thank reviewer #3 for their time, feedback and overall favorable review.

Ganassi, Massimo, Daniel Mateju, Ilaria Bigi, et al. 2016. “A Surveillance Function of the HSPB8-BAG3-HSP70 Chaperone Complex Ensures Stress Granule Integrity and Dynamism.” *Molecular Cell* 63 (5): 796–810.

Gwon, Youngdae, Brian A. Maxwell, Regina-Maria Kolaitis, Peipei Zhang, Hong Joo Kim, and J. Paul Taylor. 2021. “Ubiquitination of G3BP1 Mediates Stress Granule Disassembly in a Context-Specific Manner.” *Science* 372 (6549): eabf6548.

Markmiller, Sebastian, Sahar Soltanieh, Kari L. Server, et al. 2018. “Context-Dependent and Disease-Specific Diversity in Protein Interactions within Stress Granules.” *Cell* 172 (3): 590–604.e13.

Seguin, S. J., F. F. Morelli, J. Vinet, et al. 2014. “Inhibition of Autophagy, Lysosome and VCP Function Impairs Stress Granule Assembly.” *Cell Death and Differentiation* 21 (12): 1838–1851.

Shin, Chanyoung, Yuma Ito, Shota Ichikawa, Makio Tokunaga, Kumiko Sakata-Sogawa, and Takashi Tanaka. 2017. “MKRN2 Is a Novel Ubiquitin E3 Ligase for the p65 Subunit of NF-κB and Negatively Regulates Inflammatory Responses.” *Scientific Reports* 7 (1): 46097.

Wheeler, Joshua R., Tyler Matheny, Saumya Jain, Robert Abrisch, and Roy Parker. 2016. “Distinct Stages in Stress Granule Assembly and Disassembly.” *eLife* 5 (September). https://doi.org/10.7554/elife.18413.

Wolf, Eric J., Amanda Miles, Eliza S. Lee, et al. 2020. “MKRN2 Physically Interacts with GLE1 to Regulate mRNA Export and Zebrafish Retinal Development.” *Cell Reports* 31 (8): 107693.

Yang, Cuiwei, Zhangshun Wang, Yingjin Kang, et al. 2023. “Stress Granule Homeostasis Is Modulated by TRIM21-Mediated Ubiquitination of G3BP1 and Autophagy-Dependent Elimination of Stress Granules.” *Autophagy* 19 (7): 1934–1951.

Youn, Ji-Young, Wade H. Dunham, Seo Jung Hong, et al. 2018. “High-Density Proximity Mapping Reveals the Subcellular Organization of mRNA-Associated Granules and Bodies.” *Molecular Cell* 69 (3): 517–532.e11.

Dear Prof. Hornstein

Thank you for the submission of your revised manuscript to EMBO reports. We have now received the full set of referee reports that is copied below.

As you will see, both referees are very positive about the study and request only minor changes to clarify text and figures.

From the editorial side, there are also a few things that we need before we can proceed with the official acceptance of your study.

- Your manuscript will be published in our Reports section, which requires the combination of the Results and Discussion section.

- The Conflict of Interests statement needs to be renamed to Disclosure and Competing Interests Statement and placed after the Acknowledgments.

- Regarding the Author Contributions, we use CRediT to specify the contributions of each author in the journal submission system. Therefore, please remove the Author Contributions from the manuscript file and make sure that the author contributions in our online manuscript tracking system are correct and up-to-date. The information you specified in the system will be automatically retrieved and typeset into the article. You can enter additional information in the free text box provided, if you wish. See also our guide to authors https://www.embopress.org/page/journal/14693178/authorguide#authorshipguidelines.

- References: et al needs to be used after 10 author names. We further noticed a few DOIs which should only be used for preprints and datasets that have not been published yet.

- The funding information in the manuscript and the online system must be congruent. We noticed these are not in the system (but perhaps do not need to be there?): the Department of Defense - Congressionally Directed Medical Research Program, Longitude Prize on ALS by Motor Neurone Disease Association (MNDA), Federal German Ministry for Education and Research. In the text all funding information needs to be part of the Acknowledgements, the funding title should be removed.

- Please add a callout for Fig. 1E in the text.

- The EV tables (except for Table EV5) are actually datasets and need to be updated to Dataset EV1-Dataset EV4 in all places (source files, titles in the system, legends, manuscript callouts); each Excel file should be unzipped and uploaded as a separate file. Table EV5 (primers) would then be Table EV1.

- Appendix: the table of content needs page numbers for each item.

- Appendix Figure S2: strictly spoken you measured and quantified 2 repeats, therefore a statistical analysis seems not supported by the low 'n' number.

- Appendix Figure S3 has a typo in the legend: Ant-ubiquitin should be Anti-ubiquitin.

- Appendix Figure S4A: the graph's legend is cut-off and not fully visible.

- Appendix Figure legends in general: In some of the legends the panel names (A., B. etc) are not bolded and therefore hard to identify.

- We only need the Reagents and Tools table that you uploaded as separate file. Please remove the one in the methods section.

- Figure legends should be placed at the end of the manuscript, after the References.

- Line 414 (methods section): please replace the PMID with the citation.

- Please provide the specific URL for PXD076622 datasets in the data availability statement.

- Please address the following points in the figure legends and please mark the changes (color or track changes):
A) Please provide the exact p values in the legends of figures 1B, C, D, F; 2E, 3B, C, D, E, F, H; 4B-D; 5B, D (unless $p<0.0001$).
B) Please indicate the statistical test used for data analysis in the legends of figures 2A, B
C) Please note that information related to n is missing in the legends of figures 2A, B
D) Please note that the measure of center for the error bars needs to be defined in the legends of figures 4B, 5B, D

- Source data for Figure 2: you can remove the empty folder "Fig. 2A-2C" from the folder.

- We perform a routine check on all source data .xls files. The program detected duplications in some of them. I have not digged into them, but could you please check the color-coded files and assure that all duplicated numbers are correct and justified? Thank you.

- A spot check on the raw data for Figure 1A showed that the images showing stress granules have been contrast modified to quite some extent. Please check whether this is really needed to visualize the speckles.

- As a standard procedure, we edit the title and abstract of manuscripts to make them more accessible for our general readership. Please find my suggestions below my signature for your approval.

- Finally, EMBO Reports papers are accompanied online by
A) a short (1-2 sentences) summary of the findings and their significance,
B) 2-3 bullet points highlighting key results and
C) a schematic summary figure that provides a sketch of the major findings (not a data image).
Please provide the summary figure as a separate file in PNG or JPG format at a size of 550x300 pixels (width x height). Please note that the size is rather small and that text needs to be readable at the final size. Please send us this information along with the revised manuscript.

We look forward to seeing a final version of your manuscript as soon as possible.

With kind regards,

Martina

Martina Rembold, PhD
Senior Editor
EMBO reports

==================

Referee #1:

The authors have thoughtfully addressed my previous comments. I particularly appreciate the new catalytic-dead RING mutant experiment (Fig. S7C-D), which supports the conclusion that MKRN2 SG localization depends at least partially on its ligase activity, and the additional clarifications on basal vs. UBA1-dependent SG enrichment, the assembly/disassembly mechanistic connection, and the VCP partitioning test. The appropriately tempered language regarding MKRN2 substrates and chain specificity is reasonable given the agreed scope.

Referee #2:

I have reviewed the revised version of the manuscript submitted by the authors. Overall, the authors have addressed the main concerns raised in my previous review, either through additional experimental validation or by clearly stating certain assumptions as preliminary and hypothetical. Specifically, they performed experiments to clarify the endogenous localization of MKRN2, demonstrated effective knockdown of MKRN2, and assessed the impact of MKRN2 depletion on stress granule (SG) marker proteins. However, I remain somewhat confused at one point. In response to my request to generate a catalytically inactive mutant, the authors indicated that this would be time-consuming and beyond the scope of the study. Yet, in response to Reviewer 2, they generated a RING-dead catalytic mutant to address a similar concern. It is therefore unclear why my initial request was considered out of scope if a comparable experiment was ultimately performed. In general, the manuscript has improved in readability. The experiments are now more clearly presented, and the conclusions are better supported. Where results remain inconclusive, the authors appropriately acknowledge this and suggest avenues for future investigation, particularly regarding the involvement of additional ubiquitination factors in SG dynamics and DRIP prevention. I recommend the manuscript for publication. As a minor suggestion, the authors could strengthen the introduction or discussion by adding a few lines on the potential impact of impaired SG disassembly in pathological conditions.

=====================

Suggested title and abstract:

The E3 ligase MKRN2 prevents DRiP accumulation in stress granules and maintains granulostasis

Stress granules (SGs) are transient cytoplasmic biomolecular condensates that play a role in the cellular response to proteotoxic stress. It has been previously shown that ubiquitination regulates SG dynamics; however, the specific mechanisms by which ubiquitin affects SGs are not fully understood. Here, using proximity proteomics, we discover that the recruitment of several E3 ubiquitin ligases and ubiquitin hydrolases to SGs is dependent on the activity of the E1 ubiquitin ligase UBA1. The RNA-binding E3 ubiquitin-protein ligase Makorin 2 (MKRN2) is strongly depleted from SGs in the absence of UBA1 function. MKRN2 promotes both the proper formation of SGs and their disassembly following stress recovery, by preventing the accumulation of misfolding-prone defective ribosomal products (DRiPs) within SGs. Therefore, MKRN2 is a novel regulator of SGs that mediates the maintenance of granulostasis.

Referee #1:

The authors have thoughtfully addressed my previous comments. I particularly appreciate the new catalytic-dead RING mutant experiment (Fig. S7C-D), which supports the conclusion that MKRN2 SG localization depends at least partially on its ligase activity, and the additional clarifications on basal vs. UBA1-dependent SG enrichment, the assembly/disassembly mechanistic connection, and the VCP partitioning test. The appropriately tempered language regarding MKRN2 substrates and chain specificity is reasonable given the agreed scope.

Referee #2:

I have reviewed the revised version of the manuscript submitted by the authors. Overall, the authors have addressed the main concerns raised in my previous review, either through additional experimental validation or by clearly stating certain assumptions as preliminary and hypothetical. Specifically, they performed experiments to clarify the endogenous localization of MKRN2, demonstrated effective knockdown of MKRN2, and assessed the impact of MKRN2 depletion on stress granule (SG) marker proteins. However, I remain somewhat confused at one point. In response to my request to generate a catalytically inactive mutant, the authors indicated that this would be time-consuming and beyond the scope of the study. Yet, in response to Reviewer 2, they generated a RING-dead catalytic mutant to address a similar concern. It is therefore unclear why my initial request was considered out of scope if a comparable experiment was ultimately performed. In general, the manuscript has improved in readability. The experiments are now more clearly presented, and the conclusions are better supported. Where results remain inconclusive, the authors appropriately acknowledge this and suggest avenues for future investigation, particularly regarding the involvement of additional ubiquitination factors in SG dynamics and DRIP prevention. I recommend the manuscript for publication. As a minor suggestion, the authors could strengthen the introduction or discussion by adding a few lines on the potential impact of impaired SG disassembly in pathological conditions.

***

Rev_Com_number: RC-2025-03277
New_manu_number: EMBOR-2026-63634V2
Corr_author: Hornstein
Title: Ubiquitination regulates granulostasis and DRiP accumulation in SGs under heat stress via the E3 ligase MKRN2

Dear Martina,

We have completed the requested corrections to our manuscript and uploaded the necessary files to the system. Please find a point by point reply to your requests below:

From the editorial side, there are also a few things that we need before we can proceed with the official acceptance of your study.

- Your manuscript will be published in our Reports section, which requires the combination of the Results and Discussion section.

We have combined the results and discussion into a single section entitled "Results and Discussion".

- The Conflict of Interests statement needs to be renamed to Disclosure and Competing Interests Statement and placed after the Acknowledgments.

We have appropriately renamed and repositioned this section.

- Regarding the Author Contributions, we use CRediT to specify the contributions of each author in the journal submission system. Therefore, please remove the Author Contributions from the manuscript file and make sure that the author contributions in our online manuscript tracking system are correct and up-to-date. The information you specified in the system will be automatically retrieved and typeset into the article. You can enter additional information in the free text box provided, if you wish. See also our guide to authors https://www.embopress.org/page/journal/14693178/authorguide#authorshipguide lines.

We have removed the Author contributions section from the manuscript text and ensured that the information in the submission system is accurate.

- References: et al needs to be used after 10 author names. We further noticed a few DOIs which should only be used for preprints and datasets that have not been published yet.

This was done using the dedicated EMBO Reports citation style in Paperpile.

- The funding information in the manuscript and the online system must be congruent. We noticed these are not in the system (but perhaps do not need to be there?): the Department of Defense - Congressionally Directed Medical Research Program, Longitude Prize on ALS by Motor Neurone Disease Association (MNDA), Federal German Ministry for Education and Research. In the text all funding information needs to be part of the Acknowledgements, the funding title should be removed.

We have added these missing funding sources into the online submission system.

- Please add a callout for Fig. 1E in the text.

We have added a callout for Fig. 1E at line 98 of the manuscript.

- The EV tables (except for Table EV5) are actually datasets and need to be updated to Dataset EV1-Dataset EV4 in all places (source files, titles in the system, legends, manuscript callouts); each Excel file should be unzipped and uploaded as a separate file. Table EV5 (primers) would then be Table EV1.

We have renamed the files appropriately, referred to them correctly in the text and have replaced the files submitted in the online submission system.

- Appendix: the table of content needs page numbers for each item.

Page numbers were added to the entire Appendix and have been added to the table of content.

- Appendix Figure S2: strictly spoken you measured and quantified 2 repeats, therefore a statistical analysis seems not supported by the low 'n' number.

Thank you for raising this and for the opportunity to clarify: Each graph in Appendix Figure S2 represents a single, independent FRAP experiment with 15–20 individual stress granules (reported as mean ± s.e.m. per time point), analyzed via two-way repeated measures ANOVA.
To ensure reproducibility, **all conditions were performed in three independent experimental repeats (n = 3)**, which are distributed across the manuscript as follows:
For the UBA1 Inhibitor (TAK243) FRAP (3 total repeats): Two independent repeats are shown in Appendix Figure S2A, and the third repeat is presented in main Figure 1F. Statistics are done on each individual repeat.
For the MKRN2 KD FRAP (3 total repeats): Two independent repeats are shown in Appendix Figure S2B, and the third repeat is presented in main Figure 3F. Statistics are done on each individual repeat.

- Appendix Figure S3 has a typo in the legend: Ant-ubiquitin should be Anti-ubiquitin.

This typo has been fixed.

- Appendix Figure S4A: the graph's legend is cut-off and not fully visible.

The graph’s legend in Appendix Fig. S4A was made clearer.

- Appendix Figure legends in general: In some of the legends the panel names (A., B. etc) are not bolded and therefore hard to identify.

All panel names have been bolded.

- We only need the Reagents and Tools table that you uploaded as separate file. Please remove the one in the methods section.

The Reagents and Tools table was removed.

- Figure legends should be placed at the end of the manuscript, after the References.

We have moved the main figure legends to the very end of the manuscript.

- Line 414 (methods section): please replace the PMID with the citation.

We have cited the study appropriately.

- Please provide the specific URL for PXD076622 datasets in the data availability statement.

As discussed via email, we have added the following statement in the Data Availability section until our manuscript has a DOI: "The mass spectrometry proteomics data have been deposited to the ProteomeXchange Consortium via the PRIDE partner repository with the dataset identifier PXD076622."

- Please address the following points in the figure legends and please mark the changes (color or track changes):
A) Please provide the exact p values in the legends of figures 1B, C, D, F; 2E, 3B, C, D, E, F, H; 4B-D; 5B, D (unless p<0.0001).
B) Please indicate the statistical test used for data analysis in the legends of figures 2A, B
C) Please note that information related to n is missing in the legends of figures 2A, B
D) Please note that the measure of center for the error bars needs to be defined in the legends of figures 4B, 5B, D

All requested edits to the main figure legends were done.

- Source data for Figure 2: you can remove the empty folder "Fig. 2A-2C" from the folder.

We have removed this folder in the online submission system.

- We perform a routine check on all source data .xls files. The program detected duplications in some of them. I have not digged into them, but could you please check the color-coded files and assure that all duplicated numbers are correct and justified? Thank you.

This issue was resolved via email.

- A spot check on the raw data for Figure 1A showed that the images showing stress granules have been contrast modified to quite some extent. Please check whether this is really needed to visualize the speckles.

Thank you for raising this point. Contrast modifications in Figure 1A and throughout the manuscript were applied solely to assist the reader in visualizing stress granules. Because raw fluorescence signals can be difficult to discern on standard screens or in print, adjustments are necessary for effective visualization. This is particularly critical for live imaging, where low exposure time and intensity are maintained to prevent phototoxicity during long imaging runs. Importantly, all brightness and contrast adjustments were applied uniformly across all comparative conditions within each figure, and the visual representations accurately reflect the quantitative data. Specific values used for contrast adjustments remain embedded within the metadata of the provided .czi image files.

- As a standard procedure, we edit the title and abstract of manuscripts to make them more accessible for our general readership. Please find my suggestions below my signature for your approval.

Thank you for your suggestions for improving our title and abstract. We have changed the title according to your suggestion. We have also changed the abstract according to your suggestion, with one small alteration shown as a tracked change in the manuscript file.

- Finally, EMBO Reports papers are accompanied online by
A) a short (1-2 sentences) summary of the findings and their significance,
B) 2-3 bullet points highlighting key results and
C) a schematic summary figure that provides a sketch of the major findings (not a data image).
Please provide the summary figure as a separate file in PNG or JPG format at a size of 550x300 pixels (width x height). Please note that the size is rather small and that text needs to be readable at the final size. Please send us this information along with the revised manuscript.

A word document containing our short summary and bullet points was uploaded to the system. A schematic summary was also sent.

Looking forward to hearing from you,
Emmanuel.

Emmanuel Amzallag, PhD student
Eran Hornstein Lab, Department of Molecular Genetics
Weizmann Institute of Science
234 Herzl St. Rehovot 7610001
Israel

Prof. Eran Hornstein
Weizmann Institute of Science
Molecular Genetics
1314 Mayer building
Rehovot 76100
Israel

Dear Eran,

Thank you for implementing the final minor changes and adjustments. I am very pleased to accept your manuscript for publication in the next available issue of EMBO reports. Thank you for your contribution to our journal.

Your manuscript will be processed for publication by EMBO Press. It will be copy edited and you will receive page proofs prior to publication. Please note that you will be contacted by Springer Nature Author Services to complete licensing and payment information.

You may qualify for financial assistance for your publication charges - either via a Springer Nature fully open access agreement or an EMBO initiative. Check your eligibility: https://link.springer.com/journal/44319/how-to-publish-with-us

Should you be planning a Press Release on your article, please notify the production team via this form in order to coordinate publication and release dates: https://forms.cloud.microsoft/e/eH4A8JVP6g

If you have any questions, please do not hesitate to contact the Editorial Office. Thank you for your contribution to EMBO Reports.

Kind regards,

Martina

Martina Rembold, PhD
Senior Editor
EMBO reports

>>> Please note that it is EMBO Reports policy for the transcript of the editorial process (containing referee reports and your response letter) to be published as an online supplement to each paper. If you do NOT want this, you will need to inform the Editorial Office via email immediately. More information is available here: https://link.springer.com/partners/embo-press/editorial-policies#Peer%20review

***
Rev_Com_number: RC-2025-03277
New_manu_number: EMBOR-2026-63634V3
Corr_author: Hornstein
Title: The E3 ligase MKRN2 prevents DRiP accumulation in stress granules and maintains granulostasis

EMBO Press Author Checklist

Corresponding Author Name: Eran Hornstein	
Journal Submitted to: EMBO Reports	
Manuscript Number: EMBOR-2026-63634V1-T	

USEFUL LINKS FOR COMPLETING THIS FORM
The EMBO Journal - Author Guidelines
EMBO Reports - Author Guidelines
Molecular Systems Biology - Author Guidelines
EMBO Molecular Medicine - Author Guidelines

Reporting Checklist for Life Science Articles (updated January
This checklist is adapted from Materials Design Analysis Reporting (MDAR) Checklist for Authors. MDAR establishes a minimum set of requirements in transparent reporting in the life sciences (see Statement of Task: 10.31222/osf.io/9sm4x). Please follow the journal's guidelines in preparing your
Please note that a copy of this checklist will be published alongside your article.

Abridged guidelines for figures
1. Data
The data shown in figures should satisfy the following conditions:
- the data were obtained and processed according to the field's best practice and are presented to reflect the results of the experiments in an accurate and unbiased manner.
- ideally, figure panels should include only measurements that are directly comparable to each other and obtained with the same assay.
- plots include clearly labeled error bars for independent experiments and sample sizes. Unless justified, error bars should not be shown for technical
- if n<5, the individual data points from each experiment should be plotted. Any statistical test employed should be justified.
- Source Data should be included to report the data underlying figures according to the guidelines set out in the authorship guidelines on Data

2. Captions
Each figure caption should contain the following information, for each panel where they are relevant:
- a specification of the experimental system investigated (eg cell line, species name).
- the assay(s) and method(s) used to carry out the reported observations and measurements.
- an explicit mention of the biological and chemical entity(ies) that are being measured.
- an explicit mention of the biological and chemical entity(ies) that are altered/varied/perturbed in a controlled manner.
- the exact sample size (n) for each experimental group/condition, given as a number, not a range;
- a description of the sample collection allowing the reader to understand whether the samples represent technical or biological replicates (including how many animals, litters, cultures, etc.).
- a statement of how many times the experiment shown was independently replicated in the laboratory.
- definitions of statistical methods and measures:
  - common tests, such as t-test (please specify whether paired vs. unpaired), simple χ2 tests, Wilcoxon and Mann-Whitney tests, can be unambiguously identified by name only, but more complex techniques should be described in the methods section;
  - are tests one-sided or two-sided?
  - are there adjustments for multiple comparisons?
  - exact statistical test results, e.g., P values = x but not P values < x;
  - definition of 'center values' as median or average;
  - definition of error bars as s.d. or s.e.m.

Please complete ALL of the questions below.
Select "Not Applicable" only when the requested information is not relevant for your study.

Materials

Newly Created Materials	Information included in the manuscript?	In which section is the information available? (Reagents and Tools Table, Materials and Methods, Figures, Data Availability Section)
New materials and reagents need to be available; do any restrictions apply?	Not Applicable	

Antibodies	Information included in the manuscript?	In which section is the information available? (Reagents and Tools Table, Materials and Methods, Figures, Data Availability Section)
For **antibodies** provide the following information:<br>- Commercial antibodies: RRID (if possible) or supplier name, catalogue number and or/clone number<br>- Non-commercial: RRID or citation	Yes	Reagents and Tools Table; Materials and Methods

DNA and RNA sequences	Information included in the manuscript?	In which section is the information available? (Reagents and Tools Table, Materials and Methods, Figures, Data Availability Section)
**Short novel DNA or RNA including primers, probes**: provide the sequences.	Yes	Reagents and Tools Table; Table EV5

Cell materials	Information included in the manuscript?	In which section is the information available? (Reagents and Tools Table, Materials and Methods, Figures, Data Availability Section)
**Cell lines:** Provide species information, strain. Provide accession number in repository **OR** supplier name, catalog number, clone number, and/**OR** RRID.	Yes	Reagents and Tools Table; Materials and Methods
**Primary cultures:** Provide species, strain, sex of origin, genetic modification status.	Not Applicable	
Report if the cell lines were recently **authenticated** (e.g., by STR profiling) and tested for mycoplasma contamination.	Yes	Materials and Methods

Experimental animals	Information included in the manuscript?	In which section is the information available? (Reagents and Tools Table, Materials and Methods, Figures, Data Availability Section)
**Laboratory animals or Model organisms:** Provide species, strain, sex, age, genetic modification status. Provide accession number in repository **OR** supplier name, catalog number, clone number, **OR** RRID.	Not Applicable	
**Animal observed in or captured from the field:** Provide species, sex, and age where possible.	Not Applicable	
Please detail **housing and husbandry conditions**.	Not Applicable	

Plants and microbes	Information included in the manuscript?	In which section is the information available? (Reagents and Tools Table, Materials and Methods, Figures, Data Availability Section)
**Plants:** provide species and strain, ecotype and cultivar where relevant, unique accession number if available, and source (including location for collected wild specimens).	Not Applicable	
**Microbes:** provide species and strain, unique accession number if available, and source.	Not Applicable	

Human research participants	Information included in the manuscript?	In which section is the information available? (Reagents and Tools Table, Materials and Methods, Figures, Data Availability Section)
If collected and within the bounds of privacy constraints report on age, sex and gender or ethnicity for all study participants.	Not Applicable	

Core facilities	Information included in the manuscript?	In which section is the information available? (Reagents and Tools Table, Materials and Methods, Figures, Data Availability Section)
If your work benefited from core facilities, was their service mentioned in the acknowledgments section?	Yes	Acknowledgments

Design

**Study protocol**	**Information included in the manuscript?**	**In which section is the information available?** (Reagents and Tools Table, Materials and Methods, Figures, Data Availability Section)
If study protocol has been **pre-registered, provide DOI in the manuscript**. For clinical trials, provide the trial registration number **OR** cite DOI.	Not Applicable	
Report the **clinical trial registration number** (at ClinicalTrials.gov or equivalent), where applicable.	Not Applicable	

**Laboratory protocol**	**Information included in the manuscript?**	**In which section is the information available?** (Reagents and Tools Table, Materials and Methods, Figures, Data Availability Section)
Provide DOI OR other citation details if **external detailed step-by-step protocols** are available.	Not Applicable	

**Experimental study design and statistics**	**Information included in the manuscript?**	**In which section is the information available?** (Reagents and Tools Table, Materials and Methods, Figures, Data Availability Section)
Include a statement about **sample size** estimate even if no statistical methods were used.	Yes	Materials and Methods
Were any steps taken to minimize the effects of subjective bias when allocating animals/samples to treatment (e.g. **randomization procedure**)? If yes, have they been described?	Not Applicable	
Include a statement about **blinding** even if no blinding was done.	Yes	Materials and Methods
Describe **inclusion/exclusion criteria** if samples or animals were excluded from the analysis. Were the criteria pre-established?<br><br>If sample or data points were omitted from analysis, report if this was due to attrition or intentional exclusion and provide justification.	Not Applicable	
For every figure, are **statistical tests** justified as appropriate? Do the data meet the assumptions of the tests (e.g., normal distribution)? Describe any methods used to assess it. Is there an estimate of variation within each group of data? **I**s the variance similar between the groups that are being statistically compared?	Yes	Materials and Methods; Figure legends

**Sample definition and in-laboratory replication**	**Information included in the manuscript?**	**In which section is the information available?** (Reagents and Tools Table, Materials and Methods, Figures, Data Availability Section)
In the figure legends: state number of times the experiment was **replicated** in laboratory.	Yes	Figure legends
In the figure legends: define whether data describe **technical or biological replicates**.	Yes	Figure legends

Ethics

**Ethics**	**Information included in the manuscript?**	**In which section is the information available?** (Reagents and Tools Table, Materials and Methods, Figures, Data Availability Section)
Studies involving **human participants**: State details of **authority granting ethics approval** (IRB or equivalent committee(s), provide reference number for approval.	Not Applicable	
Studies involving **human participants**: Include a statement confirming that **informed consent** was obtained from all subjects and that the experiments conformed to the principles set out in the WMA Declaration of Helsinki and the Department of Health and Human Services Belmont Report.	Not Applicable	
Studies involving **human participants:** For publication of **patient photos**, include a statement confirming that consent to publish was obtained.	Not Applicable	
Studies involving experimental **animals**: State details of **authority granting ethics approval** (IRB or equivalent committee(s), provide reference number for approval. Include a statement of compliance with ethical regulations.	Not Applicable	
Studies involving **specimen and field samples:** State if relevant **permits** obtained, provide details of authority approving study; if none were required, explain why.	Not Applicable	

**Dual Use Research of Concern (DURC)**	**Information included in the manuscript?**	**In which section is the information available?** (Reagents and Tools Table, Materials and Methods, Figures, Data Availability Section)
Could your study fall under dual use research restrictions? Please check biosecurity documents and list of **select agents and toxins** (CDC): https://www.selectagents.gov/sat/list.htm	Not Applicable	
If you used a select agent, is the security level of the lab appropriate and reported in the manuscript?	Not Applicable	
If a study is subject to dual use research of concern regulations, is the name of the **authority granting approval and reference number** for the regulatory approval provided in the manuscript?	Not Applicable	

Reporting

The MDAR framework recommends adoption of discipline-specific guidelines, established and endorsed through community initiatives. Journals have their own policy about requiring specific guidelines and recommendations to complement MDAR.

**Adherence to community standards**	**Information included in the manuscript?**	**In which section is the information available?** (Reagents and Tools Table, Materials and Methods, Figures, Data Availability Section)
State if relevant guidelines or checklists (e.g., **ICMJE, MIBBI, ARRIVE, PRISMA**) have been followed or provided.	Not Applicable	
For **tumor marker prognostic studies,** we recommend that you follow the **REMARK** reporting guidelines (see link list at top right). See author guidelines, under 'Reporting Guidelines'. Please confirm you have followed these guidelines.	Not Applicable	
For **phase II and III randomized controlled trials**, please refer to the **CONSORT** flow diagram (see link list at top right) and submit the CONSORT checklist (see link list at top right) with your submission. See author guidelines, under 'Reporting Guidelines'. Please confirm you have submitted this list.	Not Applicable	

Data Availability

**Data availability**	**Information included in the manuscript?**	**In which section is the information available?** (Reagents and Tools Table, Materials and Methods, Figures, Data Availability Section)
Have **primary datasets** been deposited according to the journal's guidelines (see 'Data Deposition' section) and the respective accession numbers provided in the Data Availability Section?	Yes	Data Availability Section
Were **human clinical and genomic datasets** deposited in a public access-controlled repository in accordance to ethical obligations to the patients and to the applicable consent agreement?	Not Applicable	
Are **computational models** that are central and integral to a study available without restrictions in a machine-readable form? Were the relevant accession numbers or links provided?	Not Applicable	
If publicly available data were reused, provide the respective **data citations in the reference list**.	Not Applicable	
